# Design, Synthesis and Biological Evaluation of Novel and Potent Protein Arginine Methyltransferases 5 Inhibitors for Cancer Therapy

**DOI:** 10.3390/molecules27196637

**Published:** 2022-10-06

**Authors:** Yixuan Tang, Shihui Huang, Xingxing Chen, Junzhang Huang, Qianwen Lin, Lei Huang, Shuping Wang, Qihua Zhu, Yungen Xu, Yi Zou

**Affiliations:** 1 Department of Medicinal Chemistry, School of Pharmacy, China Pharmaceutical University, Nanjing 210009, China; 2 Jiangsu Key Laboratory of Drug Design and Optimization, School of Pharmacy, China Pharmaceutical University, Nanjing 210009, China

**Keywords:** epigenetic, PRMT5 inhibitors, THIQ, cancer

## Abstract

Protein arginine methyltransferases 5 (PRMT5) is a clinically promising epigenetic target that is upregulated in a variety of tumors. Currently, there are several PRMT5 inhibitors under preclinical or clinical development, however the established clinical inhibitors show favorable toxicity. Thus, it remains an unmet need to discover novel and structurally diverse PRMT5 inhibitors with characterized therapeutic utility. Herein, a series of tetrahydroisoquinoline (THIQ) derivatives were designed and synthesized as PRMT5 inhibitors using GSK-3326595 as the lead compound. Among them, compound 20 (IC_50_: 4.2 nM) exhibits more potent PRMT5 inhibitory activity than GSK-3326595 (IC_50_: 9.2 nM). In addition, compound 20 shows high anti-proliferative effects on MV-4-11 and MDA-MB-468 tumor cells and low cytotoxicity on AML-12 hepatocytes. Furthermore, compound 20 possesses acceptable pharmacokinetic profiles and displays considerable in vivo antitumor efficacy in a MV-4-11 xenograft model. Taken together, compound 20 is an antitumor compound worthy of further study.

## 1. Introduction

Methylated arginine is a post-translational modification of proteins that exists in many cellular processes including transcription, DNA damage response, stem cell function, epigenetically mediated gene expression and immune response [1,2]. Arginine methylation is regulated by protein arginine methyltransferases (PRMTs). PRMTs transfer methyl group from S-adenosyl-L-methionine (SAM) to the substrate arginine side chain. PRMTs can be categorized into three subtypes: Type I PRMTs (PRMT1, PRMT2, PRMT3, CARM1/PRMT4, PRMT6, and PRMT8) catalyze formation of ω-NG-monomethyl-arginine (MMA) and ω-NG, NG-asymmetric dimethyl-arginine (aDMA), Type II PRMTs (PRMT5 and PRMT9) catalyze formation of MMA and ω-NG, N’G-symmetric dimethyl arginine (sDMA), whereas type III PRMT (PRMT7) catalyzes only formation of MMA [3,4,5].

PRMT5 belongs to the predominant type II PRMTs, which is found in nearly all eukaryotic species [6]. The N-terminal domain of PRMT5 adopts a triosephosphate isomerase barrel structure, which forms a stable complex with methylosome protein 50 (MEP50). MEP50, as the most important interacting partner of PRMT5, is essential for the catalytic activity of PRMT5 [7]. A variety of intracellular substances was methylated by PRMT5, including histone H4 residue Arg3 (H4R3) and H3 residue Arg8(H3R8), which mediated various cellular processes such as transcriptional repression [8,9]. PRMT5 overexpression is involved in the proliferation and survival of numerous different cancers, including colorectal, lung, ovarian, prostate, and pancreatic cancers, and lymphoma, leukemia, and glioblastoma [10,11,12,13,14,15]. Interestingly, it is reported that shPRMT5 or PRMT5 inhibitors motivated cGas/ STING and NLRC5 pathway and repressed MYC downstream genes, which provided possibility to the combination therapy of PD-1 and PRMT5 inhibitors [16,17,18]. Consequently, targeting PRMT5 is being pursued as a new cancer therapeutic strategy.

**Figure 1:**
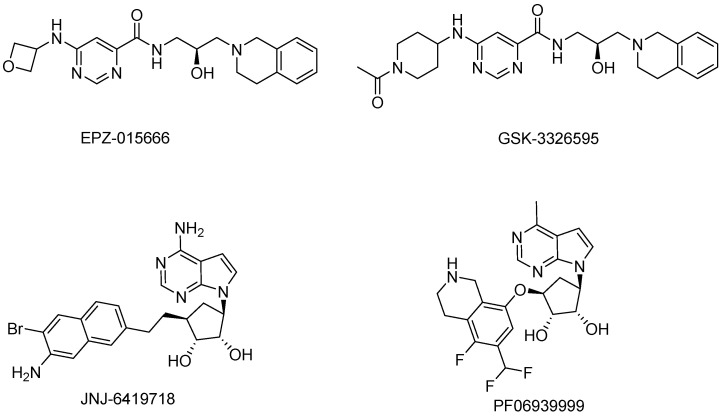
Chemical structures of some representative PRMT5 inhibitors.

In the past few years, tremendous efforts from academia and pharmaceutical companies have been made to discover and develop PRMT5 inhibitors, and several PRMT5 inhibitors are currently in clinical trials (Figure 1). As the active site of the enzyme consists of an SAM-binding pocket and a substrate-binding pocket, the mechanism of action of the clinical PRMT5 inhibitors can be classified into SAM-uncompetitive (GSK-3326595 [19], EPZ015666 [20]) and SAM-competitive (JNJ64619178 [21], PF06939999 [22]) inhibitors. In addition, there are some reports about PRMT5 allosteric inhibition, covalent inhibition and PROTACS [23,24,25]. GSK-3326595 from GlaxoSmithKline is an SAM-uncompetitive PRMT5 inhibitor, which has good brain permeability and anti-tumor effect, and is currently in clinical phase I/II (NCT04676516). Due to the lack of selectivity for tumor cells and normal cells, however, GSK-3326595 caused grade 3 or 4 adverse events in clinical studies. Therefore, the search for safe and effective PRMT5 inhibitors remains a pressing task.

In the present study, we present the design, synthesis and biological evaluation of a series of PRMT5 inhibitors based on the co-crystal structure of EPZ015666 and PRMT5. Among them, compound **20** displays high inhibitory activity both in enzymatic and cellular assays, which is better than GSK-3326595. Furthermore, compound **20** demonstrates potent antitumor in vivo efficacy, making further progress as a potent small molecule inhibitor targeting PRMT5.

## 2. Results and Discussion

### 2.1. Structure-Based Design of Novel PRMT5 Inhibitors

**Figure 2:**
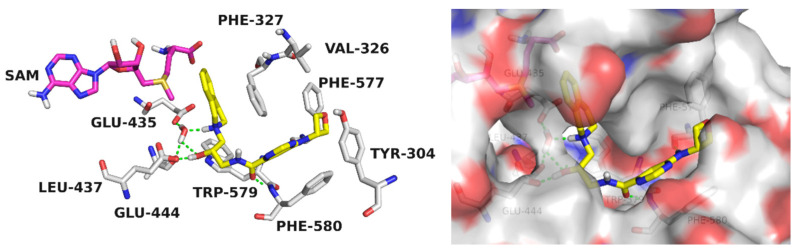
Crystal structure of the PRMT5−EPZ-015666 complex (PDB code: 4X61). Protein surface is shown in gray.

Our approach to design PRMT5 inhibitors took inspiration from the co-crystal structure of EPZ015666 and PRMT5 protein (PDB code: 4X61) (Figure 2). The computer modeling displays a key cation–π interaction between the benzene ring of THIQ and the cofactor SAM, helping to maintain high binding affinity and selectivity for PRMT5. Notably, THIQ also forms a potential π–π stacking interaction with Phe327 and the tertiary nitrogen atom of the THIQ ring system forms a key hydrogen bond, with Glu435 and Leu437 mediated by water molecule. We also noticed that the aminopyrimidine region is situated at the hydrophobic site of the substrate-binding pocket, surrounded by Tyr304, Val326, Phe327, Phe577 and Phe580. Therefore, increasing the volume of the aromatic ring in this area might be advantageous to strengthening the π–π or/and hydrophobic interaction with residues in the active site, thereby enhancing the binding affinity between the compound and PRMT5.

### 2.2. Evaluation of PRMT5 Enzymatic Activities

We initially attempted replacing 1-(4-(pyrimidin-4-ylamino) piperidin-1-yl) ethan-1-one with the carbazole ring. Using a radioactivity-based assay monitoring the transfer of the methyl group from ^3^H-SAM to peptide substrate, we found that compound **1** displays similar PRMT5 inhibitory activity compared with GSK-3326595. However, the structure–activity study of R^1^ and R^2^ did not significantly improve the activity (Table 1).

It is noteworthy that the inhibitory activity could be maintained when the carbazole moiety was replaced by less rigid tetrahydrocarbazole ring, with an IC_50_ of 18.6 ± 0.9 nM (compound **7**). No dramatic change is observed in inhibitory activity upon increasing or decreasing the size of ring A. The substituent R^1^ on the ring A were further investigated, and unfortunately, when R^1^ is methyl or ethyl, the PRMT5 inhibitory activity of the compound **11** and **12** decreases slightly (Table 2).

**Figure 3:**
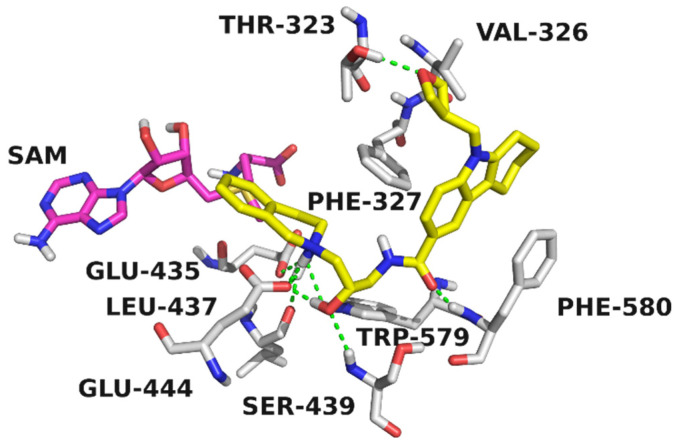
Compound **20** docked into X-ray structure of PRMT5 (PDB code:4X61).

Then we explored the effects of substituent R^2^ on PRMT5 inhibition and expected to enhance the interaction between the pocket defined by Thr323, Val326 and Phe327 and target compounds by introducing different hydrophobic groups (**13–20**). Surprisingly, compounds with the bulkier n-propyl, cyclobutyl and oxetane groups yield excellent PRMT5 inhibitory activities, and compounds **15** and **20** display the most potent inhibitory activity on PRMT5 with IC5_0_ of 4.2 nM. Modeling of the binding pose of **20** was performed by manual ligand building from the crystal structure of the complex of PRMT5 and EPZ-015666 (PDB code: 4X61)(Figure 3). The result shows that the 2,3,4,9-tetrahydro-1*H*-carbazole fragment, which mimics the N-(oxetan-3-yl) pyrimidin-4-amine moiety in EPZ015666, is situated at the hydrophobic site of the substrate-binding pocket, and the oxetan-3-ylmethyl moiety is extended to the small hydrophobic region and undergoes a hydrogen-bonding interaction with Thr323. Finally, we investigated the effects of the tetrahydropyrido [4,3-*b*] indole moiety and its substituent R^1^ on PRMT5 inhibition. However, whether the tetrahydrocarbazole moiety is replaced by tetrahydropyrido[4,3-*b*]indole or substituent R^1^ is replaced by alkyl, the outcome is unfavorable for PRMT5 inhibitory activity.

### 2.3. In Vitro Anti-Proliferative Activity Evaluation

MV-4-11 and MDA-MB-468 cell lines were used to evaluate the anti-proliferative activities of the selected target compounds (CellTiter-Glo assay), and AML-12 hepatocytes were selected to evaluate the toxicity of the compounds. As shown in Table 3, compound **20** exhibits stronger anti-proliferative activities than **GSK-3326595** in MV-4-11 and MDA-MB-468 cell lines. however, it has almost no toxicity compared to normal AML-12 hepatocytes.

### 2.4. Cellular Thermal Shift Assay (CETSA)

**Figure 4:**
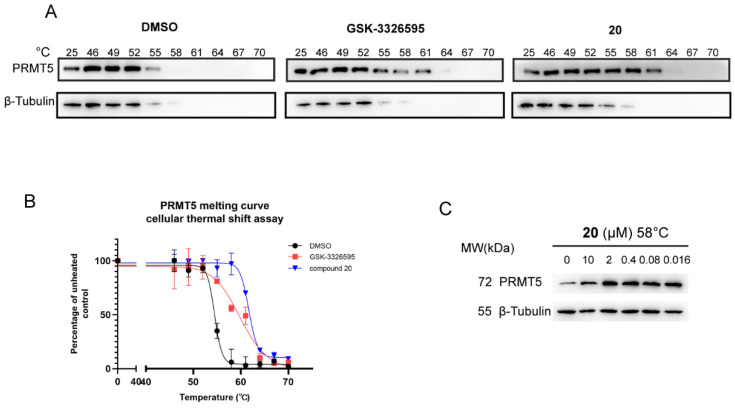
(**A**) MV-4-11 cell lines were treated for 18 h with 1 μM GSK-3326595, and compound **20** showed stabilization of PRMT5 in cellular thermal shift analysis in whole-cell lysates. (**B**) The relative strength of PRMT5 signal on the Western blots was measured by densitometry. The melting temperature for PRMT5 was shifted by 5.5 °C and 7.2 °C. All data were analyzed using a Boltzmann sigmoidal fit. Each point plotted represents the mean of three replicates for each temperature; error bars denote ±SD. (**C**) The binding of **20** inhibits the degradation of PRMT5 at 58 °C in a dose-dependent manner.

To confirm the target engagement in cells, compound **20** and **GSK-3326595** were selected and evaluated using an MV-4-11 cell line by CETSA [26,27]. As showed in Figure 4, we measured the PRMT5 melting curve and facilitated the temperature selection, as the PRMT5 melting point of **GSK-3326595** and compound **20** increased by 5.5 °C and 7.2 °C compared to the DMSO vehicle, indicating the thermal stabilization of proteins upon ligand binding. Moreover, the binding of **20** also inhibits the degradation of PRMT5 at 58 °C in a dose-dependent manner. All the results demonstrate better binding potency in a cellular context.

### 2.5. Characterization of Cell Methylation and Proliferation

**Figure 5:**
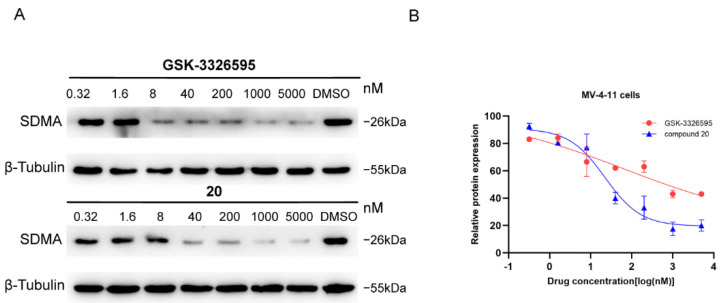
(**A**) Effect of diluent of **GSK-3326595** and compound **20** by five-fold concentration gradient protein expression of MV-4-11 cancer cells. Western blot analysis of PRMT5 substrate sDMA, as indicated in MV-4-11 cancer cells treated with the indicated compounds for 96 h. β-Tubulin was used as a loading control. (**B**) The relative strength of sDMA signal on the Western blots was measured by densitometry. The data are shown as the mean ± SD of three independent experiments.

To further evaluate if compound **20** exhibits anti-proliferative activity depending on PRMT5 inhibition, we analyzed the effect of compound 20 on PRMT5 substrate sDMA protein expression in MV-4-11 cell, with high expression in PRMT5. As shown in Figure 5, the treatment of compound 20 leads to a concentration-dependent decrease in sDMA, indicating its better PRMT5 inhibition than GSK-3326595. All the cellular data suggests that compound **20** inhibits cell proliferation through inhibiting PRMT5 activity.

### 2.6. In Vivo Pharmacokinetic Study

Considering the desirable enzymatic potency, as well as the potent anti-proliferative activity in several cancer cell lines, compound **20** was selected and evaluated for its pharmacokinetics (PK) data in male ICR mice at 2 mg/kg and 10 mg/kg for intravenous (iv) and oral (po) administration, respectively. The resulting data (Tables S1 and S2) are summarized in Table 4. It reveals that compound **20** exhibits an acceptable PK profile with a T_1/2_ of 6.06 h, a modest drug exposure in blood (AUC_0−∞_ values after oral treatment of 10 mg/kg are 747 h·ng/mL) and an acceptable oral bioavailability of 14.5%.

### 2.7. Antitumor Effect of 20 In Vivo

**Figure 6:**
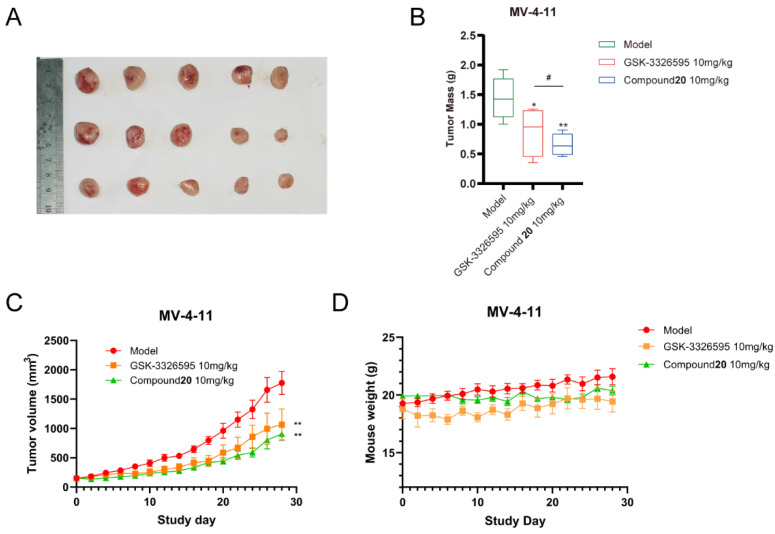
Effects of **GSK-3326595** (10 mg·kg^−1^, iv) and compound **20** (10 mg·kg^−1^, iv) treatment on the tumorigenicity of MV-4-11 cells in vivo. (**A**) The resulting tumors excised from the animals after treatment. (**B**) The tumor masses for three groups of animals were compared, and each histogram represents the Mean ± SD of 5 mice. * *p* < 0.05 vs. model, ** *p* < 0.01, ^#^ *p* < 0.05 vs. **GSK-3326595**. (**C**) The nude mouse body weight was measured every 2 days. (**D**) The tumor volumes of the nude mice were measured and calculated once every 2 days.

Based on the excellent enzymatic and anti-proliferative activities of compound **20** in vitro, we then evaluated antitumor activities in vivo in an MV-4-11 xenograft mouse model. **GSK-3326595** and compound **20** were administered by intraperitoneal injection twice daily (BID) for 28 consecutive days. As shown in Figure 6, compound **20** demonstrates its potent antitumor efficacy at a dose of 10 mg·kg^−1^, and the tumor suppression effect of **20** (TGI: 47.6%) is more effective than that of the positive control **GSK-3326595** (TGI: 39.3%). It is noteworthy that no significant weight fluctuations were observed, and compound 20 was well-tolerated with no mortality.

**Figure 7:**
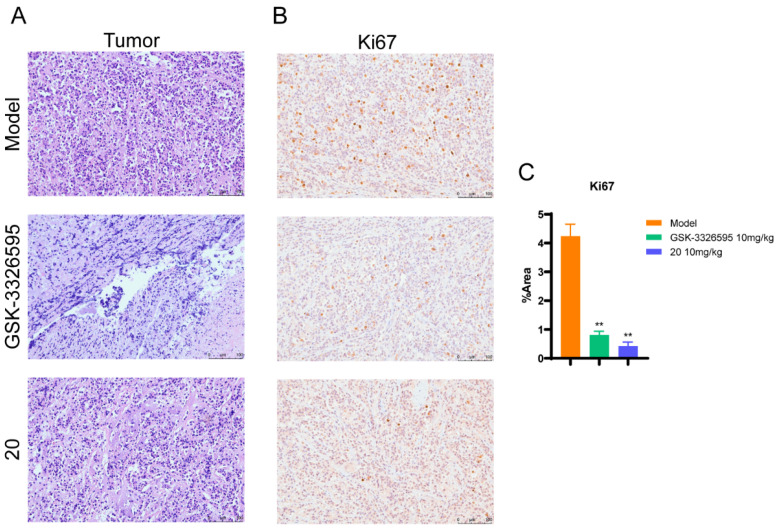
H&E staining and immunohistochemical analysis of Ki-67 expression in vivo. (**A**) Hematoxylin-eosin staining (H&E) analysis of tumor of **GSK-3326595** and compound **20** treatment. (**B**) Immunohistochemical analysis of Ki67 in vivo. (**C**) Percentage of immunohistochemically stained dots. * *p* < 0.05 vs. model, ** *p* < 0.01.

The expression of Ki-67 is positively correlated to the malignancy of tumors [28]. H&E staining shows the malignancy of the tumor and drug prognostic effect [29]. Therefore, H&E staining and Ki-67 immunohistochemistry were further used to gain insight into the changes in the structure and appearance of cancerous cellular structures after compound **20** administration. As shown in Figure 7, all administration groups cause a decrease in the population of tumor cells, and morphological features including cell shrinkage, condensation and margination of nuclear chromatin are observed by microscopy. Meanwhile, the administration group also down-regulates the expression of Ki-67, indicating the decrease in cell proliferation.

### 2.8. Expression of sDMA In Vivo

**Figure 8:**
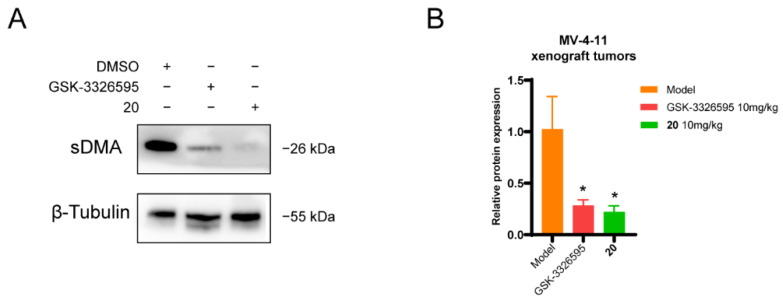
PRMT5 substrate sDMA expression of **GSK-3326595** (10 mg·kg^−1^, iv) and compound **20** (10 mg·kg^−1^, iv) treatment for the tumorigenicity of MV-4-11 cells in vivo. (**A**) Western blot analysis of sDMA as indicated in MV-4-11 xenograft tumors treated with the indicated compounds for 28 days. (**B**) The relative strength of sDMA signal on the Western blots was measured by densitometry. The data are shown as the mean ± SD of three independent experiments. * *p* < 0.05 vs. control.

Subsequently, we investigated the effects of compound **20** on the expression of the PRMT5 substrate sDMA in vivo (Figure 8). Similarly, the expression of sDMA was decreased after the treatment of compound **20**, which was in line with its antitumor activity in vivo.

### 2.9. Chemistry

**Scheme 1:**
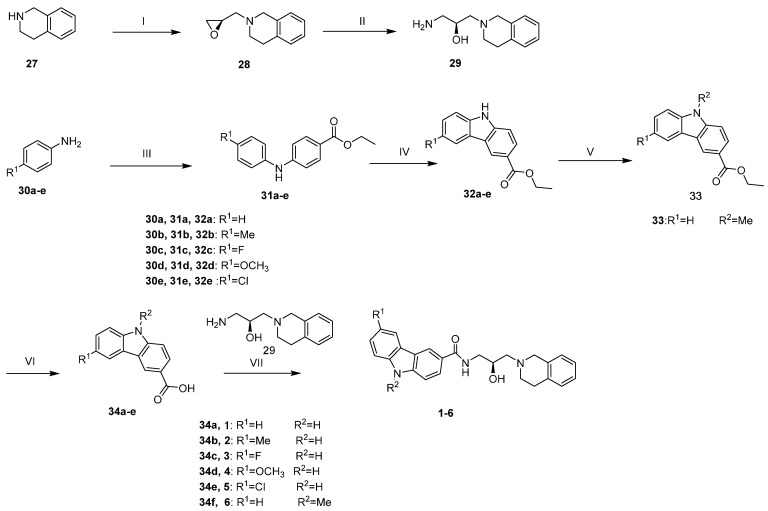
Reagents and conditions: (I) (*R*)-epichlorohydrin, THF, KF, 0°C~rt; (II) NH_3_H_2_O, EtOH, 80 °C; (III) ethyl 4-bromobenzoate, Pd(OAc)_2_, BINAP, K_2_CO_3_, Toluene, 120 °C; (IV) Pd(OAc)_2_, AcOH, 120 °C; (V) NaH, MeI, DMF; (VI) KOH, EtOH, H_2_O, 90 °C; (VII) HATU, DIEA, DCM.

The preparation of compounds **1–6** was described in Scheme 1. Initially, the commercially available 1,2,3,4-tetrahydroisoquinoline was substituted by (*R*)-epichlorohydrin to afford compound **28**, which was ring-opened by ammonolysis of ammonia water to give compound **29**. Meanwhile, compounds **30a**–**30e** were reacted with ethyl 4-bromobenzoate to afford compounds **31a**–**31e**, which were catalyzed by Pd(OAc)_2_ to afford compounds **32a**–**32e**. **32a** reacted with MeI by nucleophilic substitution to afford compound **33**. Subsequently, 32a-32e and 33 were hydrolyzed by KOH to obtain compounds **34a**–**34f**. As a result, compounds **1–6** were synthesized from carboxylic acid **34a**–**34f** and the intermediate **29**.

**Scheme 2:**
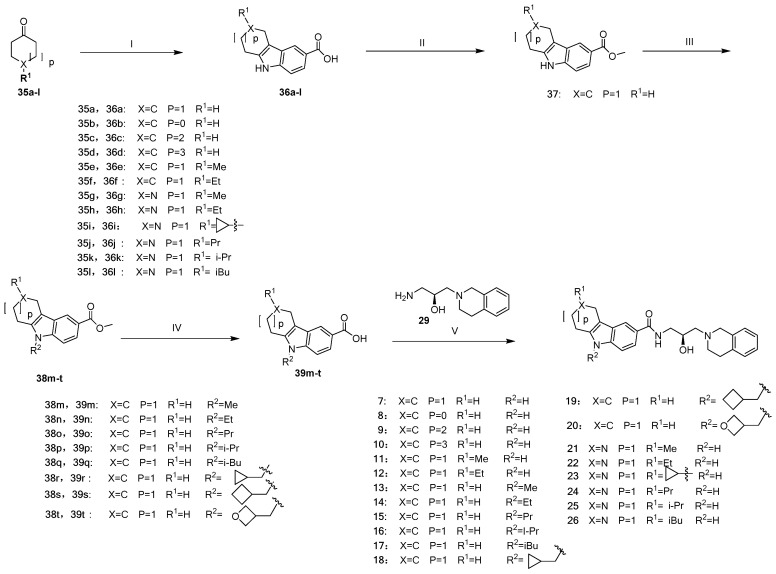
Reagents and conditions: (I) H_2_SO_4_, 4-hydrazinylbenzoic acid, H_2_O, 100 °C; (II) H_2_SO_4_, MeOH, 65 °C; (III) NaH, halogenated alkane, DMF, 0-r.t; (IV) KOH, EtOH, H_2_O, 90 °C; (V) HATU, DIEA, DCM.

The synthetic routes of compounds **7**–**26** were shown in Scheme 2. **35a**–**35l** were reacted with p-carboxyphenylhydrazine to afford compounds **36a**–**36l**, which were subsequently reacted with MeOH in the presence of H_2_SO_4_ to give compound **37**. R^2^ was introduced by nucleophilic substitution using halogenated alkanes to yield compounds **38m**–**38t**, which were hydrolyzed by LiOH to afford compounds **39m**–**39t**. Finally, the target compounds **7–26** were prepared from the corresponding carboxylic acid and the key intermediates **29**.

## 3. Experimental Sections

### 3.1. General Chemistry

All solvents and reagents were purchased from commercial vendors and used without further purification. The progress of the reaction was monitored by TLC on pre-coated silica gel plates (silica gel 60 F254), and spots were observed by UV light (λ_max_ = 254 or 365 nm) or other suitable stain. ^1^H (300 or 400 MHz) and ^13^C NMR (75 MHz or 101 MHz) spectra were recorded by Bruker spectrometer with TMS as internal standard. Coupling constants (J) are expressed in hertz (Hz). Ordinary and high-resolution mass spectra were obtained by ESI-MS. The purification of compounds was conducted by flash column chromatography (silica gel 200–300 mesh). The purity of all target compounds was > 95%, as determined by HPLC (BDS Hypersil C18, λ = 254 nm).

#### 3.1.1. General Procedure for the Synthesis of Compounds **28** and **29**

(*R*)-2-(oxiran-2-ylmethyl)-1,2,3,4-tetrahydroisoquinoline (**28**).

To a mixture of **27** (1.00 g, 7.50 mmol) in THF (10 mL) at 0°C was successively added KF (0.87 g, 15 mmol) and (*S*)-oxiran-2-ylmethyl 3-nitrobenzenesulfonate (2.20 g, 15 mmol). The reaction mixture was stirred at 0 °C for 1 h, and then reacted at 25 °C for 16 h. The mixture was filtered to remove the solid, and washed with of THF (5 mL) twice. The filtrate was concentrated and purified by column chromatography (PE/EA: 5/1) to obtain 0.86 g of pale-yellow oil with a yield of 61.0%; ^1^H NMR (300 MHz, Chloroform-*d*) *δ* 7.15–7.08 (m, 3 H), 7.06–7.01 (m, 1 H), 3.86–3.66 (m, 2 H), 3.25–3.17 (m, 1 H), 2.99–2.87 (m, 4 H), 2.86–2.76 (m, 2 H), 2.56 (dd, *J* = 5.0, 2.7 Hz, 1 H), 2.50–2.40 (m, 1 H); MS (ESI): 190.1.

(*S*)-1-amino-3-(3,4-dihydroisoquinolin-2(1*H*)-yl) propan-2-ol (**29**).

A mixture of **28** (1.00 g, 26.0 mmol) in NH_4_OH (10 mL) was refluxed at 80 °C for 3 h. The reaction mixture was allowed to cool to 25 °C and concentrated under reduced pressure. The crude product was purified using column chromatography (DCM/MeOH: 15/1) to obtain the titled compound (0.37 g, 34.0%) as a pale-yellow oil; ^1^H NMR (400 MHz, Chloroform-*d*) *δ* 7.18–7.09 (m, 3 H), 7.05–6.98 (m, 1 H), 3.85–3.76 (m, 2 H), 3.62 (d, *J* = 14.9 Hz, 1 H), 2.97–2.81 (m, 4 H), 2.78–2.64 (m, 2 H), 2.61–2.46 (m, 2 H); MS (ESI): 207.1.

#### 3.1.2. General Procedure for the Synthesis of Compound **31a**–**e**

A mixture of **30a**–**e** (2.80 mmol, 1 eq), ethyl 4-bromobenzoate (2.80 mmol, 1 eq), Pd(OAc)2 (0.14 mmol, 0.05 eq), BINAP (0.14 mmol, 0.05 eq) and K_2_CO_3_ (3.0 mmol, 1.07 eq) in toluene (10 mL) was refluxed under an atmosphere of N_2_ for 6 h. The reaction mixture was allowed to cool to rt and concentrated under reduced pressure. The crude product was purified using a column chromatography cation to obtain compound **31a**–**e**.

Ethyl 4-(phenylamino) benzoate (**31a**).

White solid; yield: 83.6%; ^1^H NMR (300 MHz, Chloroform-*d*) *δ* 7.99–7.92 (m, 2 H), 7.40–7.32 (m, 2 H), 7.24–7.18 (m, 2 H), 7.13–7.05 (m, 1 H), 7.05–6.99 (m, 2 H), 6.07 (s, 1 H), 4.37 (q, *J* = 7.1 Hz, 2 H), 1.40 (t, *J* = 7.1 Hz, 2 H); MS (ESI): 242.1.

Ethyl 4-(p-tolylamino) benzoate (**31b**).

White solid; yield: 59.5%; ^1^H NMR (300 MHz, Chloroform-*d*) *δ* 7.95–7.89 (m, 2 H), 7.20–7.14 (m, 2 H), 7.13–7.07 (m, 3 H), 6.97–6.91 (m, 2 H), 4.35 (q, *J* = 7.1 Hz, 2 H), 2.36 (s, 3 H), 1.39 (t, *J* = 7.1 Hz, 3 H); MS (ESI): 256.1.

Ethyl 4-((4-fluorophenyl) amino) benzoate (**31c**).

White solid; yield: 81.4%; ^1^H NMR (300 MHz, Chloroform-*d*) *δ* 7.95 (d, *J* = 8.2 Hz, 2 H), 7.20 (d, *J* = 4.9 Hz, 1 H), 7.18 (d, *J* = 4.6 Hz, 1 H), 7.08 (t, *J* = 8.4 Hz, 2 H), 6.93 (d, *J* = 8.2 Hz, 2 H), 4.38 (q, *J* = 6.9 Hz, 2 H), 1.42 (t, *J* = 7.1 Hz, 3 H); MS (ESI): 260.1.

Ethyl 4-((4-methoxyphenyl) amino) benzoate (**31d**).

White solid; yield: 90.8%; ^1^H NMR (300 MHz, Chloroform-*d*) *δ* 7.90 (d, *J* = 8.8 Hz, 2 H), 7.16 (d, *J* = 8.7 Hz, 2 H), 7.00–6.89 (m, 2 H), 6.84 (d, *J* = 8.7 Hz), 4.35 (q, *J* = 7.1 Hz, 2 H), 3.84 (s, 2 H), 1.39 (t, *J* = 7.1 Hz, 3 H); MS (ESI): 272.1.

Ethyl 4-((4-chlorophenyl) amino) benzoate (**31e**).

White solid; yield: 84.8%; ^1^H NMR (300 MHz, Chloroform-*d*) *δ* 8.03–7.89 (m, 2 H), 7.31–7.27 (m, 2 H), 7.12–7.06 (m, 2 H), 6.99–6.93 (m, 2 H), 4.34 (q, *J* = 7.1 Hz, 2 H),1.38 (t, *J* = 7.1 Hz, 3 H); MS (ESI):276.1.

#### 3.1.3. General Procedure for the Synthesis of Compound **32a**–**e**

A mixture of **31a**–**e** (1.24 mmol, 1 eq), Pd(OAc)_2_ (1.37 mmol, 1 eq) in AcOH (8 mL) was refluxed under an atmosphere of N_2_ for 1 h. The reaction mixture was allowed to cool to rt and concentrated under reduced pressure. The crude product was purified using a column chromatography cation to obtain compound **32a**–**e**.

Ethyl 9*H*-carbazole-3-carboxylate (**32a**).

White solid; yield: 64.0%; ^1^H NMR (300 MHz, Chloroform-*d*) *δ* 8.85 (d, *J* = 1.4 Hz, 1 H), 8.43 (s, 1 H), 8.21–8.10 (m, 2 H), 7.51–7.42 (m, 3 H), 7.35–7.30 (m, 1 H), 4.48 (q, *J* = 7.1 Hz, 2 H), 1.49 (t, *J* = 7.1 Hz, 3 H); MS (ESI): 240.1.

Ethyl 6-methyl-9*H*-carbazole-3-carboxylate (**32b**).

White solid; yield: 55.0%; ^1^H NMR (300 MHz, DMSO-*d*6) *δ* 11.56 (s, 1 H), 8.80 (s, 1 H), 7.98 (dd, *J* = 8.6, 1.6 Hz, 1 H), 7.95–7.81 (m, 1 H), 7.51 (d, *J* = 8.6 Hz, 1 H), 7.45 (d, *J* = 8.8 Hz, 1 H), 7.07 (dd, *J* = 8.8, 2.5 Hz, 1 H), 4.35 (q, *J* = 7.1 Hz, 2 H), 3.87 (s, 3 H), 1.37 (t, *J* = 7.1 Hz, 3 H); MS (ESI): 254.1.

Ethyl 6-fluoro-9*H*-carbazole-3-carboxylate (**32c**).

White solid; yield: 52.0%; ^1^H NMR (300 MHz, DMSO-*d*6) *δ* 11.82 (s, 1 H), 8.85 (s, 1 H), 8.17 (dd, *J* = 9.4, 2.5 Hz, 1 H), 8.06 (dd, *J* = 9.4, 2.4 Hz, 1 H), 7.63–7.54 (m, 2 H), 7.37–7.30 (m, 1 H), 4.38 (q, *J* = 7.1 Hz, 2 H), 1.40 (t, *J* = 7.1 Hz, 3 H); MS (ESI): 258.1.

Ethyl 6-methoxy-9*H*-carbazole-3-carboxylate (**32d**).

White solid; yield: 68.5%; MS (ESI): 270.1.

Ethyl 6-chloro-9*H*-carbazole-3-carboxylate (**32e**).

White solid; yield: 80.6%; ^1^H NMR (300 MHz, DMSO-*d*6) *δ* 11.92 (s, 1 H), 8.88 (s, 1 H), 8.43 (d, *J* = 2.0 Hz, 1 H), 8.07 (dd, *J* = 8.6, 1.7 Hz, 1 H), 7.59 (dd, *J* = 8.6, 5.3 Hz, 2 H), 7.48 (dd, *J* = 8.6, 2.0 Hz, 1 H), 4.38 (q, *J* = 7.1 Hz, 2 H), 1.40 (t, *J* = 7.1 Hz, 3 H); MS (ESI): 274.1.

#### 3.1.4. General Procedure for the Synthesis of Compound **33**

**32a** (300 mg, 1.31 mmol) in DMF (5 mL) was stirred at 0 °C for 5 min. NaH (90 mg, 3.75 mmol) was added and reacted at 25 °C for 0.5 h, MeI (264 mg, 1.86 mmol) was added dropwise at 0 °C, followed by reaction at 25 °C for 3 h TLC (DCM/MeOH: 50/1) to monitor the completion of the reaction. H_2_O (20 mL) was added to quench the reaction. EA (10 mL × 3) extracted with EA (10 mL × 3), washed with saturated NaCl (10 mL), dried over anhydrous Na_2_SO_4_, filtrated, and the filtrate was dried and purified by column chromatography (DCM/MeOH: 50/1) to obtain the titled compound (45 mg, 47.3%) as a white solid; MS (ESI): 254.1.

#### 3.1.5. General Procedure for the Synthesis of Compounds **34a**–**f**

A mixture of **32a**–**e** or **33** (0.38 mmol, 1 eq), KOH (1.13 mmol, 3 eq) in EtOH (5 mL) and H_2_O (1 mL) was refluxed under an atmosphere of N_2_ for 3 h. The reaction mixture was allowed to cool to rt and concentrated under reduced pressure. 5 mL H_2_O was added, 1 mL 2N (HCL) was added to adjust the pH to about 3, and suction filtration to obtain the compounds **34a**–**f**.

9*H*-carbazole-3-carboxylic acid (**34a**).

White solid; yield: 86.1%; MS (ESI):212.1.

6-methyl-9*H*-carbazole-3-carboxylic acid (**34b**).

White solid; yield: 81.8%; ^1^H NMR (300 MHz, DMSO-*d*6) *δ* 11.58 (s, 1 H), 8.70 (d, *J* = 1.5 Hz, 1 H), 8.01 (s, 1 H), 7.97 (dd, *J* = 8.5, 1.7 Hz, 1 H), 7.50 (d, *J* = 8.5 Hz, 1 H), 7.43 (d, *J* = 8.2 Hz, 1 H), 7.26 (dd, *J* = 8.3, 1.3 Hz, 1 H), 2.47 (s, 3 H); MS (ESI): 226.1.

6-fluoro-9*H*-carbazole-3-carboxylic acid (**34c**).

White solid; yield: 56.3%; ^1^H NMR (300 MHz, DMSO-*d*6) *δ* 12.63 (s, 1 H), 11.75 (s, 1 H), 8.82 (s, 1 H), 8.12 (dd, *J* = 9.4, 2.6 Hz, 1 H), 8.02 (dd, *J* = 8.6, 1.7 Hz, 1 H), 7.54 (dd, *J* = 8.8, 4.7 Hz, 2 H), 7.34–7.24 (m, 1 H); MS (ESI): 230.1.

6-methoxy-9*H*-carbazole-3-carboxylic acid (**34d**).

White solid; yield: 86.5%; ^1^H NMR (300 MHz, DMSO-*d*6) *δ* 11.52 (s, 1 H), 8.80 (s, 1 H), 7.99 (d, *J* = 8.6 Hz, 1 H), 7.86 (s, 1 H), 7.53–7.43 (m, 2 H), 7.09 (d, *J* = 11.2 Hz, 1 H), 3.88 (s, 3 H); MS (ESI): 242.1.

6-chloro-9*H*-carbazole-3-carboxylic acid (**34e**).

White solid; yield: 88.7%; ^1^H NMR (300 MHz, DMSO-*d*6) *δ* 11.90 (s, 1 H), 8.86 (s, 1 H), 8.40 (d, *J* = 2.0 Hz, 1 H),8.06 (dd, *J* = 8.5, 1.6 Hz,1 H), 7.60 (s, 1 H), 7.57 (s, 1 H), 7.47 (dd, *J* = 8.6, 2.0 Hz, 1 H); MS (ESI): 246.0.

9-methyl-9*H*-carbazole-3-carboxylic acid (**34f**).

White solid; yield: 75.0%; ^1^H NMR (300 MHz, DMSO-*d*6) *δ* 12.5 (s, 1 H), 8.82 (d, J = 1.5 Hz, 1 H), 8.31 (d, *J* = 7.7 Hz, 1 H),8.10 (dd, *J* = 8.6, 1.7 Hz, 1 H), 7.71 (d, *J* = 3.9 Hz, 1 H), 7.56 (t, *J* = 7.7 Hz, 1 H), 7.30(t, *J* = 7.4 Hz, 1 H), 3.96 (s, 3 H). MS (ESI): 226.1.

#### 3.1.6. General Procedure for the Synthesis of Compounds **36a**–**l**

A mixture of **35a**–**l** (6.49 mmol, 1.3 eq), 4-hydrazinylbenzoic acid (5.00 mmol, 1 eq) in 10% H_2_SO_4_(20 mL) was refluxed under an atmosphere of N_2_ for 3 h. The reaction mixture was allowed to cool to rt the solid was precipitated and filtrated, and the filter cake with 10 mL of H_2_O, and dried to obtain the compound **36a**–**l**.

2,3,4,9-tetrahydro-1*H*-carbazole-6-carboxylic acid (**36a**).

Gray solid; yield: 75.0%; ^1^H NMR (300 MHz, DMSO-*d*6) *δ* 12.32 (s, 1 H), 11.08 (s, 1 H), 8.04 (s, 1 H), 7.66 (dd, *J* = 8.5, 1.6 Hz, 1 H), 7.31 (d, *J* = 8.5 Hz, 1 H), 2.80–2.60 (m,4 H), 1.96–1.76 (m, 4 H); MS (ESI): 216.1.

1,2,3,4-tetrahydrocyclopenta[*b*]indole-7-carboxylic acid (**36b**).

Gray solid; yield: 68.0%; ^1^H NMR (300 MHz, DMSO-*d*6) *δ* 11.24 (s, 1 H), 8.03 (s, 1 H), 7.66 (dd, *J* = 8.5, 1.6 Hz, 1 H), 7.36 (d, *J* = 8.5 Hz, 1 H), 2.86 (t, *J* = 7.0 Hz, 2 H), 2.79 (t, *J* = 6.9 Hz, 2 H), 2.49–2.30 (m, 2 H); MS (ESI): 202.1.

5,6,7,8,9,10-hexahydrocyclohepta[*b*]indole-2-carboxylic acid (**36c**).

Gray solid; yield: 97.8%; ^1^H NMR (300 MHz, DMSO-*d*6) *δ* 11.11 (s, 1 H), 8.07 (s, 1 H), 7.64 (d, *J* = 8.3 Hz, 1 H), 7.30 (d, *J* = 8.4 Hz, 1 H), 2.85 (t, *J* = 5.6 Hz, 2 H), 2.78(t, *J* = 5.6 Hz, 2 H), 1.93–1.82 (m, 2 H), 1.79–1.64 (m, 4 H); MS (ESI):230.1.

6,7,8,9,10,11-hexahydro-5*H*-cycloocta[*b*]indole-2-carboxylic acid (**36d**).

Gray solid; yield: 56.3%; ^1^H NMR (400 MHz, DMSO-*d*6) *δ* 12.32 (s, 1 H), 11.08 (s, 1 H), 8.06 (s, 1 H), 7.62 (d, *J* = 10.0 Hz, 1 H), 7.29 (d, *J* = 8.5 Hz, 1 H), 2.83 (q, *J* = 5.6 Hz, 4 H), 1.80–1.58 (m, 4 H), 1.52–1.29 (m,4 H); MS (ESI): 243.1.

3-methyl-2,3,4,9-tetrahydro-1*H*-carbazole-6-carboxylic acid (**36e**).

Gray solid; yield: 90.2%; ^1^H NMR (400 MHz, DMSO-*d*6) *δ* 11.07 (s, 1 H), 8.01 (s, 1 H), 7.63 (dd, *J* = 8.5, 1.6 Hz, 1 H), 7.28 (d, *J* = 8.4 Hz, 1 H), 2.84–2.76(m, 1 H), 2.77–2.71 (m, 2 H), 2.22 (dd, *J* = 15.3, 9.5 Hz, 1 H), 1.97–1.82 (m, 2 H), 1.58–1.43 (m, 1 H), 1.10 (d, *J* = 6.5 Hz, 3 H); MS (ESI): 230.1.

3-ethyl-2,3,4,9-tetrahydro-1*H*-carbazole-6-carboxylic acid (**36f**).

Gray solid; yield: 78.6%; ^1^H NMR (400 MHz, DMSO-*d*6) *δ* 11.06 (s, 1 H), 8.03 (s, 1 H), 7.63 (dd, *J* = 8.4, 1.7 Hz, 1 H), 7.28 (dd, *J* = 8.6, 1.4 Hz, 1 H), 2.84 (dd, *J* = 15.4, 4.9 Hz, 1 H), 2.77–2.69 (m, 2 H), 2.22 (dd, *J* = 14.7, 10.0 Hz, 2 H), 1.97 (dd, *J* = 11.5, 6.1 Hz, 1 H), 1.72–1.59(m, 1 H), 1.55–1.37(m, 3 H), 0.99 (t, *J* = 7.3 Hz, 3 H); MS (ESI): 244.1.

2-methyl-2,3,4,5-tetrahydro-1*H*-pyrido[4,3-*b*] indole-8-carboxylic acid (**36g**).

White solid; yield: 8.85%; ^1^H NMR (300 MHz, DMSO-*d*6) *δ* 11.79 (s, 1 H), 11.45 (s, 1 H), 8.11 (s, 1 H), 7.73 (dd, *J* = 8.6, 1.5 Hz,1 H), 7.42 (d, *J* = 8.5 Hz, 1 H), 4.63 (d, *J* = 13.0 Hz, 1 H), 4.29 (dd, *J* = 14.3, 7.3 Hz, 1 H), 3.74–3.62 (m, 1 H), 3.54–3.40 (m, 1 H), 3.33–3.19 (m, 1 H), 3.11–2.98 (m, 1 H), 2.93 (d, *J* = 4.3 Hz, 3 H); MS (ESI): 231.1.

2-ethyl-2,3,4,5-tetrahydro-1*H*-pyrido[4,3-*b*] indole-8-carboxylic acid (**36h**).

White solid; yield: 8.35%; ^1^H NMR (300 MHz, DMSO-*d*6) *δ* 8.20 (d, *J* = 1.5 Hz, 1 H), 7.75 (dd, *J* = 8.6, 1.5 Hz, 1 H), 7.45 (d, *J* = 8.6 Hz, 1 H), 4.72 (d, *J* = 14.5 Hz, 1 H), 4.30 (d, *J* = 14.5 Hz, 1 H), 3.83–3.73 (m, 1 H), 3.50–3.40 (m, 1 H), 3.39–3.28 (m, 2 H), 3.28–3.18 (m, 1 H), 3.17–3.00 (m, 1 H), 1.40 (t, *J* = 7.2 Hz, 3 H); MS (ESI): 245.1.

2-cyclopropyl-2,3,4,5-tetrahydro-1*H*-pyrido[4,3-*b*] indole-8-carboxylic acid (**36i**).

White solid; yield: 19.41%; ^1^H NMR (400 MHz, DMSO-*d*6) *δ* 11.67 (s, 1 H), 11.17 (s, 1 H), 8.18 (s, 1 H), 7.72 (dd, *J* = 8.5, 1.5 Hz, 1 H), 7.40 (d, *J* = 8.5 Hz, 1 H), 4.70 (d, *J* = 14.1 Hz, 1 H), 4.46 (d, *J* = 9.9 Hz, 1 H), 3.83–3.71 (m, 1 H), 3.69–3.59 (m, 1 H), 3.32–3.20 (m, 1 H), 3.08 (d, *J* = 14.8 Hz, 1 H), 1.27 (s, 2 H), 0.92–0.78 (m, 2 H); MS (ESI): 257.1.

2-propyl-2,3,4,5-tetrahydro-1*H*-pyrido[4,3-*b*] indole-8-carboxylic acid (**36j**).

White solid; yield: 9.97%; ^1^H NMR (300 MHz, DMSO-*d*6) *δ* 11.79 (s, 1 H), 11.33 (s, 1 H), 8.19 (s, 1 H), 7.75 (dd, *J* = 8.6, 1.4 Hz, 1 H), 7.42 (s, 1 H), 4.69 (d, *J* = 14.2 Hz, 1 H), 4.31 (dd, *J* = 14.6, 7.2 Hz, 1 H), 3.80–3.68 (m, 1 H), 3.37–3.27 (m, 2 H), 3.25–3.14 (m, 2 H), 3.14–3.00 (m, 1 H), 2.02–1.80 (m, 2 H), 0.98 (t, *J* = 7.3 Hz, 3 H); MS (ESI): 259.1.

2-isopropyl-2,3,4,5-tetrahydro-1*H*-pyrido[4,3-*b*] indole-8-carboxylic acid (**36k**).

White solid; yield: 44.5%; ^1^H NMR (300 MHz, DMSO-*d*6) *δ* 11.66 (s, 1 H), 10.87 (s, 1 H), 8.20 (s, 1 H), 7.72 (d, *J* = 10.1 Hz, 1 H), 7.41 (d, *J* = 8.5 Hz, 1 H), 4.60–4.49 (m, 1 H), 4.33 (dd, *J* = 14.6, 8.8 Hz, 1 H), 3.78–3.61 (m, 1 H), 3.34–3.20 (m, 2 H), 3.10–2.93 (m, 1 H), 1.40 (dd, *J* = 10.8, 6.6 Hz, 6 H); MS (ESI):259.1

2-isobutyl-2,3,4,5-tetrahydro-1*H*-pyrido[4,3-*b*] indole-8-carboxylic acid (**36l**).

White solid; yield: 23.5%; ^1^H NMR (300 MHz, DMSO-*d*6) δ 8.21 (d, *J* = 1.5 Hz, 1 H), 7.75 (dd, *J* = 8.6, 1.7 Hz, 1 H), 7.45 (d, *J* = 8.6 Hz, 1 H), 4.75 (d, *J* = 14.6 Hz, 1 H), 4.35 (d, *J* = 14.7 Hz, 1 H), 3.83–3.73 (m, 1 H), 3.54–3.48 (m, 1 H), 3.48–3.40 (m, 1 H), 3.34–3.19 (m, 1 H), 3.18–3.11 (m, 1 H), 3.10–3.02(m, 1 H), 2.36–2.21 (m, 1 H), 1.03 (t, *J* = 6.9 Hz, 6 H); MS (ESI): 273.2.

#### 3.1.7. General Procedure for the Synthesis of Compound **37**

Methyl 2,3,4,9-tetrahydro-1*H*-carbazole-6-carboxylate (**37**).

A mixture of **36a** (2.2 g, 10.22 mmol) in MeOH (40 mL) and H_2_SO_4_ (2.19 mL, 40.88 mmol) was refluxed at 65 °C for 6 h. The reaction mixture was allowed to cool to rt and concentrated under reduced pressure. H_2_O (30 mL) was added, extracted with EA (10 mL × 3), washed with saturated NaCl (10 mL), dried with anhydrous Na_2_SO_4_, filtrate, and dried to obtain the titled compound (2.3 g, 98.3%) as a white solid; ^1^H NMR (300 MHz, Chloroform-*d*) *δ* 8.22 (s, 1 H), 7.95 (s, 1 H), 7.83 (d, *J* = 8.5 Hz, 1 H), 7.25 (d, *J* = 8.5 Hz, 1 H), 3.93 (s, 3 H), 2.72 (t, *J* = 5.9 Hz, 4 H), 1.97–1.81 (m, 4 H); MS (ESI): 230.1.

#### 3.1.8. General Procedure for the Synthesis of Compounds **38m**–**t**

**37** (1.31 mmol, 1 eq) in DMF (5 mL) was stirred at 0 °C for 5 min, NaH (2.62 mmol, 2 eq) was added and reacted at 25 °C for 0.5 h, halogenated alkane (2.62 mmol, 2 eq) was added dropwise at 0 °C, followed by a reaction at 25 °C for 3 h TLC (DCM/MeOH: 50/1) to monitor the completion of the reaction. H_2_O (20 mL) was added to quench the reaction, extracted with EA (10 mL × 3), washed with saturated NaCl (10 mL), dried over anhydrous Na_2_SO_4_ filtrate, the filtrate was dried and purified by column chromatography to obtain compound **38m**–**t**.

methyl 9-methyl-2,3,4,9-tetrahydro-1*H*-carbazole-6-carboxylate (**38m**).

White solid; yield: 98.6%; ^1^H NMR (300 MHz, Chloroform-*d*) *δ* 8.26 (d, *J* = 1.4 Hz, 1 H), 7.89 (dd, *J* = 8.6, 1.6 Hz, 1 H), 7.26 (d, *J* = 8.6 Hz, 1 H), 3.95 (s, 3 H), 3.65 (s, 3 H), 2.80–2.70 (m, 4 H), 2.04–1.83 (m, 4 H); MS (ESI): 244.1.

methyl 9-ethyl-2,3,4,9-tetrahydro-1*H*-carbazole-6-carboxylate (**38n**).

White solid; yield: 13.4%; ^1^H NMR (300 MHz, DMSO-*d*6) *δ* 8.09 (s, 1 H), 7.73 (d, *J* = 8.5 Hz, 1 H), 7.49 (d, *J* = 8.5 Hz, 1 H), 4.16 (q, *J* = 7.0 Hz, 2 H), 3.86 (s, 3 H), 2.83–2.65 (m, 4 H), 2.03–1.76 (m, 4 H), 1.26 (t, *J* = 6.8 Hz, 3 H); MS (ESI): 258.1.

methyl 9-propyl-2,3,4,9-tetrahydro-1*H*-carbazole-6-carboxylate (**38o**).

White solid; yield: 94.4%; MS (ESI): 272.2.

Methyl 9-isopropyl-2,3,4,9-tetrahydro-1*H*-carbazole-6-carboxylate (**38p**).

White solid; yield: 18.0%; ^1^H NMR (400 MHz, DMSO-*d*6) *δ* 8.04 (d, *J* = 1.5 Hz, 1 H), 7.67 (dd, *J* = 8.7, 1.7 Hz, 1 H), 7.58 (d, *J* = 8.7 Hz, 1 H), 4.76–4.59 (m, 1 H), 3.84 (s, 2 H), 2.77 (t, *J* = 5.8 Hz, 2 H), 2.66 (t, *J* = 5.8 Hz, 2 H), 1.92–1.83 (m, 2 H), 1.83–1.73 (m, 2 H), 1.51 (d, *J* = 7.0 Hz, 6 H); MS (ESI): 272.2.

Methyl 9-isobutyl-2,3,4,9-tetrahydro-1*H*-carbazole-6-carboxylate (**38q**).

White solid; yield: 32.1%; ^1^H NMR (400 MHz, DMSO-*d*6) *δ* 8.06 (d, *J* = 1.4 Hz, 1 H), 7.69 (dd, *J* = 8.6, 1.7 Hz, 1 H), 7.46 (d, *J* = 8.6 Hz, 1 H), 3.89 (d, *J* = 7.6 Hz, 2 H), 3.83 (s, 3 H), 2.76–2.66 (m, 4 H), 2.13–2.01 (m, 1 H), 1.92–1.74 (m, 4 H), 0.85 (d, *J* = 6.7 Hz, 6 H); MS (ESI): 286.2.

Methyl 9-(cyclopropylmethyl)-2,3,4,9-tetrahydro-1*H*-carbazole-6-carboxylate (**38r**).

White solid; yield: 60.7%; MS (ESI): 284.2.

Methyl 9-(cyclobutylmethyl)-2,3,4,9-tetrahydro-1*H*-carbazole-6-carboxylate (**38s**).

White solid; yield: 7.71%; ^1^H NMR (300 MHz, DMSO-*d*6) *δ* 8.05 (d, *J* = 1.7 Hz, 1 H), 7.69 (dd, *J* = 8.6, 1.7 Hz, 1 H), 7.51 (d, *J* = 8.6 Hz, 1 H), 4.12 (d, *J* = 7.1 Hz, 2 H), 3.83 (s, 3 H), 2.79–2.66 (m, 5 H), 1.93–1.71 (m, 10 H); MS (ESI): 298.2.

methyl 9-(oxetan-3-ylmethyl)-2,3,4,9-tetrahydro-1*H*-carbazole-6-carboxylate (**38t**).

White solid; yield: 5.67%; ^1^H NMR (300 MHz, DMSO-*d*6) *δ* 12.38 (s, 1 H), 8.06 (d, *J* = 1.2 Hz, 1 H), 7.71 (dd, *J* = 8.6, 1.5 Hz, 1 H), 7.43 (d, *J* = 8.6 Hz, 1 H), 3.65 (s, 3 H), 2.86–2.62 (m, 4 H), 2.03–1.73 (m, 4 H); MS (ESI): 230.1.

#### 3.1.9. General Procedure for the Synthesis of Compounds **39m**–**t**

9-methyl-2,3,4,9-tetrahydro-1*H*-carbazole-6-carboxylic acid (**39m**).

White solid; yield: 91.9%; ^1^H NMR (300 MHz, DMSO-*d*6) *δ* 12.38 (s, 1 H), 8.06 (d, *J* = 1.2 Hz, 1 H), 7.71 (dd, *J* = 8.6, 1.5 Hz, 1 H), 7.43 (d, *J* = 8.6 Hz, 1 H), 3.65 (s, 3 H), 2.86–2.62 (m, 4 H), 2.03–1.73 (m, 4 H); MS (ESI): 230.1.

9-ethyl-2,3,4,9-tetrahydro-1*H*-carbazole-6-carboxylic acid (**39n**).

White solid; yield: 79.2%; ^1^H NMR (400 MHz, DMSO-*d*6) *δ* 12.34 (s, 1 H), 8.04 (d, *J* = 1.2 Hz, 1 H), 7.69 (dd, *J* = 8.6, 1.6 Hz, 1 H), 7.43 (d, *J* = 8.6 Hz, 1 H), 4.13 (q, *J* = 7.1 Hz, 2 H), 2.73 (t, *J* = 5.6 Hz, 2 H), 2.67 (t, *J* = 5.5 Hz, 2 H), 1.93–1.75 (m, 4 H), 1.23 (t, *J* = 7.1 Hz, 3 H); MS (ESI): 244.1.

9-propyl-2,3,4,9-tetrahydro-1*H*-carbazole-6-carboxylic acid (**39o**).

White solid; yield: 85.1%; ^1^H NMR (300 MHz, DMSO-*d*6) *δ* 12.39 (s, 1 H), 8.07 (d, *J* = 1.6 Hz, 1 H), 7.71 (dd, *J* = 8.6, 1.4 Hz, 1 H), 7.46 (d, *J* = 8.6 Hz, 1 H), 4.06 (t, *J* = 7.2 Hz, 2 H), 2.81–2.63 (m, 4 H), 1.96–1.76 (m, 4 H), 1.75–1.60(m, 2 H), 0.88 (t, *J* = 7.4 Hz, 3 H); MS (ESI): 258.1.

9-isobutyl -2,3,4,9-tetrahydro-1*H*-carbazole-6-carboxylic acid (**39p**).

White solid; yield: 62.1%; ^1^H NMR (300 MHz, DMSO-*d*6) *δ* 8.09 (d, *J* = 1.6 Hz, 1 H), 7.72 (dd, *J* = 8.7, 1.4 Hz, 1 H), 7.62 (d, *J* = 8.7 Hz, 1 H), 4.81–4.69 (m, 1 H), 2.83 (t, *J* = 5.0 Hz, 1 H), 2.73 (t, *J* = 5.0 Hz, 2 H), 2.02–1.90 (m, 2 H), 1.90–1.79 (m, 2 H), 1.58 (d, *J* = 6.9 Hz, 6 H); MS (ESI): 258.1.

9-isobutyl-2,3,4,9-tetrahydro-1*H*-carbazole-6-carboxylic acid (**39q**).

White solid; yield: 95.3%; ^1^H NMR (300 MHz, DMSO-*d*6) *δ* 8.06 (d, *J* = 1.5 Hz, 1 H), 7.70 (dd, *J* = 8.6, 1.7 Hz, 1 H), 7.45 (d, *J* = 8.7 Hz, 1 H), 3.91 (d, *J* = 7.5 Hz, 2 H), 2.79–2.69 (m, 4 H), 2.15–2.00(m, 1 H), 1.95–1.76 (m, 4 H), 0.88 (d, *J* = 6.6 Hz, 6 H); MS (ESI): 272.2.

9-(cyclopropylmethyl)-2,3,4,9-tetrahydro-1*H*-carbazole-6-carboxylic acid (**39r**).

White solid; yield: 87.7%; ^1^H NMR (300 MHz, DMSO-*d*6) *δ* 8.06 (d, *J* = 1.5 Hz, 1 H), 7.70 (dd, *J* = 8.6, 1.4 Hz, 1 H), 7.49 (d, *J* = 8.6 Hz, 1 H), 4.02 (d, *J* = 6.7 Hz, 2 H), 2.84–2.64 (m, 4 H), 1.97–1.75 (m, 4 H), 1.24–1.08 (m, 1 H), 0.54–0.43 (m, 2 H), 0.43–0.33 (m, 2 H); MS (ESI): 270.1.

9-(cyclobutylmethyl)-2,3,4,9-tetrahydro-1*H*-carbazole-6-carboxylic acid (**39s**).

White solid; yield: 65.4%; ^1^H NMR (300 MHz, DMSO-*d*6) *δ* 8.05 (d, J = 1.6 Hz, 1 H), 7.70 (dd, *J* = 8.6, 1.7 Hz, 1 H), 7.50 (d, *J* = 8.6 Hz, 1 H), 4.14 (d, *J* = 7.0 Hz, 2 H), 2.80–2.60 (m, 5 H), 2.00–1.69 (m, 10 H); MS (ESI): 284.2.

9-(oxetan-3-ylmethyl)-2,3,4,9-tetrahydro-1*H*-carbazole-6-carboxylic acid (**39t**).

White solid; yield: 59.4%; MS (ESI): 286.1.

#### 3.1.10. General Procedure for the Synthesis of Target Products **1**–**26**

A mixture of **34a**–**f** or **36a**–**l** or **39m**–**t** (0.30 mmol, 1 eq), **29** (0.30 mmol, 1 eq), HATU (0.30 mmol, 1 eq), DIPEA (0.75 mmol, 2.5 eq) in DCM (2 mL) was stirred under an atmosphere of N_2_ for 3 h. The reaction mixture was concentrated under reduced pressure and purify by column chromatography (DCM + 5% Ammonium hydroxide) to obtain the titled compounds **1**–**26**.

(*S*)-*N*-(3-(3,4-dihydroisoquinolin-2(1*H*)-yl)-2-hydroxypropyl)-9*H*-carbazole-3-carboxamide (**1**).

White solid; yield: 25.2%; HPLC Purity: 97.6%; ^1^H NMR (300 MHz, Methanol-*d*4) *δ* 8.63 (s, 1 H), 8.05 (d, *J* = 7.8 Hz, 1 H), 7.88 (d, *J* = 8.5 Hz, 1 H), 7.55–7.37 (m, 3 H), 7.22 (t, *J* = 7.4 Hz, 1 H), 7.16–7.06 (m, 3 H), 7.05–6.98 (m, 1 H), 4.25–4.12 (m, 1 H), 3.76 (s, 2 H), 3.68–3.51 (m, 2 H), 2.98–2.80 (m, 4 H), 2.79–2.62 (m, 2 H); ^13^C NMR (75 MHz, Methanol-*d*4) *δ* 170.06, 142.09, 140.65, 133.62, 128.29, 126.26, 126.12, 126.05, 125.51, 124.57, 124.07, 122.79, 122.68, 119.89, 119.44, 119.24, 110.81, 110.17, 67.12, 62.25, 56.05, 51.25, 45.07, 28.14; HRMS (ESI+): *m*/*z* calcd for C_25_H_26_N_3_O_2_ 400.2025 [M + H]+; found: 400.2014.

(*S*)-*N*-(3-(3,4-dihydroisoquinolin-2(1*H*)-yl)-2-hydroxypropyl)-6-methyl-9*H*-carbazole-3-carboxamide (**2**).

White solid; yield: 50.0%; HPLC Purity: 98.8%; ^1^H NMR (300 MHz, Methanol-*d*4) *δ* 8.61 (d, *J* = 1.6 Hz, 1 H), 7.90 (s, 1 H), 7.87–7.82 (m, 1 H), 7.41 (s, 1 H), 7.38 (s, 1 H), 7.31–7.25 (m, 1 H), 7.16–7.10 (m, 3 H), 7.10–7.04 (m, 1 H), 4.25–4.15 (m, 1 H), 3.80 (s, 2 H), 3.69–3.50 (m, 2 H), 3.00–2.87 (m, 4 H), 2.83–2.37(m,2 H), 2.54 (s, 3 H); ^13^ C NMR (75 MHz, Methanol-*d*4) *δ* 170.05, 142.37, 138.92, 133.82, 133.58, 128.47, 128.23, 127.29, 126.21, 126.10, 125.48, 124.33, 123.90, 123.03, 122.54, 119.61, 119.41, 110.42, 109.90, 67.11, 56.07, 51.30, 44.94, 28.16, 20.15; HRMS (ESI+): *m*/*z* calcd for C_26_H_28_N_3_O_2_ 414.2182 [M + H]+; found: 414.2170.

(*S*)-*N*-(3-(3,4-dihydroisoquinolin-2(1*H*)-yl)-2-hydroxypropyl)-6-fluoro-9*H*-carbazole-3-carboxamide (**3**).

White solid; yield: 47.5%; HPLC Purity: 97.7%; ^1^H NMR (300 MHz, Methanol-*d*4) *δ* 8.60 (d, *J* = 1.4 Hz, 1 H), 7.93–7.87 (m, 1 H), 7.81–7.74 (m, 1 H), 7.51–7.46 (m, 1 H), 7.45–7.41 (m, 1 H), 7.27-.7.19 (m, 1 H), 7.17–7.09 (m, 3 H), 7.09–7.03 (m, 1 H), 4.26–4.14 (m, 1 H), 3.79 (s, 2 H), 3.69–3.52 (m, 2 H), 2.99–2.83 (m, 4 H), 2.82–2.60 (m, 2 H); ^13^C NMR (75 MHz, Methanol-*d*4) *δ* 169.73, 143.05, 137.02, 134.14, 133.72, 128.22, 126.18, 126.02, 125.42, 125.09, 124.28, 119.78, 113.72, 113.38, 111.58, 111.46, 110.31,105.40, 105.09, 67.14, 62.43, 56.21, 51.39, 45.06, 28.36; HRMS (ESI + ): *m*/*z* calcd for C_25_H_25_FN_3_O_2_ 418.1931 [M + H]+; found: 418.1919.

(*S*)-*N*-(3-(3,4-dihydroisoquinolin-2(1*H*)-yl)-2-hydroxypropyl)-6-methoxy-9*H*-carbazole-3-carboxamide (**4**).

White solid; yield: 60.8%; HPLC Purity: 97.4%; ^1^H NMR (300 MHz, Methanol-*d*4) *δ* 8.62 (s, 1 H), 7.84 (d, *J* = 8.6 Hz, 1 H), 7.64 (d, *J* = 2.1 Hz, 1 H), 7.39 (t, *J* = 8.3 Hz, 2 H), 7.16–7.06 (m, 3 H), 7.04–6.98 (m, 1 H), 4.23–4.13 (m, 1 H), 3.91 (s, 3 H), 3.75 (s, 2 H), 3.69–3.50 (m, 2 H), 2.96–2.82 (m, 4 H), 2.79–2.64 (m, 2 H); ^13^C NMR (75 MHz, Methanol-*d*4) *δ* 170.09, 153.90, 142.70, 135.52, 133.92, 133.62, 128.25, 126.24, 126.08, 125.47, 124.40, 123.61, 123.27, 119.63, 115.30, 111.56, 110.21, 102.72, 67.23, 62.13, 56.03, 55.22, 51.23, 44.96, 28.13; HRMS (ESI+): *m*/*z* calcd for C_26_H_28_N_3_O_3_ 430.2131 [M + H]+; found: 430.2118.

(*S*)-6-chloro-*N*-(3-(3,4-dihydroisoquinolin-2(1*H*)-yl)-2-hydroxypropyl)-9*H*-carbazole-3-carboxamide (**5**).

White solid; yield: 66.7%; HPLC Purity: 97.7%; ^1^H NMR (400 MHz, Methanol-*d*4) *δ* 8.59 (d, *J* = 1.4 Hz, 1 H), 8.07 (d, *J* = 2.0 Hz, 1 H), 7.91–7.86 (m, 1 H), 7.50–7.39 (m, 3 H), 7.15–7.09 (m, 3 H), 7.09–7.02 (m, 1 H), 4.23–4.15 (m, 1 H), 3.79 (s, 2 H), 3.67–3.49 (m, 2 H), 2.97–2.87 (m, 4 H), 2.77–2.66 (m, 2 H); ^13^C NMR (75 MHz, DMSO-*d*6) *δ* 167.37, 142.32, 139.18, 134.44, 128.87, 126.85, 126.47, 126.31, 126.19, 125.97, 125.86, 124.34, 123.91, 121.54, 120.92, 120.33, 113.28, 111.06, 67.38, 62.88, 56.43, 51.61, 45.47, 29.00; HRMS (ESI+): *m*/*z* calcd for C_25_H_25_ClN_3_O_2_ 434.1635 [M + H]+; found: 434.1627.

(*S*)-*N*-(3-(3,4-dihydroisoquinolin-2(1*H*)-yl)-2-hydroxypropyl)-9-methyl-9*H*-carbazole-3-carboxamide (**6**).

White solid; yield: 45.4%; HPLC Purity: 99.6%; ^1^H NMR (300 MHz, Methanol-*d*4) *δ* 8.57 (d, *J* = 1.8 Hz, 1 H), 8.00 (d, *J* = 7.7 Hz, 1 H), 7.94–7.87 (m, 1 H), 7.57–7.40 (m, 2 H), 7.32 (d, *J* = 8.6 Hz, 1 H), 7.27–7.18 (m, 1 H), 7.14–7.03 (m, 3 H), 7.04–6.92 (m, 1 H), 4.24–4.10 (m, 1 H), 3.75 (s, 3 H), 3.71 (s, 2 H), 3.67–3.52 (m, 2 H), 2.90–2.80 (m, 4 H), 2.71 (m, 2 H); ^13^C NMR (75 MHz, Methanol-*d*4) *δ* 169.66, 142.70, 141.56, 134.14, 133.72, 128.24, 126.20, 126.07, 125.98, 125.48, 125.41, 124.71, 124.26, 122.51, 122.23, 119.87, 119.32, 108.65, 107.89, 79.04, 67.13, 62.46, 56.18, 51.34, 45.12, 37.50, 36.44, 27.95; HRMS (ESI+): *m*/*z* calcd for C_26_H_28_N_3_O_2_ 414.2182 [M + H]+; found: 414.2167.

(*S*)-*N*-(3-(3,4-dihydroisoquinolin-2(1*H*)-yl)-2-hydroxypropyl)-2,3,4,9-tetrahydro-1*H*-carbazole-6-carboxamide (**7**).

White solid; yield: 48.1%; HPLC Purity: 99.8%; ^1^H NMR (300 MHz, Methanol-*d*4) *δ* 7.96 (s, 1 H), 7.52 (d, *J* = 8.5 Hz, 1 H), 7.22 (d, *J* = 8.5 Hz, 1 H), 7.18–7.06 (m, 3 H), 7.07–7.00 (m, 1 H), 4.21–4.08 (m,1 H), 3.76 (s, 2 H), 3.64–3.48 (m, 2 H), 2.99–2.82 (m,4 H), 2.80–2.62 (m, 6 H), 2.00–1.82 (m, 4 H); ^13^C NMR (75 MHz, Methanol-*d*4) *δ* 170.94, 138.13, 136.21, 133.80, 133.61, 128.30, 127.39, 126.30, 126.15, 125.55, 123.62, 119.34, 116.96, 109.96, 109.84, 67.14, 62.16, 56.00, 51.18, 44.97, 28.04, 23.01, 22.88, 22.62, 20.41; HRMS (ESI+): *m*/*z* calcd for C^25^H^30^N^3^O^2^ 404.2338 [M + H]+; found: 404.2326.

(*S*)-*N*-(3-(3,4-dihydroisoquinolin-2(1*H*)-yl)-2-hydroxypropyl)-1,2,3,4-tetrahydrocyclopenta[b]indole-7-carboxamide (**8**).

White solid; yield: 47.6%; HPLC Purity: 98.7%; ^1^H NMR (400 MHz, Methanol-*d*4) *δ* 7.91 (d, *J* = 1.5 Hz, 1 H), 7.51–7.45 (m, 1 H), 7.26 (d, *J* = 8.6 Hz, 1 H), 7.16–7.08 (m, 3 H), 7.08–6.98 (m, 1 H), 4.21–4.10 (m, 1 H), 3.76 (s, 2 H), 3.60–3.47 (m, 2 H), 2.96–2.90 (m,2 H), 2.90–2.85 (m, 4 H), 2.84–2.79 (m, 2 H), 2.76–2.64 (m, 2 H), 2.63–2.50 (m, 2 H); ^13^C NMR (75 MHz, Methanol-*d*4) *δ* 170.95, 146.13, 143.32, 133.70, 133.56, 128.33, 126.32, 126.20, 125.59, 124.11, 124.04, 119.24, 118.78, 117.65, 111.02, 67.11, 62.00, 55.90, 51.09, 44.91, 28.25, 27.94, 25.10, 23.73; HRMS (ESI+): *m*/*z* calcd for C_24_H_28_N_3_O_2_ 390.2182 [M + H]+; found: 390.2172.

(*S*)-*N*-(3-(3,4-dihydroisoquinolin-2(1*H*)-yl)-2-hydroxypropyl)-5,6,7,8,9,10-hexahydrocyclohepta[*b*]indole-2-carboxamide (**9**).

White solid; yield: 50.1%; HPLC Purity: 97.5%; ^1^H NMR (300 MHz, Methanol-*d*4) *δ* 8.00 (d, *J* = 1.7 Hz, 1 H), 7.50–7.45 (m, 1 H), 7.20 (d, *J* = 8.5 Hz, 1 H), 7.15–7.09 (m, 3 H), 7.07–7.01 (m, 1 H), 4.23–4.10 (m, 1 H), 3.77 (s, 2 H), 3.66–3.46 (m, 2 H), 2.98–2.80 (m, 8 H), 2.79–2.59 (m, 2 H), 2.01–1.89 (m, 2 H), 1.88–1.70 (m, 4 H); ^13^C NMR (75 MHz, Methanol-*d*4) *δ* 171.02, 136.68, 133.44, 133.44, 128.68, 128.30, 126.28, 126.25, 125.61, 123.64, 118.89, 116.97, 113.66, 67.08, 61.89, 55.88, 51.16, 44.80, 31.68, 28.62, 27.87, 24.22; HRMS (ESI+): *m*/*z* calcd for C_26_H_32_N_3_O_2_ 418.2495 [M + H]+; found: 418.2483.

(*S*)-*N*-(3-(3,4-dihydroisoquinolin-2(1*H*)-yl)-2-hydroxypropyl)-6,7,8,9,10,11-hexahydro-5*H*-cycloocta[b]indole-2-carboxamide (**10**).

White solid; yield: 51.3%; HPLC Purity: 97.0%; ^1^H NMR (300 MHz, Methanol-*d*4) *δ* 8.03 (s, 1 H), 7.54–7.47 (m, 1 H), 7.23 (d, *J* = 8.5 Hz, 1 H), 7.20–7.10 (m, 3 H), 7.10–7.03 (m, 1 H), 4.24–4.12 (m, 1 H), 3.79 (s, 2 H), 3.66–3.46 (m, 2 H), 3.01–2.84 (m, 8 H), 2.80–2.65 (m, 2 H), 1.87–1.70 (m, 4 H), 1.58–1.41 (m, 4 H); ^13^C NMR (75 MHz, Methanol-*d*4) *δ* 170.95, 137.78, 137.52, 133.96, 133.67, 128.26, 127.95, 126.25, 126.08, 125.48, 123.70, 118.95, 116.99, 111.69, 109.82, 67.28, 62.18, 56.10, 51.28, 44.89, 29.80, 29.27, 28.17, 25.62, 25.59, 24.96, 21.62; HRMS (ESI+): *m*/*z* calcd for C_27_H_34_N_3_O_2_ 432.2651 [M + H]+; found: 432.2639.

*N*-((*S*)-3-(3,4-dihydroisoquinolin-2(1*H*)-yl)-2-hydroxypropyl)-3-methyl-2,3,4,9-tetrahydro-1H-carbazole-6-carboxamide (**11**).

White solid; yield: 45.9%; HPLC Purity: 97.7%; ^1^H NMR (300 MHz, Methanol-*d*4) *δ* 7.96 (s, 1 H), 7.55–7.49 (m, 1 H), 7.22 (d, *J* = 8.5 Hz, 1 H), 7.19–7.10 (m, 3 H), 7.09–7.03 (m, 1 H), 4.27–4.10 (m, 1 H), 3.80 (s, 2 H), 3.66–3.48 (m, 2 H), 2.99–2.87 (m, 4 H), 2.86–2.78 (m, 3 H), 2.77–2.65 (m, 2 H), 2.35–2.20 (m, 1 H), 2.08–1.90 (m, 2 H), 1.68–1.51 (m, 1 H), 1.18 (d, *J* = 6.5 Hz, 3 H); ^13^C NMR (75 MHz, Methanol-*d*4) *δ* 170.90, 138.46, 135.87, 133.86, 133.62, 128.27, 127.29, 126.26, 126.12, 125.51, 123.68, 119.29, 116.93, 109.88, 109.74, 62.19, 56.05, 51.23, 44.93, 31.14, 29.53, 28.91, 28.11, 22.25, 20.75; HRMS (ESI+): *m*/*z* calcd for C_26_H_32_N_3_O_2_ 418.2495 [M + H]+; found: 418.2483.

*N*-((*S*)-3-(3,4-dihydroisoquinolin-2(1*H*)-yl)-2-hydroxypropyl)-3-ethyl-2,3,4,9-tetrahydro-1*H*-carbazole-6-carboxamide (**12**).

White solid; yield: 41.1%; HPLC Purity: 99.0%; ^1^HNMR (300 MHz, Methanol-*d*4) *δ* 7.97 (s, 1 H), 7.52 (d, *J* = 8.5 Hz, 1 H), 7.22 (d, *J* = 8.4 Hz, 1 H), 7.20–7.10 (m, 3 H), 7.10–7.02 (m, 1 H), 4.17 (m, 1 H), 3.79 (s, 2 H), 3.64–3.48 (m, 2 H), 2.99–2.83 (m, 5 H), 2.83–2.67 (m, 4 H), 2.33–2.19 (m, 1 H), 2.13–2.02 (m, 1 H), 1.80–1.64 (m 1 H), 1.55 (m, 3 H), 1.07 (t, *J* = 7.4 Hz, 3 H); ^13^C NMR (75 MHz, Methanol-*d*4) *δ* 170.93, 138.47, 136.16, 133.80, 133.60, 128.28, 127.37, 126.28, 126.15, 125.54, 123.65, 119.30, 116.93, 109.93, 109.68, 62.11, 56.01, 51.20, 36.46, 28.99, 28.65, 28.05, 26.61, 22.32, 10.85; HRMS (ESI+): *m*/*z* calcd for C_27_H_34_N_3_O_2_ 432.2651 [M + H]+; found: 432.2639.

(*S*)-*N*-(3-(3,4-dihydroisoquinolin-2(1*H*)-yl)-2-hydroxypropyl)-9-methyl-2,3,4,9-tetrahydro-1*H*-carbazole-6-carboxamide (**13**).

White solid; yield: 57.6%; HPLC Purity: 99.1%; ^1^H NMR (300 MHz, Methanol-*d*4) *δ* 7.98 (s, 1 H), 7.63–7.57 (m, 1 H), 7.25 (d, *J* = 8.6 Hz, 1 H), 7.19–7.08 (m, 3 H), 7.08–7.02 (m, 1 H), 4.23–4.11 (m, 1 H), 3.78 (s, 2 H), 3.64 (s, 3 H), 3.62–3.50 (m, 2 H), 2.98–2.84 (m, 4 H), 2.80–2.65 (m, 6 H), 2.04–1.83 (m, 4 H); ^13^C NMR (75 MHz, Methanol-*d*4) *δ* 170.90, 138.75, 133.57, 133.53, 128.34, 126.83, 126.34, 126.24, 125.63, 123.59, 119.30, 117.08, 109.93, 108.03, 67.04, 62.01, 55.89, 51.13, 44.93, 28.10, 27.88, 22.79, 21.46, 20.47; HRMS (ESI+): *m*/*z* calcd for C_26_H_32_N_3_O_2_ 418.2495 [M + H]+; found: 418.2484.

(*S*)-*N*-(3-(3,4-dihydroisoquinolin-2(1*H*)-yl)-2-hydroxypropyl)-9-ethyl-2,3,4,9-tetrahydro-1*H*-carbazole-6-carboxamide (**14**).

White solid; yield: 46.7%; HPLC Purity: 97.4%; ^1^H NMR (400 MHz, Methanol-*d*4) *δ* 7.94 (d, *J* = 1.6 Hz, 1 H), 7.56–7.52 (m, 1 H), 7.19 (d, *J* = 8.6 Hz, 1 H), 7.12–7.02 (m, 3 H), 7.00–6.97 (m, 1 H), 4.14–4.08 (m, 1 H), 4.05 (q, *J* = 7.3 Hz, 2 H), 3.68 (s, 2 H), 3.58–3.46 (m, 2 H), 2.88–2.82 (m, 2 H), 2.81–2.78 (m, 2 H), 2.71–2.60 (m, 6 H), 1.95–1.87 (m, 2 H), 1.86–1.78 (m, 2 H), 1.24 (t, *J* = 7.2 Hz, 3 H); ^13^C NMR (75 MHz, Methanol-*d*4) *δ* 170.55, 137.62, 134.18, 133.76, 128.21, 126.21, 125.39, 123.84, 119.35, 110.07, 107.91, 67.19, 62.46, 56.21, 51.34, 45.01, 37.50, 37.00, 28.37, 22.92, 21.46, 20.54, 14.33; HRMS (ESI+): *m*/*z* calcd for C_27_H_34_N_3_O_2_ 432.2651[M + H]+; found: 432.2638.

*N*-((2*R*)-2-hydroxy-3-(1,2,3,4-tetrahydronaphthalen-2-yl)propyl)-9-propyl-2,3,4,9-tetrahydro-1*H*-carbazole-6-carboxamide (**15**).

White solid; yield: 50.2%; HPLC Purity: 97.0%; ^1^H NMR (300 MHz, Methanol-*d*4) *δ* 7.94 (d, *J* = 1.7 Hz, 1 H), 7.55–7.49 (m, 1 H), 7.14 (d, *J* = 8.6 Hz, 1 H), 7.11–6.99 (m, 3 H), 6.99–6.91 (m, 1 H), 4.14–4.04 (m, 1 H), 3.92 (t, *J* = 7.2 Hz, 2 H), 3.65 (s, 2 H), 3.59–3.44 (m, 2 H), 2.88–2.80 (m, 2 H), 2.77–2.71 (m, 2 H), 2.69–2.55 (m, 6 H), 1.94–1.75 (m, 4 H), 1.74–1.57 (m, 2 H), 1.1–1.01 (m, 2 H), 0.85 (t, *J* = 7.4 Hz, 3 H); ^13^C NMR (75 MHz, Methanol-*d*4) *δ* 170.55, 137.62, 134.18, 133.76, 128.21, 126.21, 125.39, 123.84, 119.35, 110.07, 107.91, 67.19, 62.46, 56.21, 51.34, 45.01, 37.50, 37.00, 28.37, 22.92, 21.46, 20.54, 14.33; HRMS (ESI+): *m*/*z* calcd for C_29_H_37_N_2_O_2_ 446.2855 [M + H]+; found: 446.2796.

(*S*)-*N*-(3-(3,4-dihydroisoquinolin-2(1*H*)-yl)-2-hydroxypropyl)-9-isopropyl-2,3,4,9-tetrahydro-1*H*-carbazole-6-carboxamide (**16**).

White solid; yield: 40.5%; HPLC Purity: 99.2%; ^1^H NMR (300 MHz, DMSO-*d*4) *δ* 8.41 (t, *J* = 5.1 Hz, 1 H), 7.97 (s, 1 H), 7.59–7.40 (m, 2 H), 7.17–7.09 (m, 3 H), 7.09–7.02 (m, 1 H), 4.99 (s, 1 H), 4.73–4.60 (m, 1 H), 4.04–3.92 (m, 1 H), 3.69 (s, 2 H), 3.33–3.24 (m, 2 H), 2.87–2.74 (m, 6 H), 2.68–2.56 (m, 4 H), 1.95–1.72 (m, 4 H), 1.52 (d, *J* = 6.9 Hz, 6 H); ^13^C NMR (101 MHz, DMSO-*d*4) *δ* 167.98, 136.78, 136.38, 135.29, 134.52, 128.86, 127.45, 126.84, 126.38, 125.91, 124.76, 119.93, 117.65, 110.07, 67.46, 56.53, 51.65, 46.71, 45.56, 38.70, 29.10, 23.47, 23.09, 22.94, 21.92, 21.11; HRMS (ESI+): *m*/*z* calcd for C_28_H_36_N_3_O_2_ 446.2808 [M + H]+; found: 446.2798.

(*S*)-*N*-(3-(3,4-dihydroisoquinolin-2(1*H*)-yl)-2-hydroxypropyl)-9-isobutyl-2,3,4,9-tetrahydro-1*H*-carbazole-6-carboxamide (**17**).

White solid; yield: 29.9%; HPLC Purity: 98.7%; ^1^H NMR (300 MHz, Methanol-*d*4) *δ* 8.01 (s, 1 H), 7.58 (d, *J* = 8.5 Hz, 1 H), 7.21 (d, *J* = 8.6 Hz, 1 H), 7.17–7.08 (m, 3 H), 7.08–7.00 (m, 1 H), 4.23–4.11 (m, 1 H), 3.85 (d, *J* = 7.4 Hz, 2 H), 3.77 (s, 2 H), 3.59 (d, *J* = 5.6 Hz, 1 H), 3.02–2.89 (m, 4 H), 2.79–2.60 (m, 6 H), 2.25–2.06 (m, 1 H), 2.01–1.82 (m, 4 H), 1.42–1.31 (m, 2 H), 0.93 (d, *J* = 6.5 Hz, 6 H); ^13^C NMR (75 MHz, Methanol-*d*4) *δ* 170.49, 138.49, 137.13, 134.10, 133.74, 126.95, 126.25, 125.99, 125.44, 123.80, 119.29, 110.00, 108.63, 67.08, 62.47, 56.19, 51.34, 45.06, 37.51, 29.40, 28.34, 23.02, 22.86, 22.09, 20.57, 19.11, 16.79; HRMS (ESI+): *m*/*z* calcd for C_29_H_38_N_3_O_2_ 460.2964 [M + H]+; found: 460.2953.

(*S*)-9-(cyclopropylmethyl)-*N*-(3-(3,4-dihydroisoquinolin-2(1*H*)-yl)-2-hydroxypropyl)-2,3,4,9-tetrahydro-1*H*-carbazole-6-carboxamide (**18**).

White solid; yield: 48.8%; HPLC Purity: 99.8%; ^1^H NMR (300 MHz, Methanol-*d*4) *δ* 7.96 (d, *J* = 1.7 Hz, 1 H), 7.56–7.51 (m, 1 H), 7.20 (d, *J* = 8.6 Hz, 1 H), 7.15–7.03 (m, 3 H), 7.01–6.95 (m, 1 H), 4.17–4.06 (m, 1 H), 3.91 (d, J = 6.4 Hz, 2 H), 3.69 (s, 2 H), 3.61–3.46 (m, 2 H), 2.91–2.84 (m, 2 H), 2.83.-2.76 (m, 2 H), 2.74–2.58 (m, 6 H), 1.97–1.89 (m, 2 H), 1.88–1.78 (m, 2 H), 1.18–1.04 (m, 1 H), 0.53–0.44 (m, 2 H), 0.36–0.24 (m, 2 H); ^13^C NMR (75 MHz, Methanol-*d*4) *δ* 170.46, 138.36, 136.70, 134.18, 133.77, 128.23, 127.00, 126.22, 125.95, 125.40, 123.83, 117.13, 110.10, 108.38, 67.13, 62.50, 56.21, 51.34, 46.25, 45.05, 37.50, 28.39, 22.84, 21.95, 20.55, 11.22, 2.88; HRMS (ESI+): *m*/*z* calcd for C_29_H_36_N_3_O_2_ 458.2808 [M + H]+; found: 458.2796.

(*S*)-9-(cyclobutylmethyl)-*N*-(3-(3,4-dihydroisoquinolin-2(1*H*)-yl)-2-hydroxypropyl)-2,3,4,9-tetrahydro-1*H*-carbazole-6-carboxamide (**19**).

White solid; yield: 96.2%; HPLC Purity: 99.4%; ^1^H NMR (300 MHz, Methanol-*d*4) *δ* 7.95 (d, *J* = 1.8 Hz, 1 H), 7.56–7.49 (m, 1 H), 7.22 (d, *J* = 8.7 Hz, 1 H), 7.16–7.05 (m, 3 H), 7.05–6.98 (m, 1 H), 4.19–4.09 (m, 1 H), 4.04 (d, *J* = 7.0 Hz, 2 H), 3.73 (s, 2 H), 3.60–3.49 (m, 2 H), 2.92–2.83 (m, 4 H), 2.78–2.64 (m, 6 H), 2.01–1.74 (m, 10 H); ^13^C NMR (75 MHz, Methanol-*d*4) *δ* 170.51, 138.50, 136.86, 134.19, 133.78, 128.23, 126.91, 126.23, 125.96, 125.41, 123.79, 119.25, 117.09, 110.00, 108.45, 67.12, 62.51, 56.23, 51.38, 45.05, 37.49, 36.61, 28.38, 25.90, 22.85, 22.05, 20.55, 17.78; HRMS (ESI+): *m*/*z* calcd for C_30_H_38_N_3_O_2_ 472.2964 [M + H]+; found: 472.2953.

(*S*)-*N*-(3-(3,4-dihydroisoquinolin-2(1*H*)-yl)-2-hydroxypropyl)-9-(oxetan-3-ylmethyl)-2,3,4,9-tetrahydro-1*H*-carbazole-6-carboxamide (**20**).

White solid; yield: 57.4%; HPLC Purity: 97.8%; ^1^H NMR (400 MHz, DMSO-*d*6) *δ* 7.96 (s, 1 H), 7.61–7.53 (m, 1 H), 7.33 (d, *J* = 8.6 Hz, 1 H), 7.17–7.07 (m, 3 H), 7.07–7.00 (m, 1 H), 4.74 (t, *J* = 7.0 Hz, 2 H), 4.52 (t, *J* = 6.1 Hz, 2 H), 4.42 (d, *J* = 7.4 Hz, 2 H), 4.20–4.08 (m, 1 H), 3.76 (s, 2 H), 3.60–3.53 (m, 2 H), 3.51–3.43(m, 1 H), 2.95–2.81 (m, 4 H), 2.81–2.73 (m, 2 H),2.73–2.61 (m, 4 H),2.02–1.80 (m, 4 H); ^13^C NMR (75 MHz, Methanol-*d*4) *δ* 170.27, 138.36, 136.68, 134.21, 133.81, 128.29, 127.1) 3, 126.28, 126.02, 125.47, 124.33, 119.75, 117.33, 110.67, 108.30, 74.91, 67.16, 62.54, 56.24, 51.38, 45.12, 44.65, 35.87, 28.44, 23.01, 22.79, 22.03, 20.55; HRMS (ESI+): *m*/*z* calcd for C_29_H_36_N_3_O_3_ 474.2757 [M + H]+; found: 474.2744.

(*S*)-*N*-(3-(3,4-dihydroisoquinolin-2(1*H*)-yl)-2-hydroxypropyl)-2-methyl-2,3,4,5-tetrahydro-1*H*-pyrido[4,3-*b*]indole-8-carboxamide (**21**).

White solid; yield: 41.1%; HPLC Purity: 96.9%; ^1^H NMR (300 MHz, Methanol-*d*4) *δ* 7.98 (s, 1 H), 7.61–7.54 (m, 1 H), 7.28 (d, *J* = 8.3 Hz, 1 H), 7.20–7.10 (m, 3 H), 7.10–7.02 (m, 1 H), 4.23–4.10 (m, 1 H), 3.78 (s, 2 H), 3.73 (s, 2 H), 3.67–3.47 (m, 2 H), 3.01–2.83 (m, 8 H), 2.79–2.62 (m, 2 H),2.59 (s, 3 H); ^13^C NMR (75 MHz, Methanol-*d*4) δ 170.44, 134.07, 133.72, 133.44, 128.27, 126.25, 126.10, 125.47, 125.38, 124.53, 119.95, 116.88, 110.23, 107.60, 67.17, 56.16, 51.34, 51.09, 45.01, 44.23, 28.31, 22.81; HRMS (ESI+): *m*/*z* calcd for C_25_H_31_N_4_O_2_ 419.2447 [M + H]+; found: 419.2449.

(*S*)-*N*-(3-(3,4-dihydroisoquinolin-2(1*H*)-yl)-2-hydroxypropyl)-2-ethyl-2,3,4,5-tetrahydro-1*H*-pyrido[4,3-*b*]indole-8-carboxamide (**22**).

White solid; yield: 30.8%; HPLC Purity: 97.0%; ^1^H NMR (300 MHz, Methanol-*d*4) *δ* 8.00 (s, 1 H), 7.61–7.54 (m, 1 H), 7.26 (d, *J* = 8.5 Hz, 1 H), 7.18–7.07 (m, 3 H), 7.06–6.94 (m, 1 H), 4.20–4.10 (m,1 H), 3.77–3.69 (m, 4 H), 3.65–3.47 (m, 2 H), 2.96–2.80 (m, 9 H), 2.79–2.63 (m, 4 H), 1.26 (t, *J* = 7.2 Hz, 3 H); ^13^C NMR (75 MHz, Methanol-*d*4) *δ* 170.42, 138.55, 134.20, 133.91, 133.77, 128.23, 126.22, 126.00, 125.55, 125.42, 124.43, 119.83, 116.84, 110.20, 107.79, 67.24, 62.37, 56.19, 51.36, 49.81, 48.69, 44.97, 36.46, 28.37, 22.87, 10.97; HRMS (ESI+): *m*/*z* calcd for C_26_H_33_N_4_O_2_ 433.2604 [M + H]+; found: 433.2596.

(*S*)-2-cyclopropyl-*N-*(3-(3,4-dihydroisoquinolin-2(1*H*)-yl)-2-hydroxypropyl)-2,3,4,5-tetrahydro-1*H*-pyrido[4,3-*b*]indole-8-carboxamide (**23**).

White solid; yield: 31.9%; HPLC Purity: 98.6%; ^1^H NMR (300 MHz, Methanol-*d*4) *δ* 7.99 (d, *J* = 1.7 Hz, 1 H), 7.5–7.52 (m, 1 H), 7.23 (d, *J* = 8.5 Hz, 1 H), 7.14–7.03 (m, 3 H),7.02–6.96(m, 1 H), 4.18–4.07 (m, 1 H), 3.84 (s, 2 H), 3.72–3.67 (m, 2 H), 3.63–3.46 (m, 2 H), 3.04 (t, *J* = 5.8 Hz, 2 H), 2.91–2.75 (m, 8 H), 2.74–2.57 (m, 2 H), 2.02–1.92 (m, 1 H), 0.66–0.49 (m, 4 H); ^13^C NMR (75 MHz, Methanol-*d*4) *δ* 170.43, 138.50, 134.20, 133.85, 133.77, 128.24, 126.23, 126.01, 125.55, 125.42, 124.41, 119.81, 116.89, 110.19, 108.01, 78.98, 67.25, 62.36, 56.19, 51.33, 50.47, 49.30, 44.97, 37.74, 36.47, 28.38, 22.83, 5.02; HRMS (ESI+): *m*/*z* calcd for C_28_H_35_N_4_O_2_ 445.2604 [M + H]+; found: 445.2597.

(*S*)-*N*-(3-(3,4-dihydroisoquinolin-2(1*H*)-yl)-2-hydroxypropyl)-2-propyl-2,3,4,5-tetrahydro-1*H*-pyrido[4,3-*b*]indole-8-carboxamide (**24**).

White solid; yield: 53.3%; HPLC Purity: 99.5%; ^1^H NMR (300 MHz, Methanol-*d*4) *δ* 7.96 (d, *J* = 1.7 Hz, 1 H), 7.57–7.51 (m, 1 H), 7.25 (d, *J* = 8.5 Hz, 1 H), 4.20–4.10 (m, 1 H), 3.79–3.71 (m, 4 H), 3.61–3.46 (m, 2 H), 2.94–2.81 (m, 9 H), 2.71–2.59 (m, 2 H), 1.79–1.63 (m, 2 H), 1.01 (t, *J* = 7.4 Hz, 3 H); ^13^C NMR (75 MHz, Methanol-*d*4) *δ* 170.50, 138.54, 134.19, 133.89, 133.77, 128.22, 126.20, 126.00, 125.53, 125.41, 124.43, 119.79, 110.14, 107.81, 67.25, 62.35, 59.81, 56.21, 51.36, 50.31, 49.19, 44.93, 28.35, 22.84, 19.75, 10.90; HRMS (ESI+): *m*/*z* calcd for C_27_H_35_N_4_O_2_ 447.2760 [M + H]+; found: 447.2749.

(*S*)-*N*-(3-(3,4-dihydroisoquinolin-2(1*H*)-yl)-2-hydroxypropyl)-2-isopropyl-2,3,4,5-tetrahydro-1*H*-pyrido[4,3-*b*]indole-8-carboxamide (**25**).

White solid; yield: 21.2%; HPLC Purity: 96.7%; ^1^H NMR (300 MHz, Methanol-*d*4) *δ* 8.01 (d, *J* = 1.7 Hz, 1 H), 7.60–7.53 (m, 1 H), 7.26 (d, *J* = 8.5 Hz, 1 H), 7.17–7.06 (m, 3 H), 7.04–6.98 (m, 1 H), 4.20–4.10 (m, 1 H), 3.82 (s, 2 H), 3.72 (s, 2 H), 3.66–3.46 (m, 2 H), 3.08–2.98 (m, 1 H), 2.97–2.86 (m, 6 H), 2.86–2.76 (m, 2 H), 2.75–2.59 (m, 2 H), 1.22 (d, *J* = 6.5 Hz, 6 H); ^13^C NMR (75 MHz, Methanol-*d*4) *δ* 170.42, 138.58, 134.19, 134.04, 133.77, 128.23, 126.23, 126.00, 125.70, 125.42, 124.39, 119.81, 116.84, 110.21, 108.12, 67.26, 62.33, 56.18, 54.31, 51.33, 45.91, 44.95, 44.44, 28.36, 23.34, 17.33; HRMS (ESI+): *m*/*z* calcd for C_27_H_35_N_4_O_2_ 447.2760 [M + H]+; found: 447.2751.

(*S*)-*N*-(3-(3,4-dihydroisoquinolin-2(1*H*)-yl)-2-hydroxypropyl)-2-isobutyl-2,3,4,5-tetrahydro-1*H*-pyrido[4,3-*b*]indole-8-carboxamide (**26**).

White solid; yield: 28.0%; HPLC Purity: 99.6%; ^1^HNMR (300 MHz, Methanol-*d*4) *δ* 7.97 (d, *J* = 1.7 Hz, 1 H), 7.57–7.51 (m, 1 H), 7.24 (d, *J* = 8.5 Hz, 1 H), 7.16–7.04 (m, 3 H), 7.04–6.90 (m, 1 H), 4.18–4.04 (m, 1 H), 3.70 (s, 2 H), 3.64 (s, 2 H), 3.60–3.46 (m, 2 H), 2.90–2.77 (m, 8 H), 2.72–2.59 (m, 2 H), 2.38 (d, *J* = 7.2 Hz, 2 H), 2.04–1.89 (m, 1 H), 0.98 (d, *J* = 6.6 Hz, 6 H); ^13^C NMR (75 MHz, Methanol-*d*4) *δ* 170.47, 138.49, 134.20, 134.13, 133.77, 128.23, 126.22, 125.99, 125.65, 125.41, 124.32, 119.71, 116.80, 110.15, 108.18, 67.25, 66.26, 56.20, 51.33, 50.68, 44.95, 36.45, 25.65, 22.89, 20.25; HRMS (ESI+): *m*/*z* calcd for C_28_H_37_N_4_O_2_ 461.2917 [M + H]+; found: 461.2911.

### 3.2. Biological Activity Assay

#### 3.2.1. PRMT5 Enzymatic Activity Inhibition Assays

The PRMT5 enzymatic activity assay was conducted by Chempartner Life science. The final concentrations of PRMT5 enzyme: 2.5 nm/L; ^3^H-SAM: 0.3 μM/L; histone peptide (H4R3S1ac): 0.3 μM/L. The protocol of radioactive methylation assay was as follows: (1) Prepare 1× assay buffer (modified Tris Buffer). (2) Transfer compounds to assay plate using Echo in 100% DMSO. DMSO’s final fraction is 1%. (3) Prepare enzyme and ^3^H-SAM Mix solution in 1× assay buffer. (4) Prepare substrate (peptide sequence Ac-SGRGKGGKGLGKGGAKRHRKVGG-K(Biotin)) solution in 1× assay buffer. (5) Transfer 15 μL of enzyme and ^3^H-SAM solution to assay plate. (6) Incubate at room temperature for 60 min. (7) Add 10 μL of substrate solution to each well to start the reaction, and for low control transfer 10 μL of 1× assay buffer. (8) Incubate at room temperature for 120 min. (9) Add cold SAM in 1× assay buffer to make the stop mix. (10) Stop reaction with addition of 5 µL per well of stop solution. (11) Transfer 25 µL of volume per well to Flash plate from assay plate. (12) Incubate for 1 h minimum at room temperature.

(13) Read plate on Micro beta. (14) Data process—get the data in Excel to obtain inhibition values using Equation (1).

Inhibitory rate% = (Max-Signal)/ (Max-Min) × 100(1)

Fit the data in XL-Fit to obtain IC_50_ values using Equation (2):

Y = Bottom + (Top-Bottom)/(1 + (IC_50_/X) × Hillslope) Y is% inhibition and X is compound concentration (2)

#### 3.2.2. Cell Lines, Antibodies and Agents

MV-4-11 and MDA-MB-468 cell line were derived from Cell Resources Center of Shanghai Academy of life sciences (Shanghai, China). AML-12 cell line was brough from Nanjing Lixing Biotechnology Co. Ltd. (Nanjing, China). MDA-MB-468 cell line was maintained in Dulbecco’s Modified Eagle Medium (DMEM, KeyGEN Bio) that contained 10% FBS (Biochanel, China), MV-4-11 cell line was maintained in Iscove’s Modified Dulbecco Medium containing; 10% FBS (Biochannel, China), AML-12 cell line was maintained in DMEM/F-12 (1:1) (KeyGEN, China; 1% ITS Liquid Media Supplement (Sigma, America), dexamethasone 40 ng/mL (Sigma, USA) and 10% FBS (Biochannel, China). All cells were grown in a humidified incubator containing 5% carbon dioxide at 37 ℃. All compounds were dispersed in dimethyl sulfoxide (Sigma), the concentration of the reserve solution was 10 mmol/L and the solution was stored in a refrigerator at −20 ℃. SDMA (# 13222S), β- Tubulin (#2146S) and horseradish peroxidase (HRP)-linked secondary antibodies anti-rabbit or anti-mouse IgG were purchased from Cell Signaling Technology (CST), and the anti-PRMT5 (ab109451) antibody was purchased from ABCAM.

#### 3.2.3. Anti-Proliferation Cells Assay of Compounds

The antiproliferative activities of compounds in vitro were measured by CellTiter-Glo (CTG) assay (Promega, America). In linear/log-phase growth, cultured MV-4-11 cells were split to with seeding density of 2 × 10^5^ cells/mL in 1–20 mL of media, depending on the yield required at the end of the growth period. At the conclusion of each treatment period, cells were harvested by centrifugation (4 min, 1000 rpm), and cell pellets were rinsed twice with PBS before being research further processing. Afterwards, cells were plated in 96-well plates with a density of 500 cells/well in 1 mL media. After six hours incubation, compounds treatment was performed. Half of the culture medium was moved and re-plated with fresh media with fresh compound after 6 days. On the 12th day, Cell Titer-Glo Luminescent Cell Viability assays (Promega, USA) were used to determine the viable cell number, and luminescence was recorded using the Envision Multilabel Plate Readers (PerkinElmer). The concentration dependence curves were determined at each time point using GraphPad Prism 8.0 software.

#### 3.2.4. Western Blot Assays

Compounds were administered at the expected concentration for the expected time in MV-4-11 cell lines. After being washed twice with PBS, cells were lysed with RIPA buffer, protease inhibitors (PMSF, MCE, New York, NY, USA), and phosphatase cocktails II (MCE, USA) for 1 h. The mixture was centrifuged at 12000 rpm for 15 min. The total protein content was quantified by BCA kit (Beyotime, Shanghai, China). Equivalent proteins were electrophoresed through 8~10% SDS-PAGE and then transferred on to polyvinylidene difluoride membranes (Millipore, Burlington, MA, USA). Subsequently, block the membrane for 1~2 h by 5% (*w*/*v*) skim milk (BD, Columbus, OH, USA). Proteins were detected using primary antibodies, followed by HRP-conjugated secondary antibodies, and visualization was performed using ECL (Vazyme Biotech, Beijing, China) as the HRP substrate. The target blots were detected with chemiluminescence system (Tanon, Shanghai, China). The densitometric analysis was performed with Image J software.

Tumor tissue was grinded by a cryo-grinder before being lysed with an RIPA buffer, protease inhibitors (PMSF, MCE), and phosphatase cocktails II (MCE). The mixture was centrifuged for 15 min (12,000 rpm). The following steps were the same as with cells.

#### 3.2.5. CETSA

The cellular thermal shift assay and the isothermal dose response were run as previously described. Briefly, MV-4-11 cells were collected and centrifugated (4 min, 1000 rpm) and washed twice with PBS. Then the cells were divided three equal parts with seeding density of 1 × 10^6^ cells/mL into 60 mm cell culture dish in three mL media. The cell suspension was incubated with DMSO and compounds for 18 h at 37 °C, 5% CO_2_, and 85% RH.

After incubation, cells were collected and washed twice with PBS. Then they divided the ten parts into transferred thin-walled PCR tubes (0.2 mL, Axygen, Union City, CA, USA) in 50μL PBS. Cells were subjected to 3 min of heat in 96-well thermal cycler (Eppendorf Mastercycler nexus GSX1, Düsseldorf, German) at temperatures of 37, 46, 49, 52, 55, 58, 61, 64, 67 and 70 °C. After heating, cells were left to cool for 3 min and then snap frozen in −80 °C ultra-low temperature freezer overnight. The next day, cells were subjected to 3 rapid freeze thaws with 15 s of vortexing after each thaw. Cell lysates were centrifuged at 12000 rpm for 10 min to separate the soluble fractions from precipitates, and the supernatant was transferred (∼40 μL) to 1.5 mL Eppendorf tube and 8 μL 6 × Loading Buffer, stored at −80 °C until analysis. Western blot step was the same as cells and tumor tissue.

#### 3.2.6. Pharmacokinetic Study in Mice

The pharmacokinetic properties were evaluated by PRECEDO. China. SPF ICR mice (18–20 g, 6–8 weeks, n = 3 per group) were used to carried out pharmacokinetics determinations. The compound was formulated into 0.4 mg/mL test solution with DMSO, PEG400 and 5% glucose injection and administered intravenously at a dose of 1 mg/kg. Colleting samples at different times after treatment. Plasma was obtained from the blood samples by centrifugation (6000 rpm for 3 min at 2–8 °C) and stored at −20 °C. The LC-MS/MS (Shimadzu, API 4000) method was used to determine the concentration of each animal at each time point.

#### 3.2.7. In Vivo Xenograft Research

Male BALB/c nude mice (6–8 weeks old) were purchased from Hangzhou Medical College. All animals were housed in a specific pathogen-free facility. MV-4-11 cells (5 × 10^6^) suspended in 0.2 mL of PBS were inoculated subcutaneously on the right flank of each BALB/c nude mouse. Mice were divided randomly (5 mice for each group) into two treatment groups and a control group when the size of the tumors reached 150 mm^3^. GSK-3326595 (10 mg/kg, dissolved in DMSO/10% PEG300/20% β-cyclodextrin/7%), compound 20 (10 mg/kg, DMSO/10% PEG300/20% β-cyclodextrin/7%) and vehicle (DMSO/10% PEG300/20% β-cyclodextrin/7%) were administered twice per day for 28 days by intraperitoneal administration. The tumor volume and body weight were measured every 2 days. The tumor volume was determined with Vernier calipers and calculated as follows: tumor volume = a × b^2^/2, where a and b stand for the longest and shortest diameters measured by the vernier caliper, respectively. All animal experiments have been approved by Pharmaceutical Experiment Center of China Pharmaceutical University.

#### 3.2.8. H&E Staining

The tumor tissue samples of xenograft model mice were fixed with 4% paraformaldehyde, dehydrated with ethanol, immersed in xylene, embedded in paraffin and cut into 4.0 μm longitudinal sections. The paraffin-embedded sections were stained with H&E according to the manufacturer’s instructions (Beyotime, Shanghai, China). Each group of samples was observed with a DM6B-positive fluorescence microscope (Leica, Frankfurt, Germany). Five images were randomly captured per slide.

#### 3.2.9. Immunohistochemical Staining

Tumors of xenograft model mice were embedded in paraffin, incubated with 0.3% hydrogen peroxide to block endogenous peroxidase, blocked with 1.0% BSA, stained with the primary antibody, incubated with secondary antibody and counterstained with hematoxylin. Each group was examined using the DM6B-positive fluorescence microscope (Leica, Germany). Five images were randomly captured per slide. The percentage of stained dots were analyzed by Image J software.

#### 3.2.10. Statistical Analysis

Data were analyzed using Prism 8.0. Results were expressed as means ± SD. Differences between treatment regimens were analyzed by one way ANOVA. *p* < 0.05 was considered statistical significance.

### 3.3. Molecular Modelling

The protein crystallized was downloaded from the PDB (PDB code: 4X61). Protein Preparation Wizard of the Schrodinger Suite was used to prepare protein structure to ensure that the downloaded X-ray structure was reliable and qualitatively considerable for further in silico studies. Small molecules were prepared using LigPrep [30] prior to docking simulation to obtain the accessible least-energy ionized conformer and tautomeric states of the ligands. The binding site was defined by a box centered on the centroid of the crystal ligand and with similar size to the ligand. The Glide implemented in Schrodinger 2009 was used for molecular docking [31]. The standard precision (SP) mode was used for the docking and scoring. All other parameters were kept default. The best pose was output on the basis of Glide score and the protein–ligand interactions. PyMOL software was employed to depict structural representations [32].

## 4. Conclusions

In this work, we designed and synthesized a series of THIQ derivatives. Among them, compound **20** has potent PRMT5 inhibitory activity, with the IC_50_ value of 4.2 nM. In addition, compound **20** also dramatically suppresses the growth of MV-4-11 and MDA-MB-468 cell lines with no significant cytotoxicity in normal hepatocyte AML-12 cell lines. In MV-4-11 cell-derived xenograft model, compound **20** (TGI = 47.6%) at a dose of 10 mg/kg (iv) displays more effective antitumor effects than **GSK-3326595** (TGI = 39.3%) at the same dose (iv). The preliminary mechanism studies in vivo confirm that **20** exerts antitumor effect via decreasing the expression of sDMA in tumor tissues. Encouraging to researchers owing to the excellent properties in vitro and in vivo, **20** as a novel PRMT5 inhibitor is worthy of further study.

## Figures and Tables

**Table 1:** In Vitro PRMT5 Inhibitory activity of compounds **1**~**6**.

Compound	R^1^	R^2^	Inhibition Rate%@100 nM	IC_50_ (nM)
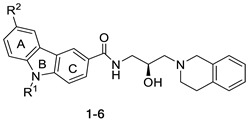				
**1**	H	H	73 ± 0.7	13 ± 8.9
**2**	Me	H	59 ± 0.4	ND
**3**	F	H	65 ± 0.2	ND
**4**	OCH_3_	H	64 ± 1.5	ND
**5**	Cl	H	69 ± 7.0	ND
**6**	H	Me	63 ± 3.2	ND
**GSK-3326595**			77 ± 3.8	9.2 ± 0.1

**Table 2:** In Vitro PRMT5 Inhibitory activity of compounds **7~26**.

Compound	R^1^	R^2^	X	P	Inhibition Rate% @100 nM	IC_50_ (nM)
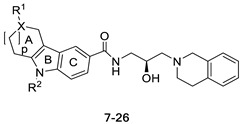						
**7**	H	H	C	1	75 ± 0.7	18.6 ± 0.9
**8**	H	H	C	0	74 ± 2.0	ND
**9**	H	H	C	2	73 ± 4.1	ND
**10**	H	H	C	3	71 ± 1.3	ND
**11**	Me	H	C	1	67 ± 3.5	ND
**12**	Et	H	C	1	58 ± 3.7	ND
**13**	H	Me	C	1	75 ± 0.7	10 ± 4.4
**14**	H	Et	C	1	81 ± 3.2	8.8 ± 0.4
**15**	H	*n*-Pr	C	1	79 ± 4.0	4.2 ± 2.1
**16**	H	*i*-Pr	C	1	70 ± 0.2	ND
**17**	H	*i*-Bu	C	1	75 ± 1.6	ND
**18**	H	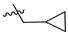	C	1	60 ± 4.6	ND
**19**	H	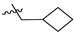	C	1	80 ± 1.0	7.5 ± 0.3
**20**	H	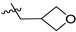	C	1	83 ± 12	4.2 ± 1.3
**21**	Me	H	N	1	48 ± 1.2	ND
**22**	Et	H	N	1	64 ± 10.7	ND
**23**	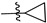	H	N	1	52 ± 4.8	ND
**24**	*n*-Pr	H	N	1	63 ± 4.4	ND
**25**	*i*-Pr	H	N	1	51 ± 4.2	ND
**26**	*i*-Bu	H	N	1	59 ± 1.4	ND
**GSK-3326595**					77 ± 3.8	9.2 ± 0.1

**Table 3:** Anti-proliferative activity of compounds in vitro.

Compound	MV4-11 (GI_50_, nM)	MDA-MB-468 (GI_50_, nM)	AML-12	
			Inhibition Rate% @10 μM	Inhibition Rate% @20 μM
**1**	271.3 ± 151.7	552.1 ± 33.0	16.7 ± 10.4	97.5 ± 0.1
**7**	54.1 ± 20.5	>250	46.4 ± 3.7	98.4 ± 0.7
**10**	42.4 ± 2.6	125.8 ± 70.9	97.9 ± 1.2	100.7 ± 0.1
**13**	163.1 ± 0.3	147 ± 2.5	16.3 ± 3.4	21.2 ± 2.0
**14**	29.7 ± 12.8	285.7 ± 5.9	−67 ± 11.3	17.7 ± 3.5
**15**	39.5 ± 1.9	24.9 ± 20.2	−21.5 ± 2.0	77.3 ± 1.6
**19**	16 ± 2.3	99.1 ± 8.3	32.9 ± 5.4	100.2 ± 0.9
**20**	4.6 ± 0.9	9.9 ± 6.8	−30.5 ± 9.4	−27.0 ± 1.2
**GSK-3326595**	9.3 ± 2.8	37.3 ± 3.0	22.9 ± 3.0	26.5 ± 2.0

**Table 4:** Pharmacokinetics (PK) of Compound **20** *.

Route	Species	C_max_(ng/mL)	T_1/2_(h)	AUC_0−∞_(h·ng/mL)	V_ss_(mL/kg)	CL(mL/h/kg)	F (%)
po.	mouse	1267	6.06	747			14.5
iv.	mouse	1687	0.49	1031	1346	1944	

* *n* = 3. The compound was formulated as solution in 20% HP-β-CD in saline. Dosage: 2 mg/kg for intravenous (iv) and 10 mg/kg for oral (po)administration. ICR mouse were used. The maximum drug concentration (C_max_) was observed at t = 5 min, the first sampling time point after iv administration.

## Data Availability

The article contains complete data used to support the findings of this study.

## Supplementary Materials

The following supporting information can be downloaded at: https://www.mdpi.com/article/10.3390/molecules27196637/s1. Figure S1: Plasma concentration curve after administration of **20** (2 mg/kg, iv). The value at each time point represents. Figure S2: Plasma concentration curve after administration of **20** (10 mg/kg, ig). The value at each time point represents. Table S1: intravenous injection of 2 mg/kg compound **20** in male mice pharmacokinetic parameters. Table S2: gavage of 10 mg/kg compound **20** in male mice pharmacokinetic parameters.

## Sample Availability

Samples of the compounds described in this paper are available from the authors.
