# Supplementary material for: Design, Synthesis and Biological Evaluation of Novel and Potent Protein Arginine Methyltransferases 5 Inhibitors for Cancer Therapy
Source: Molecules. 2022 Oct 6;27(19):6637. doi: 10.3390/molecules27196637 (PMC9572541; doi:10.3390/molecules27196637)

Supplementary Materials

1. Pharmacokinetics

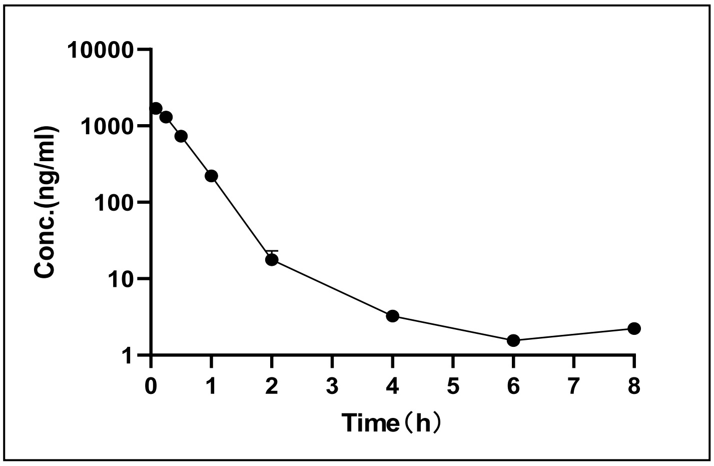

Figure S1. Plasma concentration curve after administration of **20** (2 mg/kg, iv). The value at each time point represents

Table S1. Intravenous injection of 2 mg/kg compound **20** in male mice pharmacokinetic parameters (n=3 per group).

Parameters	Mean ± SD
$T_{1/2}$ (hr)	0.49 ± 0.23
T_{max} (hr)	0.08 ± 0.00
C_{max} (ng/mL)	1686.67 ± 133.17
C_0 (ng/mL)	1936.74 ± 330.29
AUC_{0-t} (hr*ng/mL)	1026.89 ± 68.51
$AUC_{0-\infty}$ (hr*ng/mL)	1031.66 ± 64.77
Vz (mL/kg)	1346.90 ± 555.73
Cl (mL/hr/kg)	1943.71 ± 121.37
MRT_{0-t} (hr)	0.44 ± 0.05
$MRT_{0-\infty}$(hr)	0.45 ± 0.05

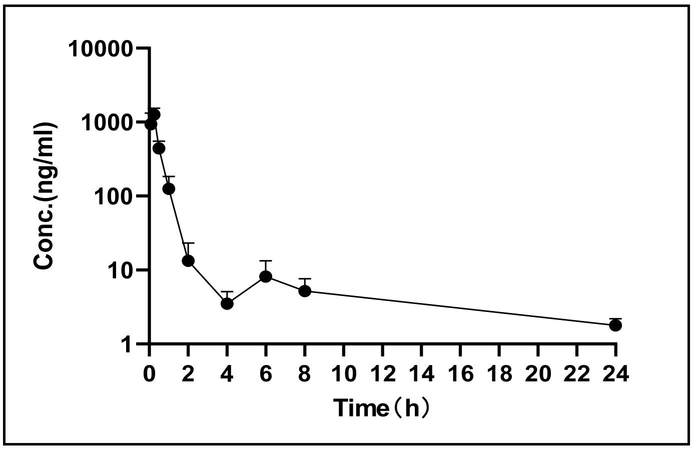

Figure S2. Plasma concentration curve after administration of **20** (10 mg/kg, po). The value at each time point represents

Table S2. Oral gavage of 10 mg/kg compound **20** in male mice pharmacokinetic parameters (n=3 per group).

**Parameters**	**Mean ± SD**
$T_{1/2}$ (hr)	6.06±3.21
T_{max} (hr)	0.25±0.00
C_{max} (ng/mL)	1266.67±273.92
AUC_{0-t} (hr*ng/mL)	727.75±234.35
$AUC_{0-\infty}$ (hr*ng/mL)	747.32 ±240.27
MRT_{0-t} (hr)	1.22±0.42
$MRT_{0-\infty}$ (hr)	1.91±0.77
F (%)	14.49%±0.05

3. ¹H NMR, ¹³C NMR and HRMS Spectra of All Target Compounds.

¹H-NMR of compound **1**

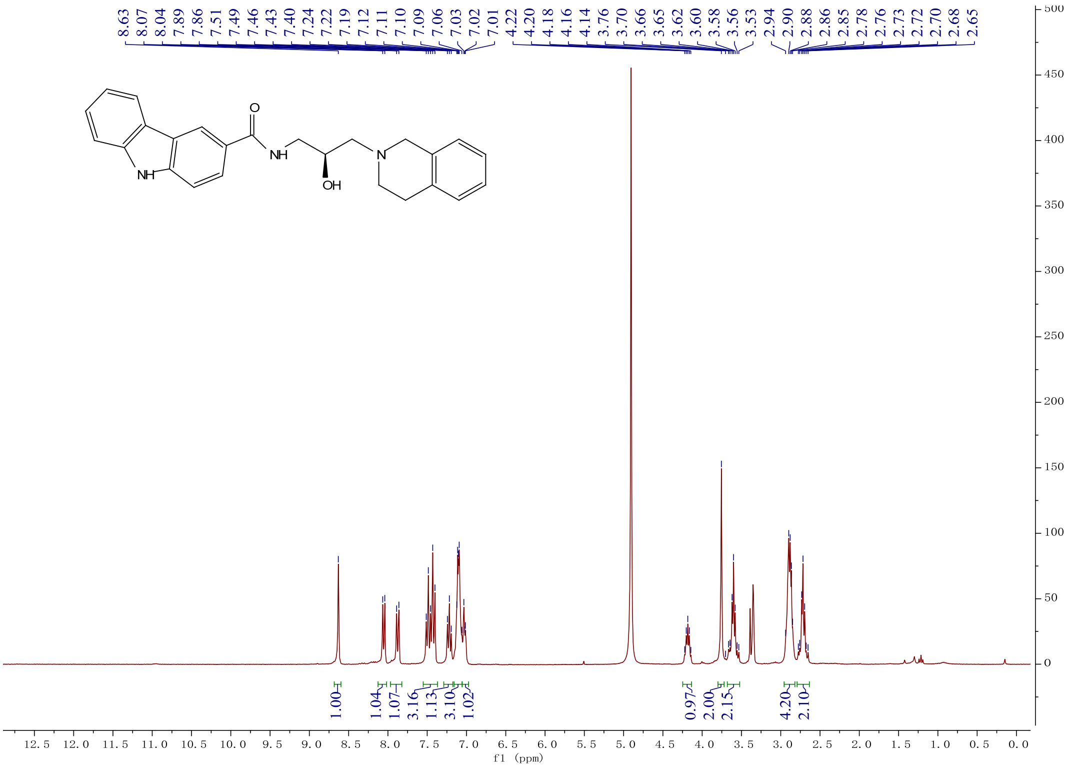

¹³C-NMR of compound **1**

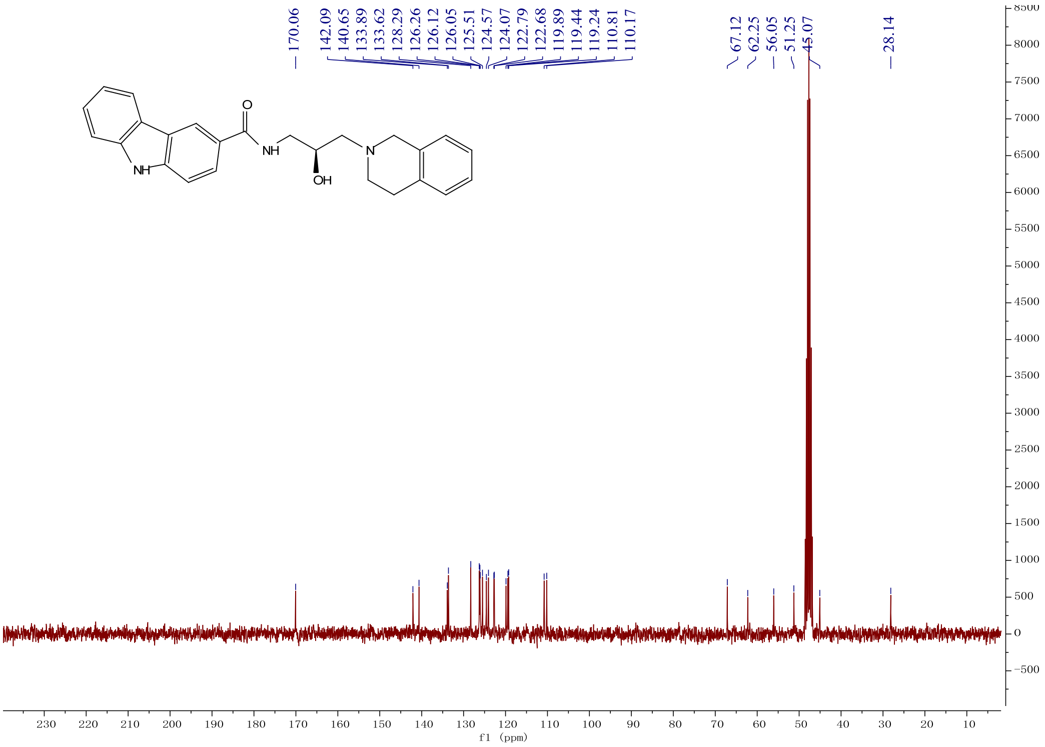

HRMS of compound **1**

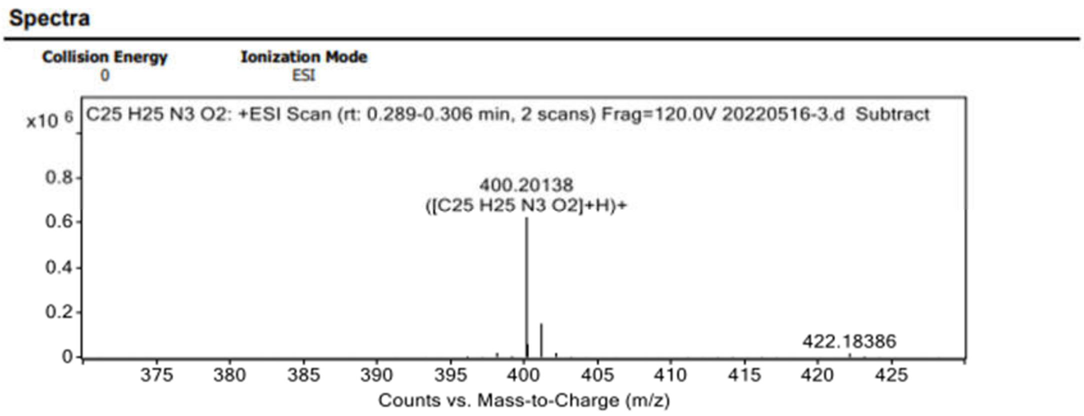

^{1}H-NMR of compound **2**

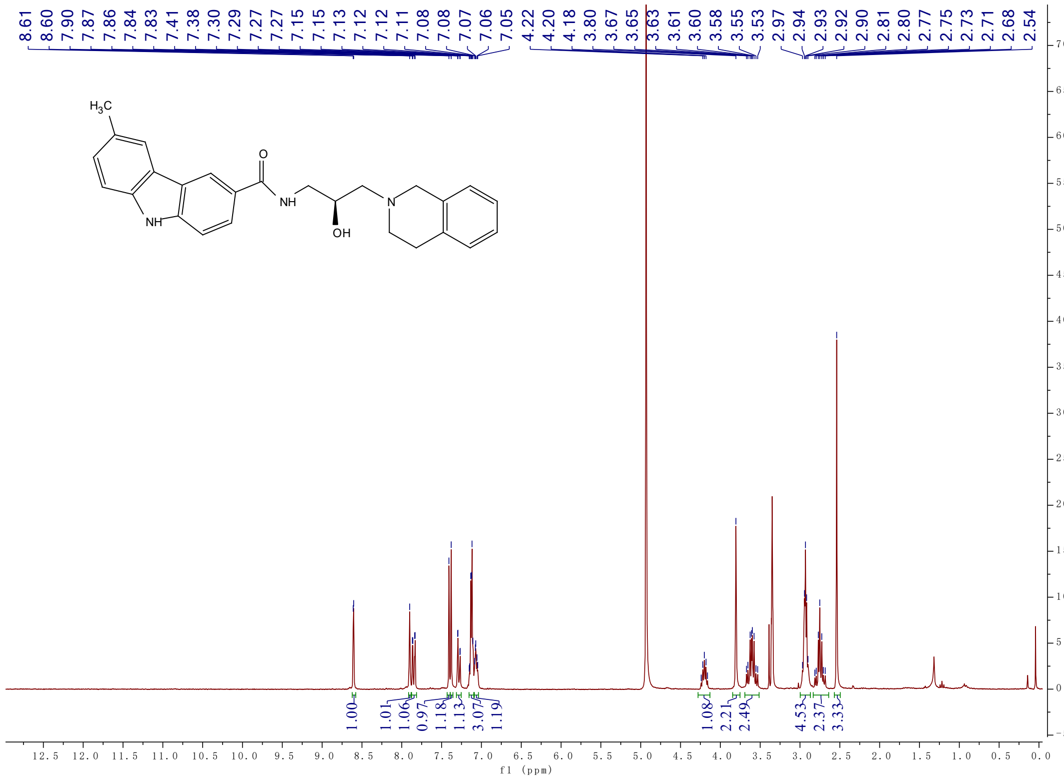

^{13}C-NMR of compound **2**

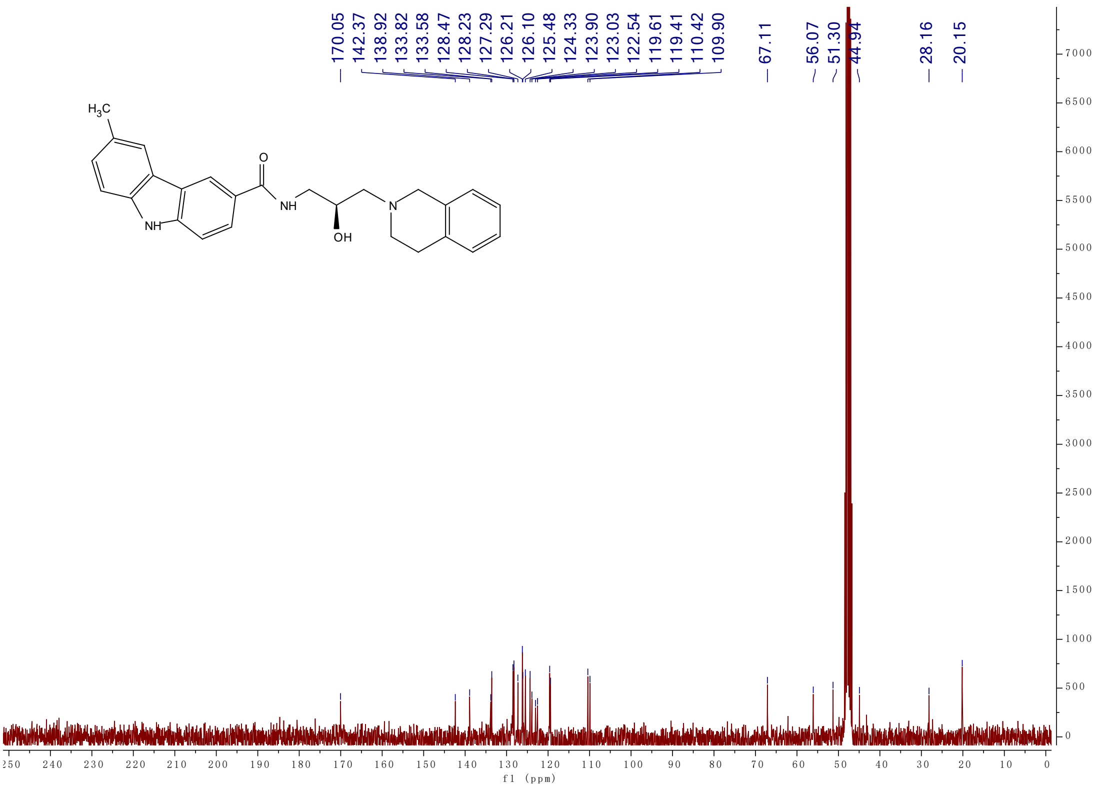

HRMS of compound **2**

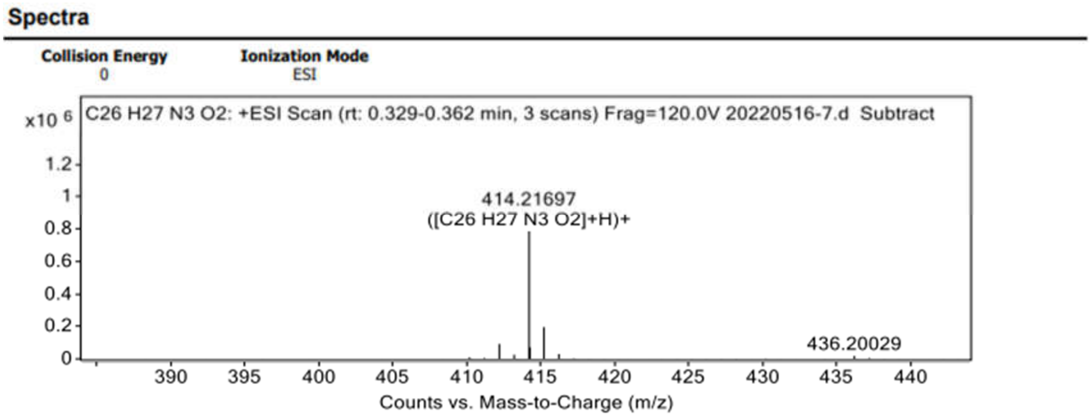

^{1}H-NMR of compound **3**

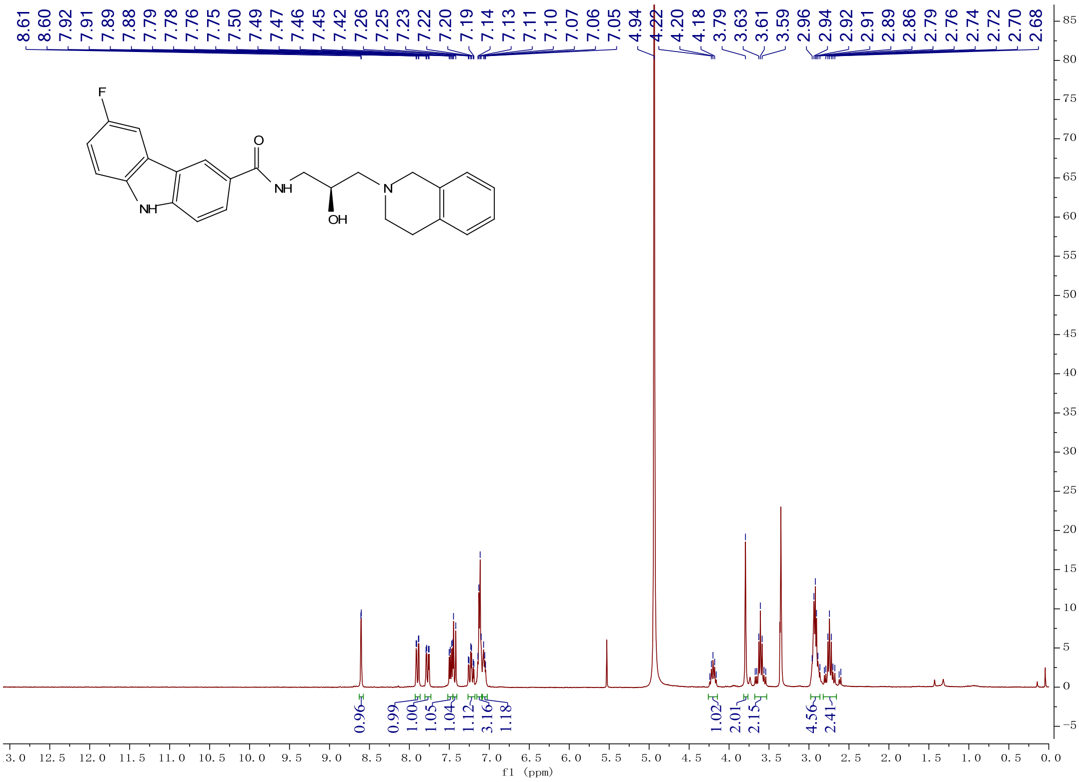

^{13}C-NMR of compound **3**

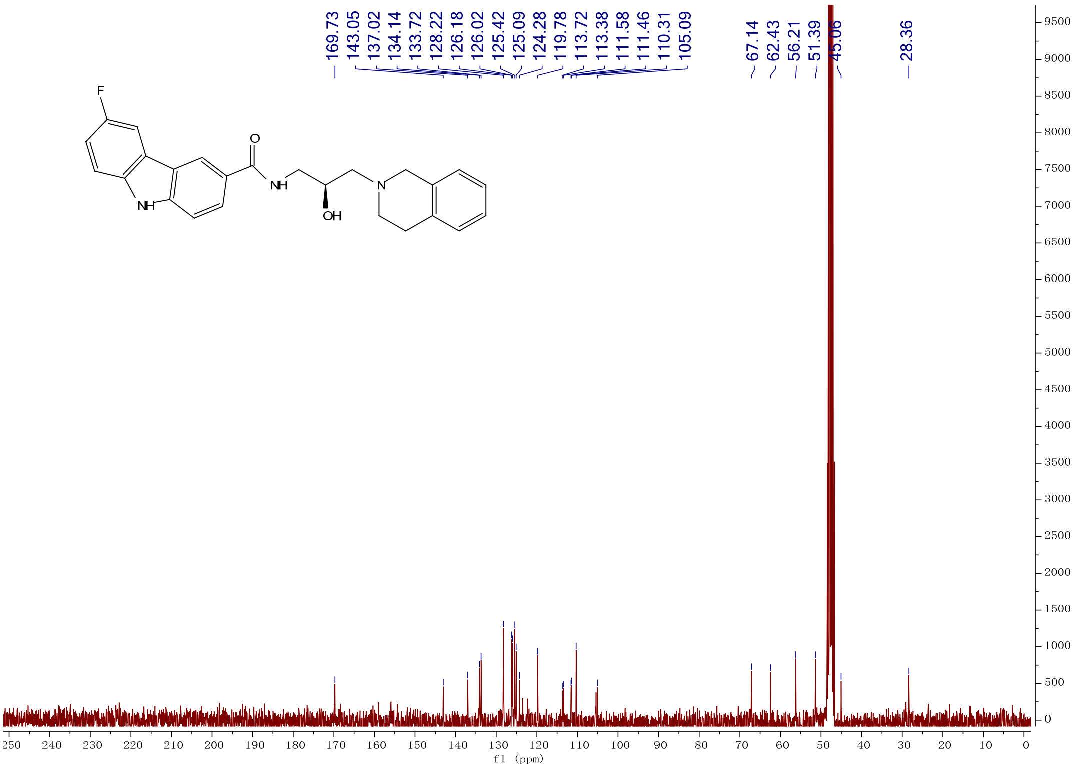

HRMS of compound **3**

Spectra

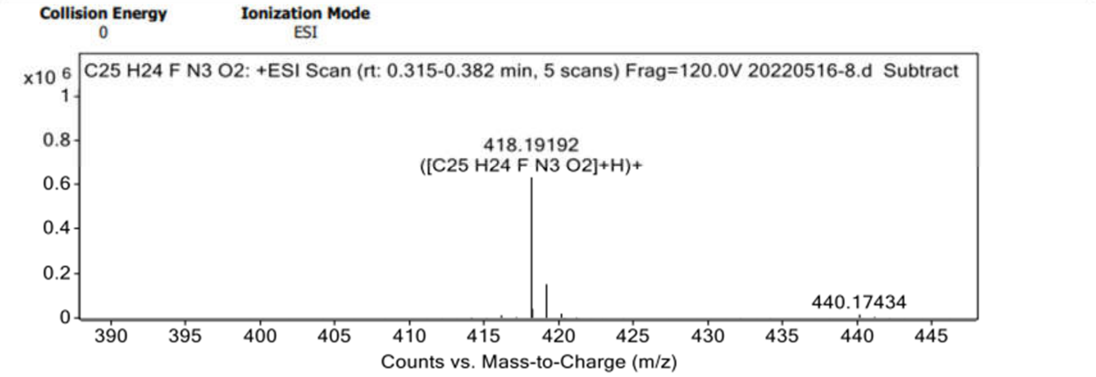

^{1}H-NMR of compound **4**

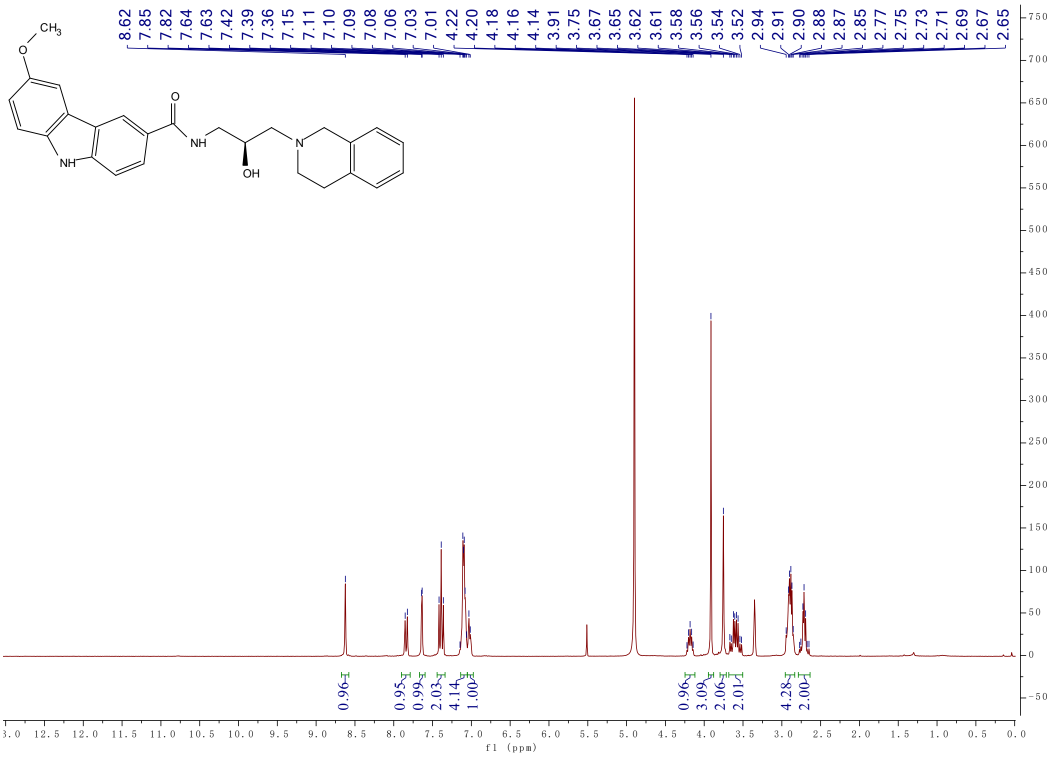

^{13}C-NMR of compound **4**

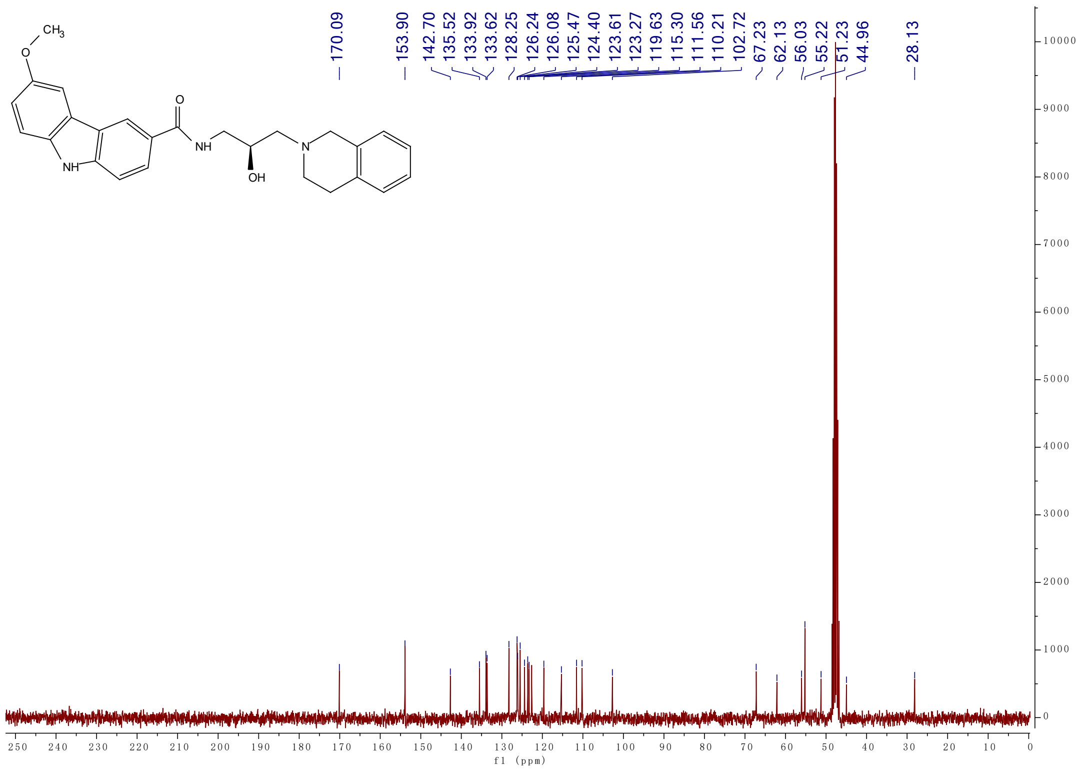

HRMS of compound **4**

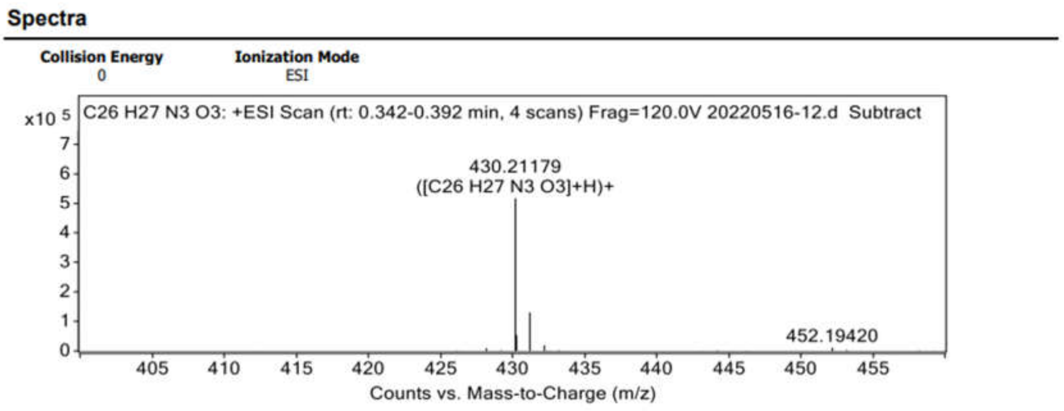

^{1}H-NMR of compound **5**

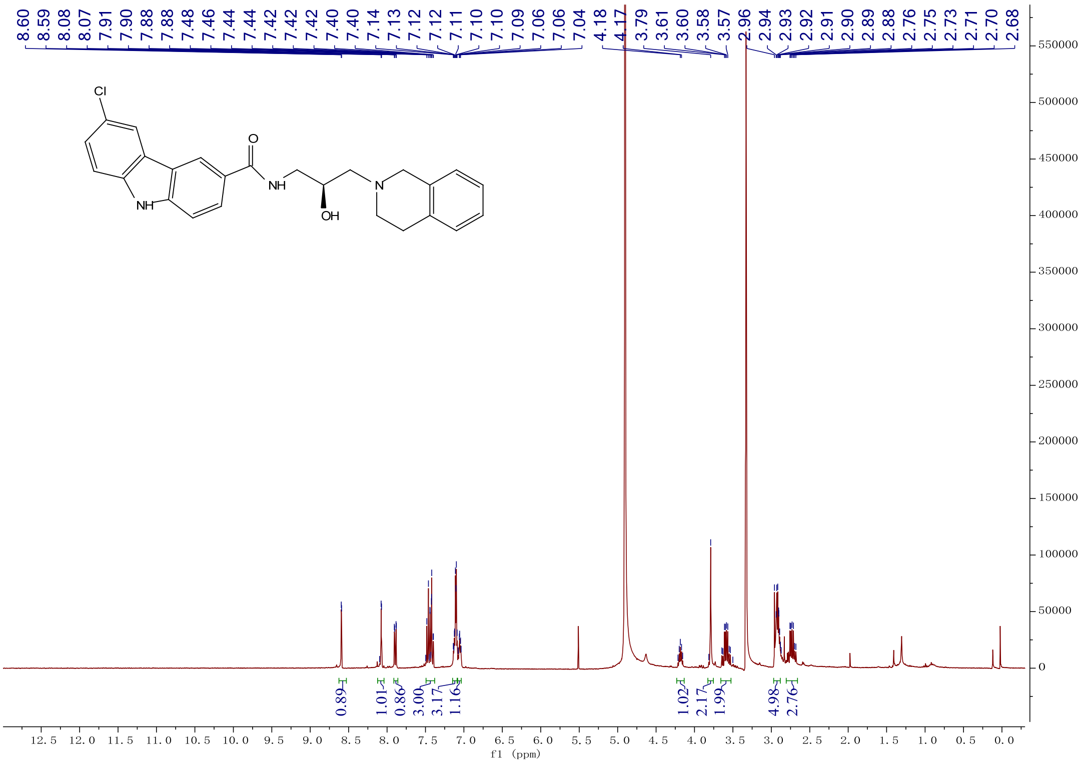

^{13}C-NMR of compound **5**

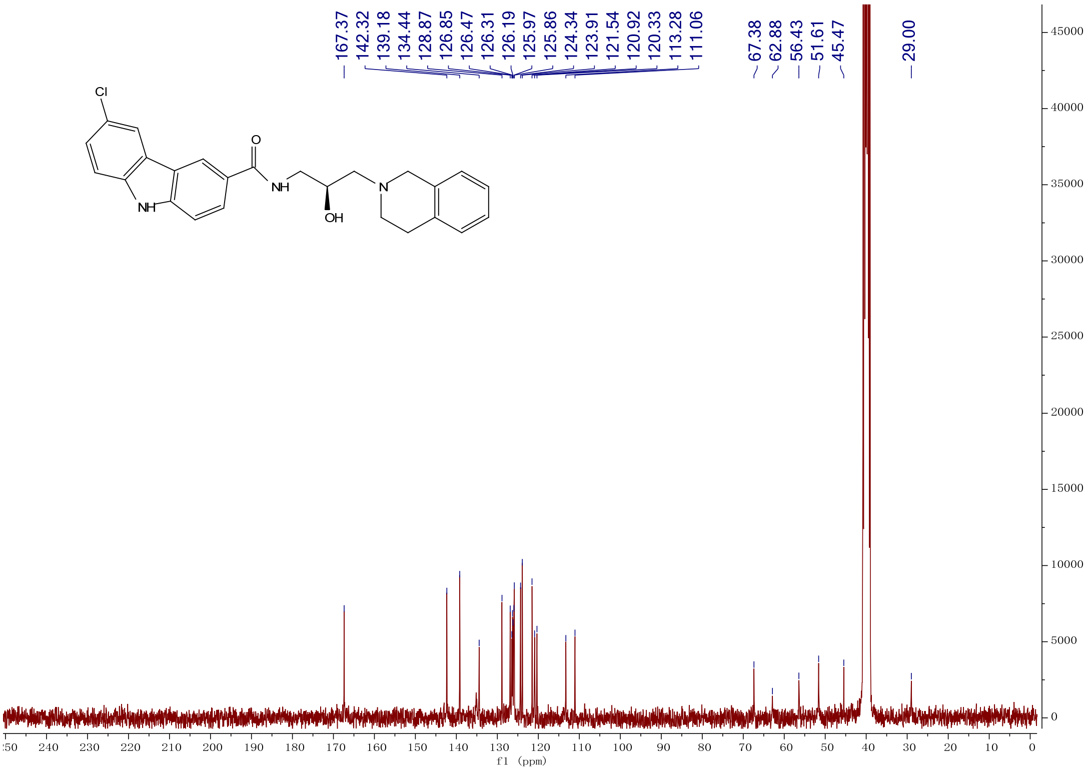

HRMS of compound **5**

Spectra

Collision Energy	Ionization Mode
0	ESI

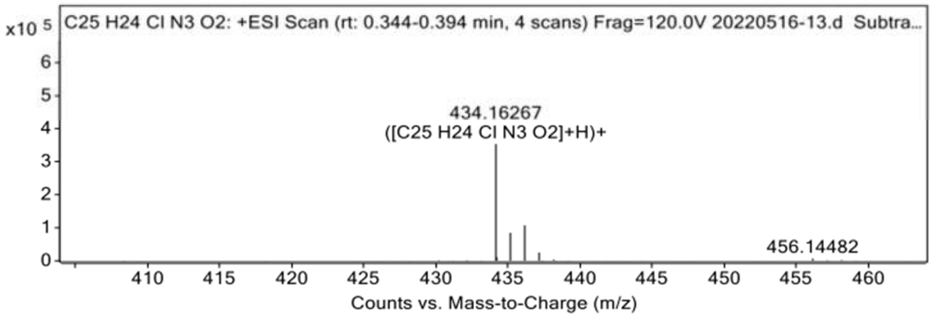

^{1}H-NMR of compound **6**

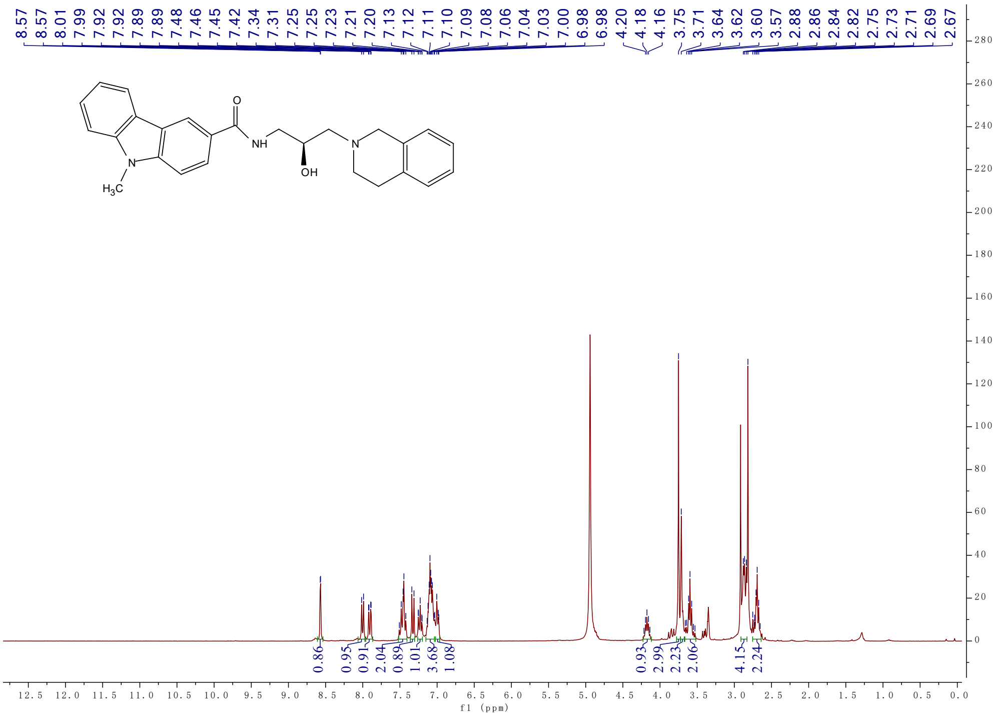

^{13}C-NMR of compound **6**

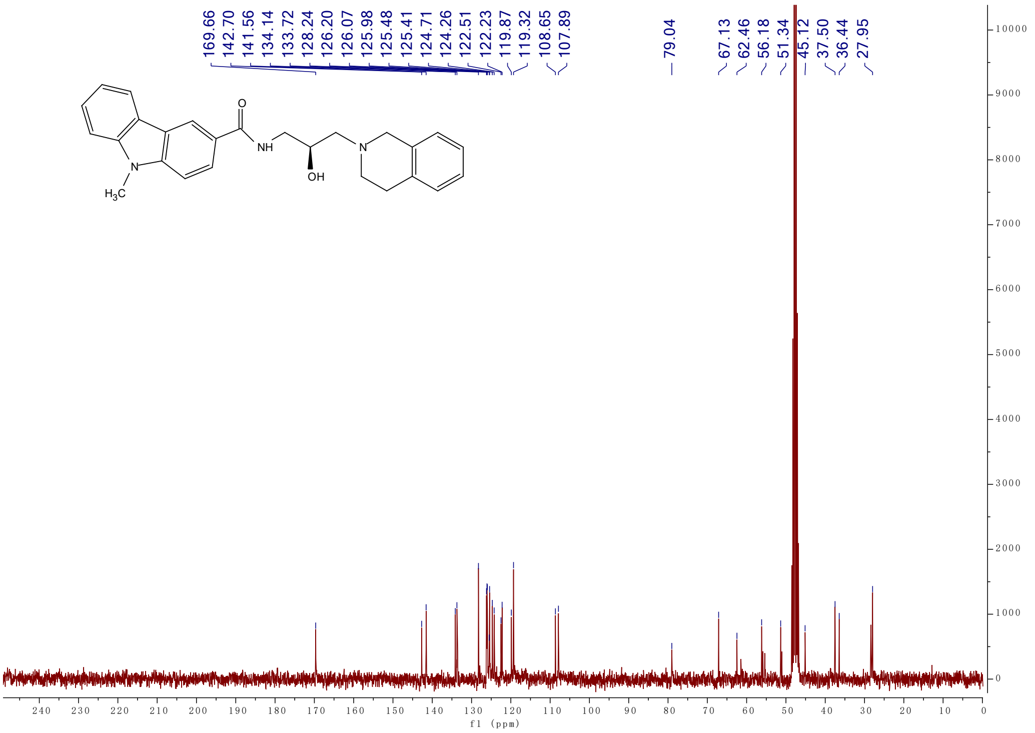

HRMS of compound **6**

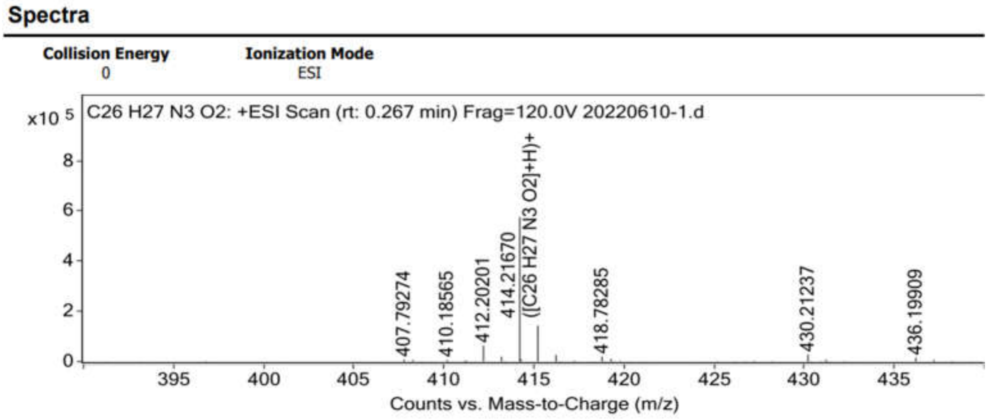

^{1}H-NMR of compound **7**

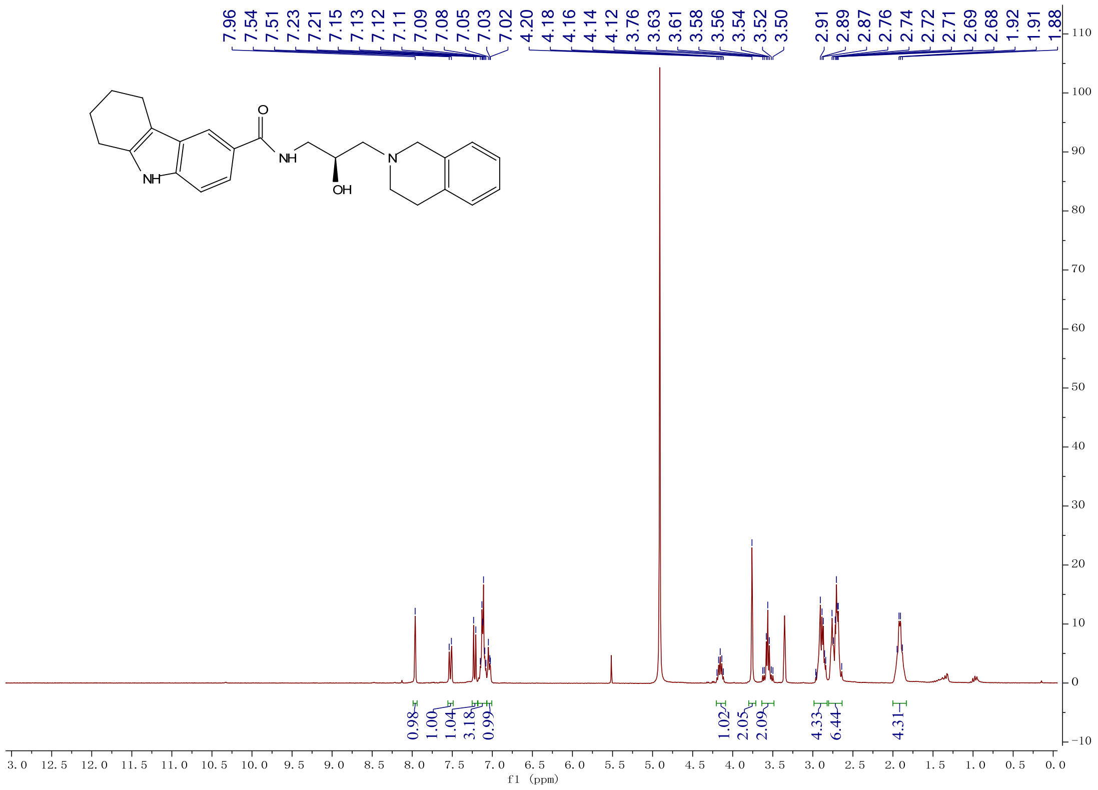

^{13}C-NMR of compound **7**

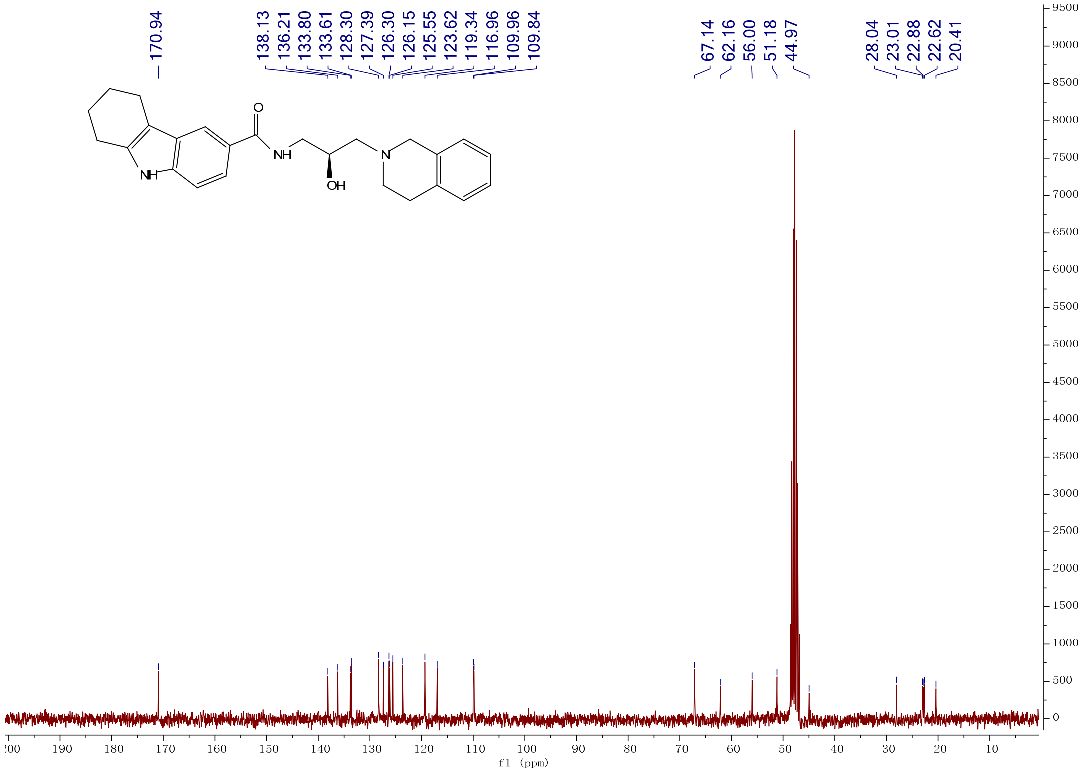

HRMS of compound **7**

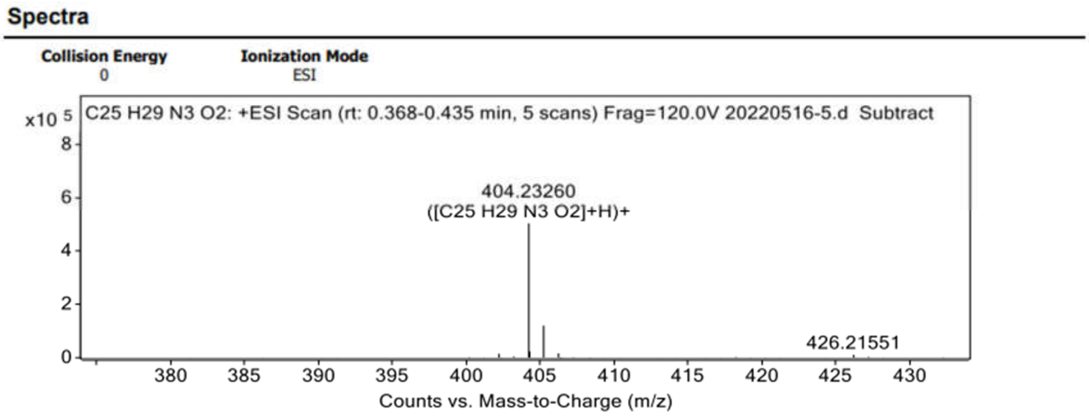

^{1}H-NMR of compound8

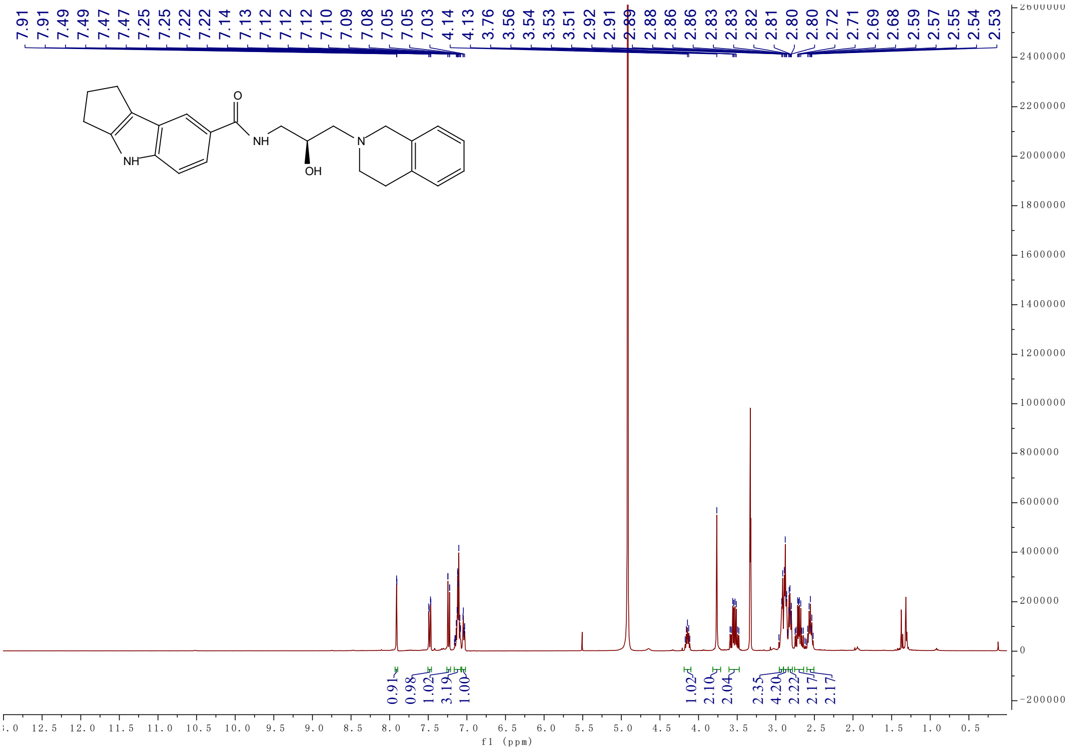

^{13}C-NMR of compound **8**

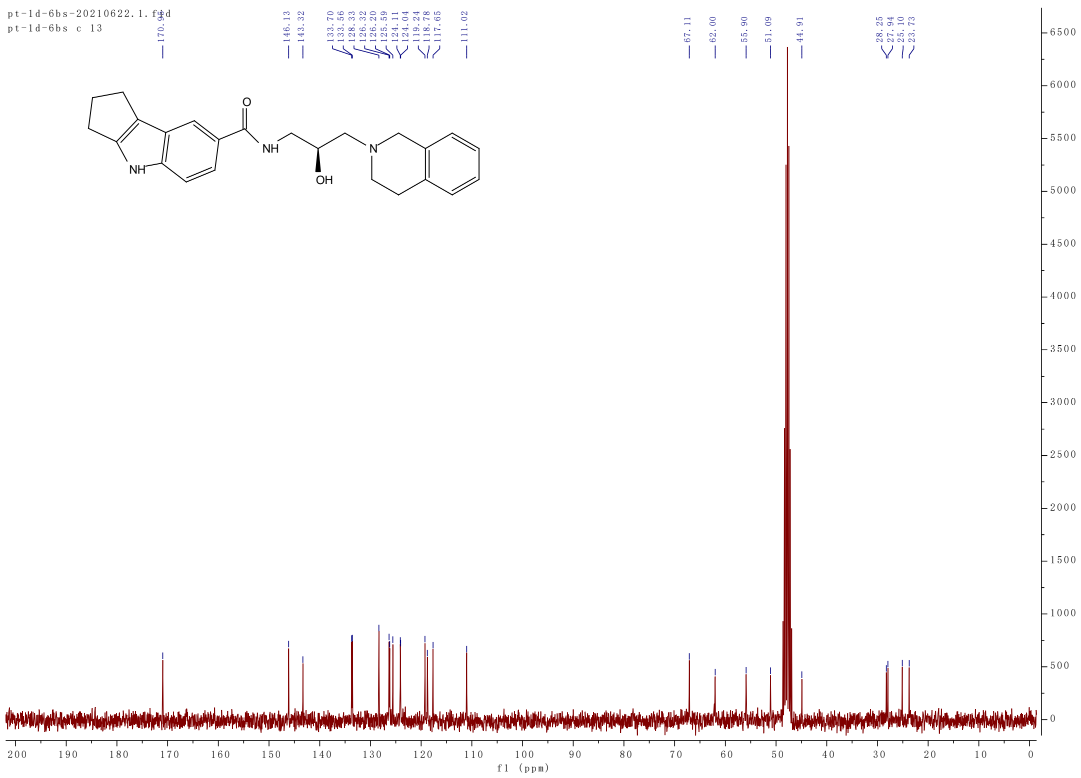

HRMS of compound **8**

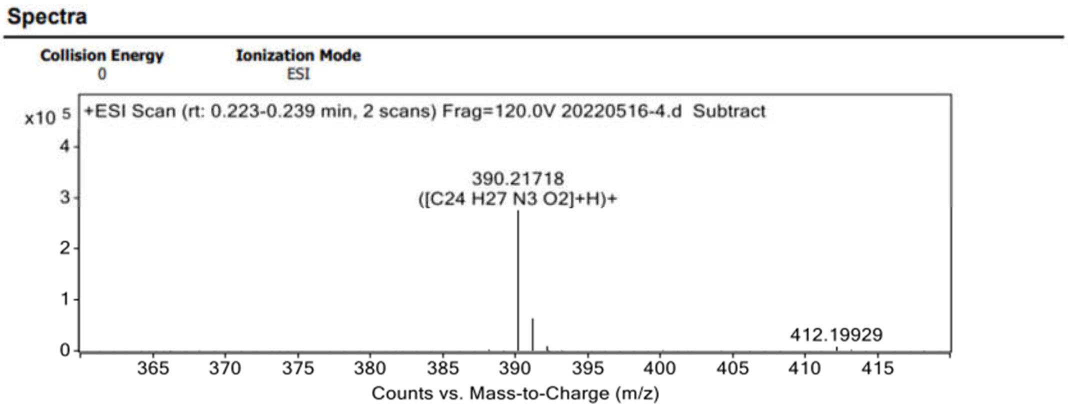

^{1}H-NMR of compound **9**

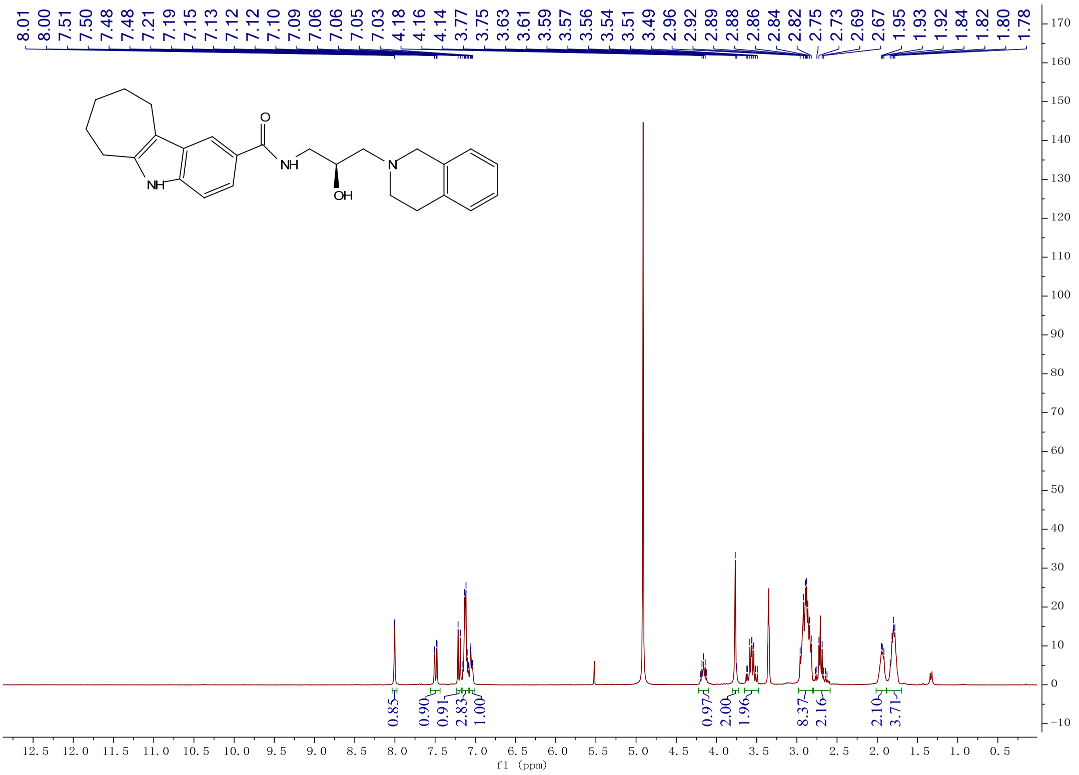

^{13}C-NMR of compound **9**

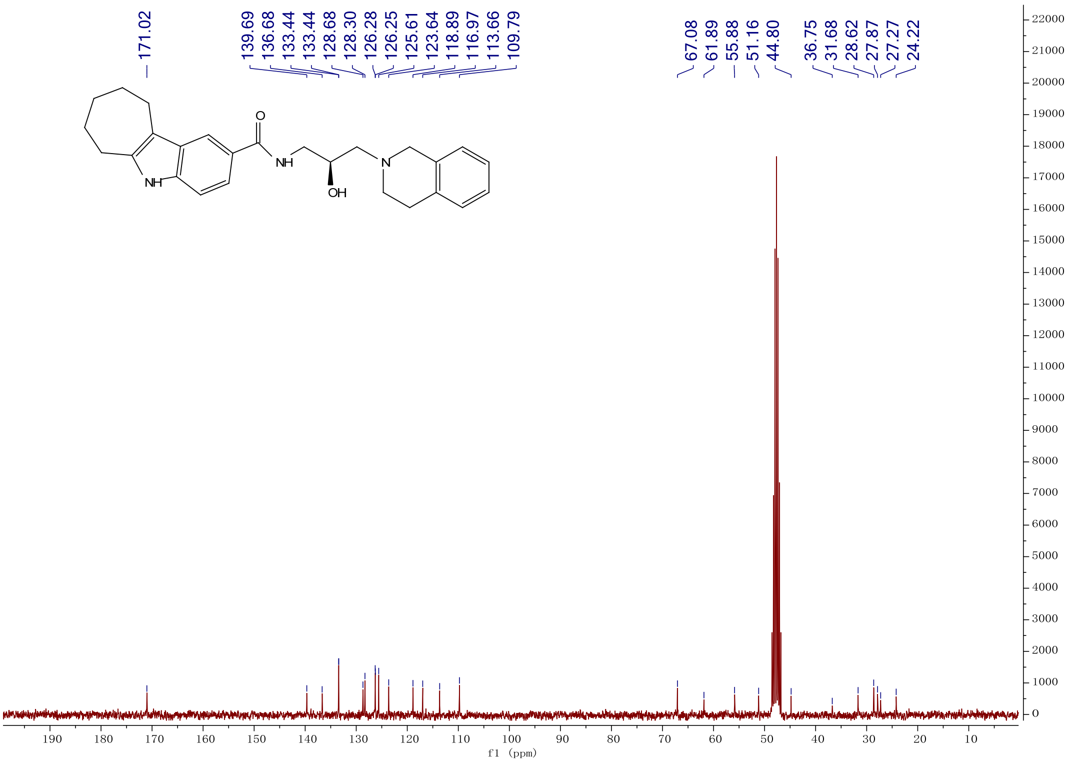

HRMS of compound **9**

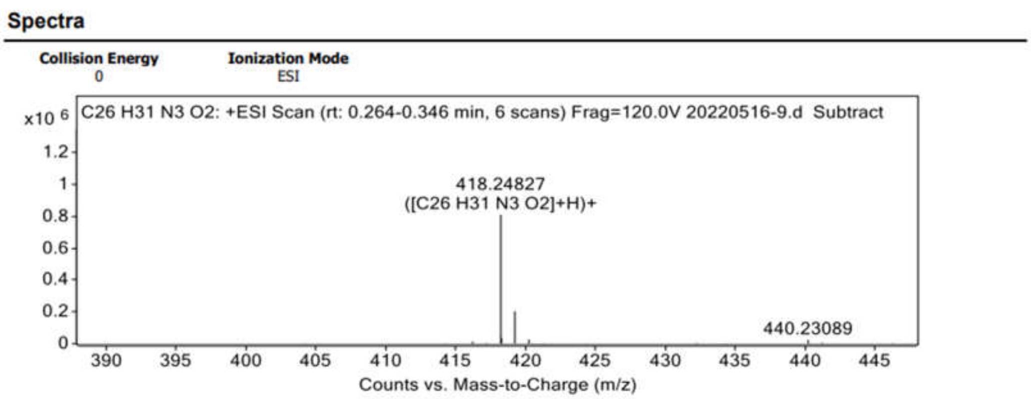

^{1}H-NMR of compound **10**

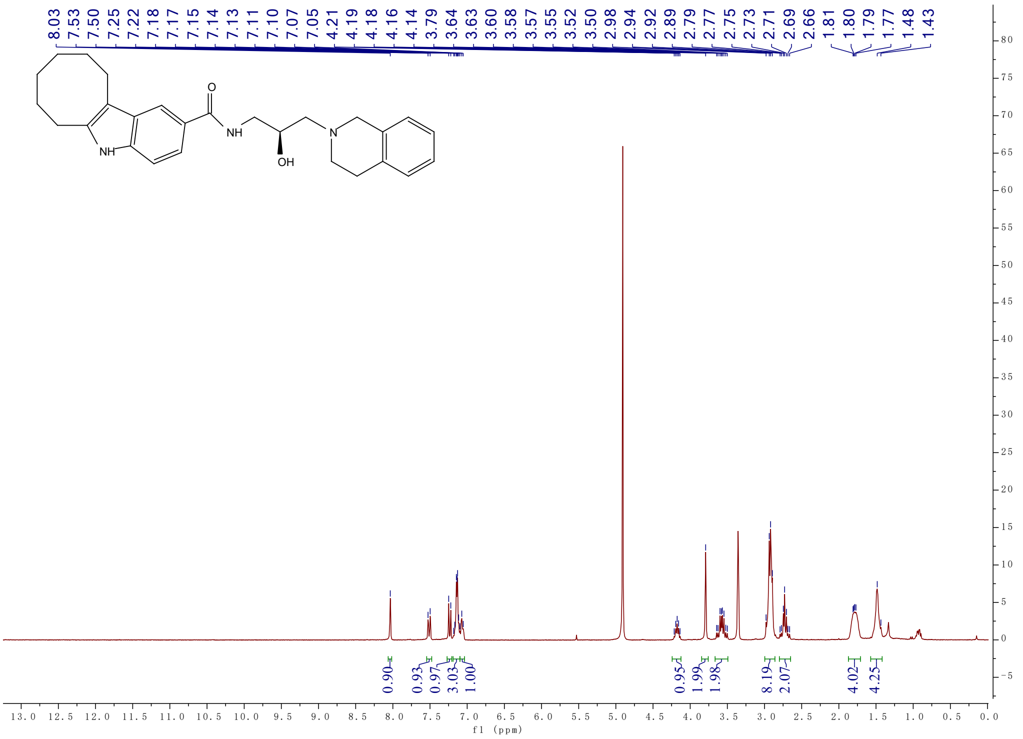

^{13}C-NMR of compound **10**

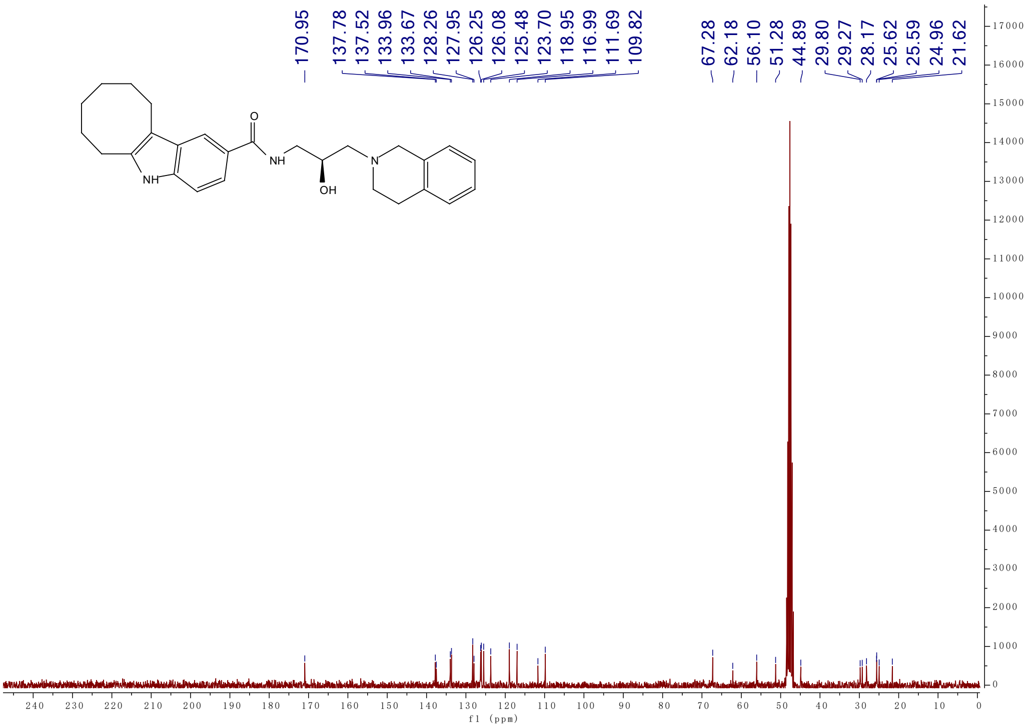

HRMS of compound **10**

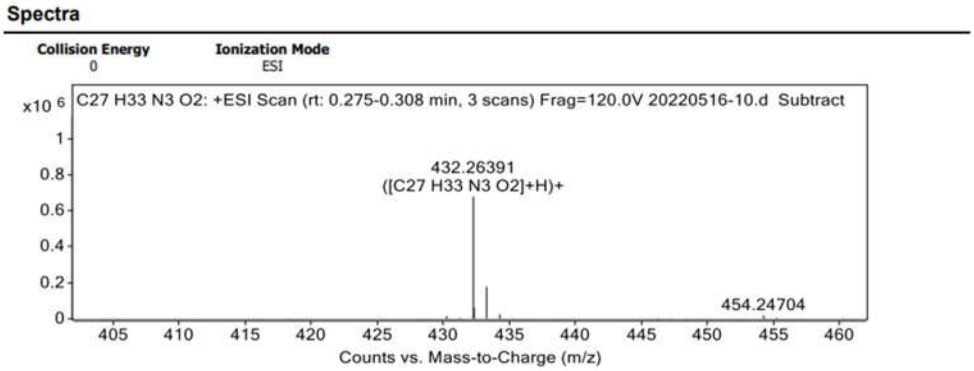

^{1}H-NMR of compound **11**

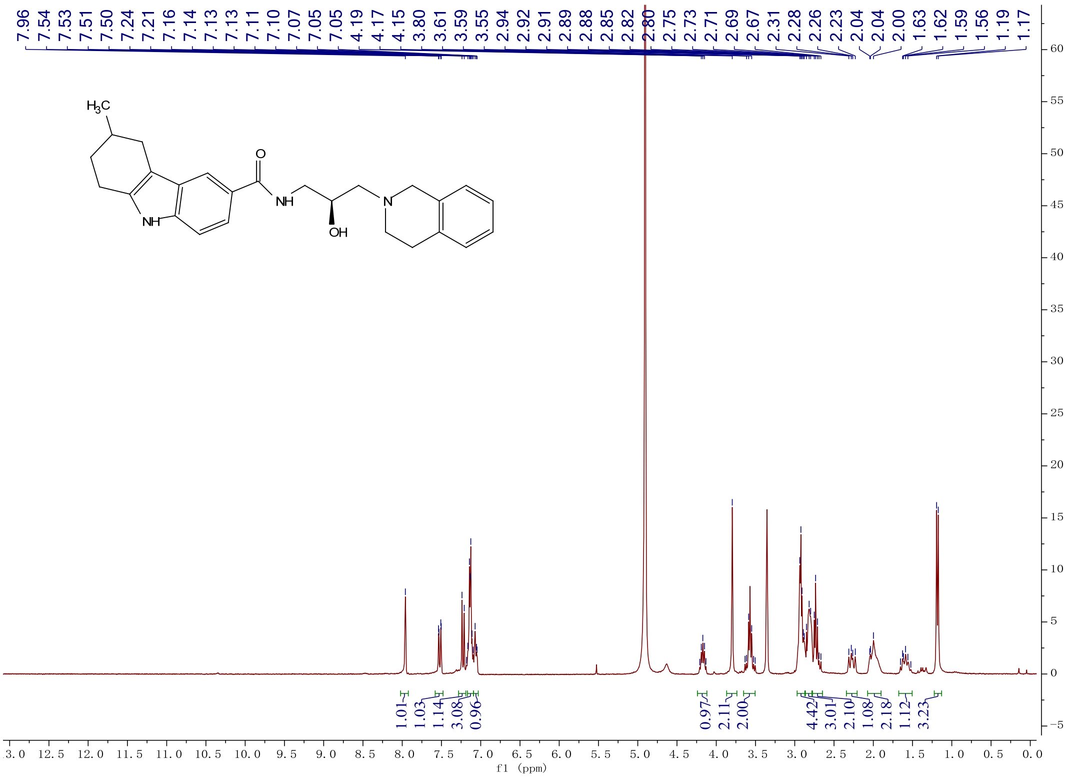

^{13}C-NMR of compound **11**

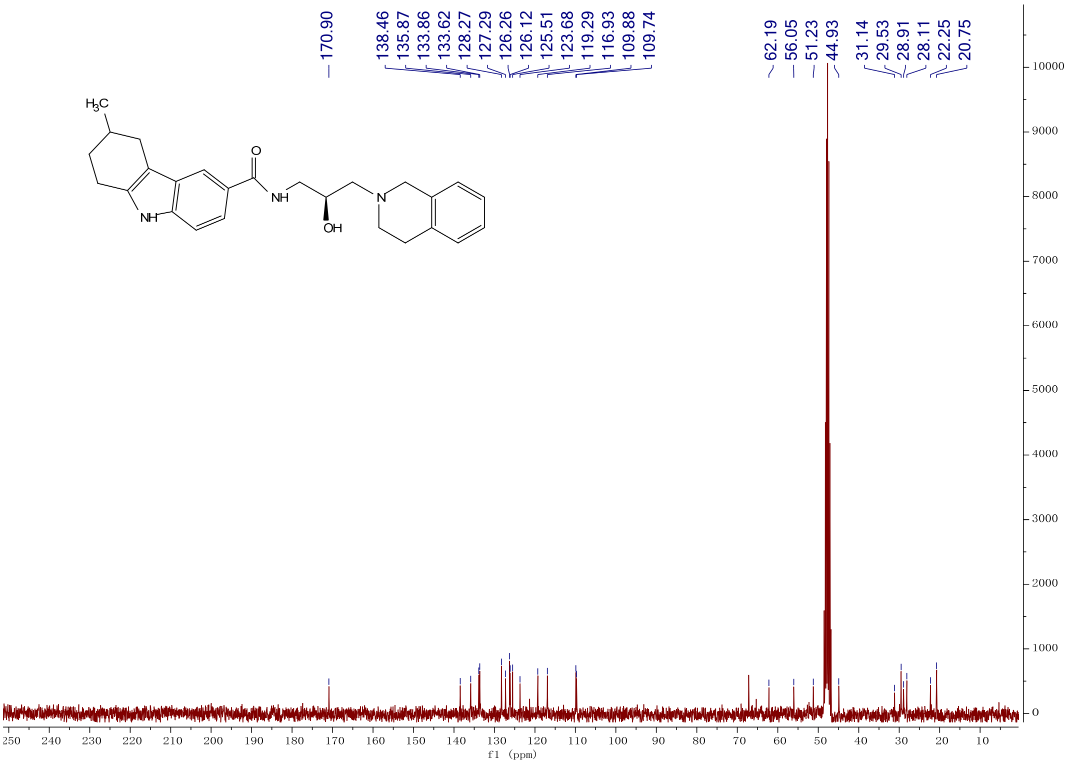

HRMS of compound **11**

Spectra

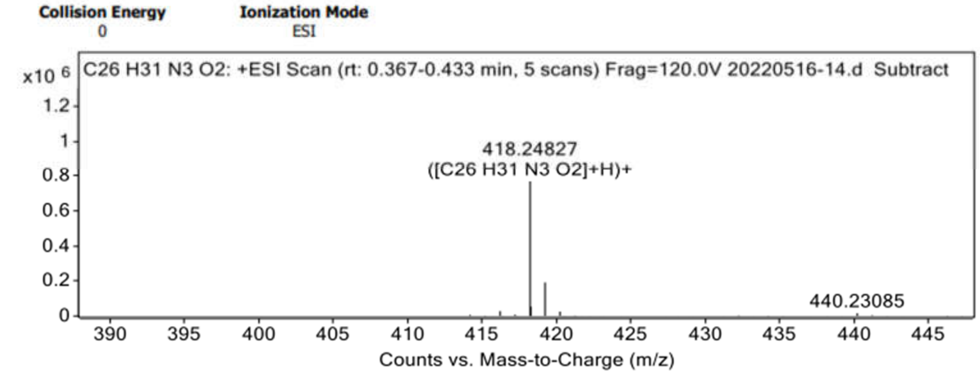

^{1}H-NMR of compound **12**

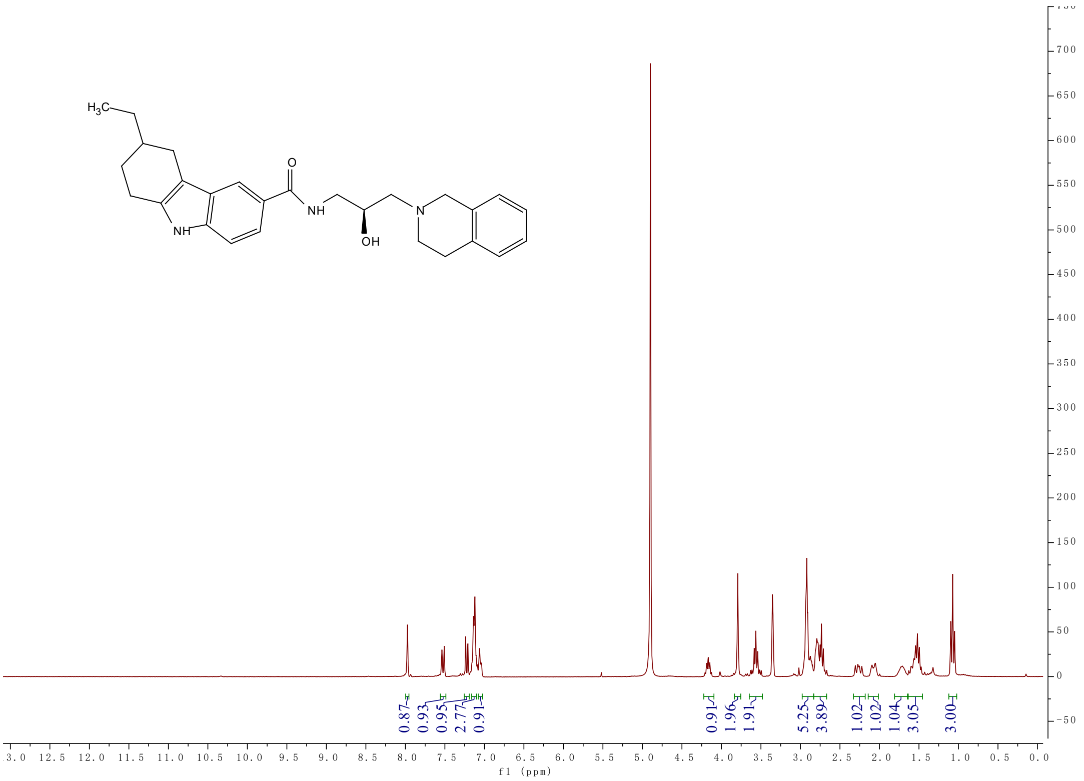

^{13}C-NMR of compound **12**

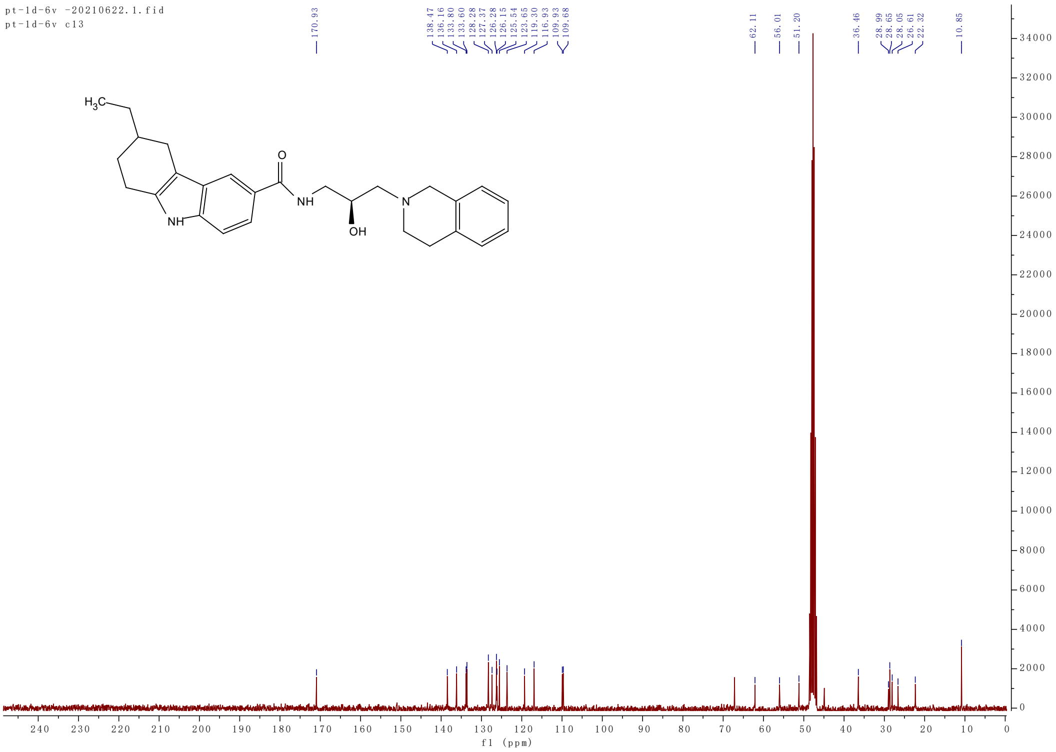

HRMS of compound **12**

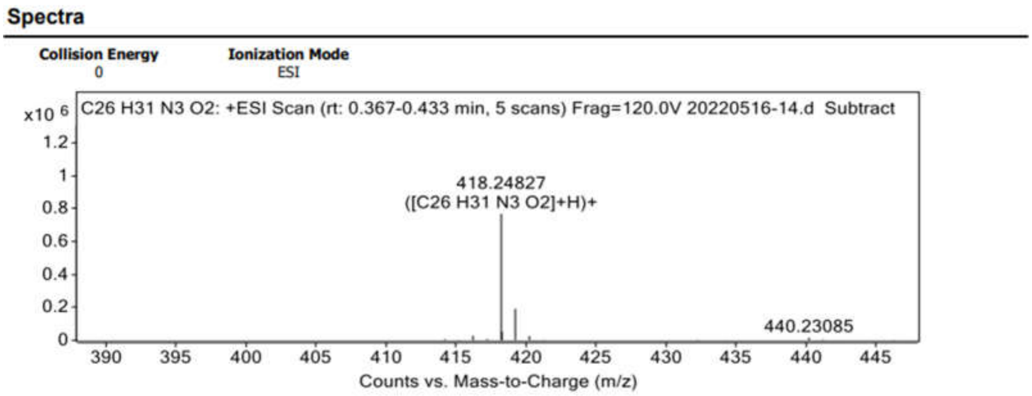

^{1}H-NMR of compound **13**

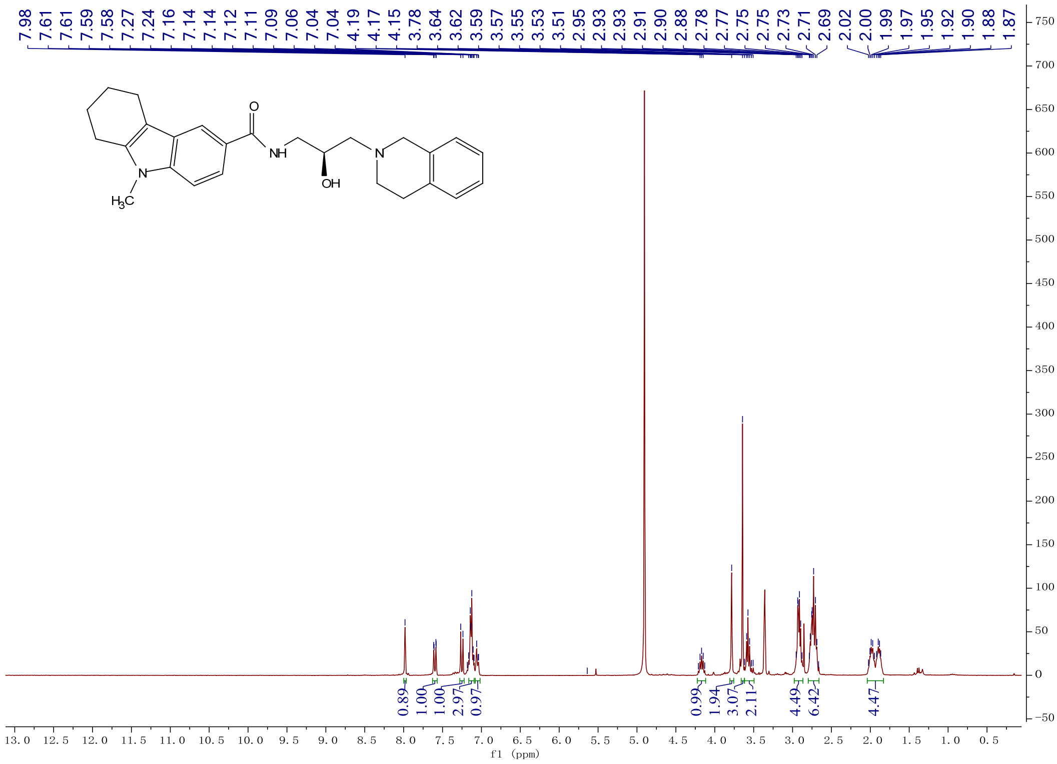

^{13}C-NMR of compound **13**

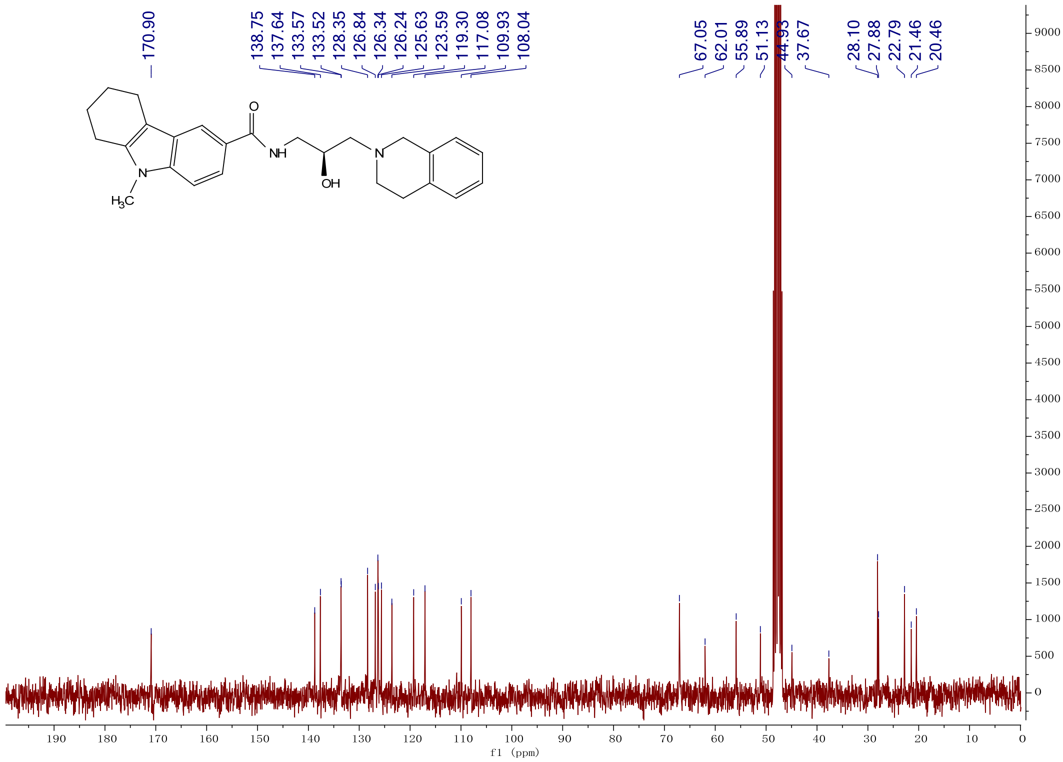

HRMS of compound **13**

Spectra

Collision Energy	Ionization Mode
0	ESI

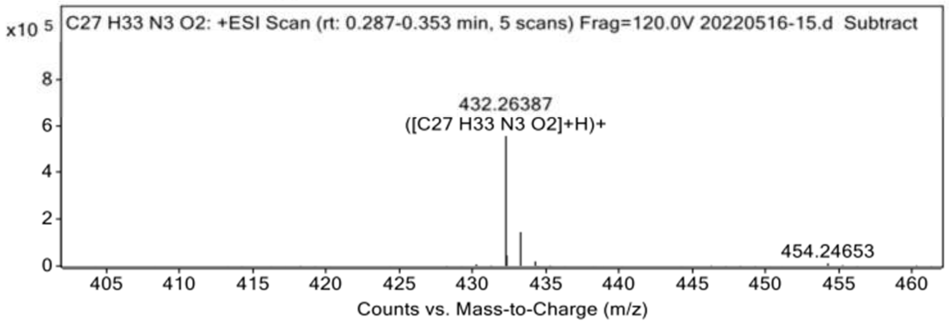

^{1}H-NMR of compound **14**

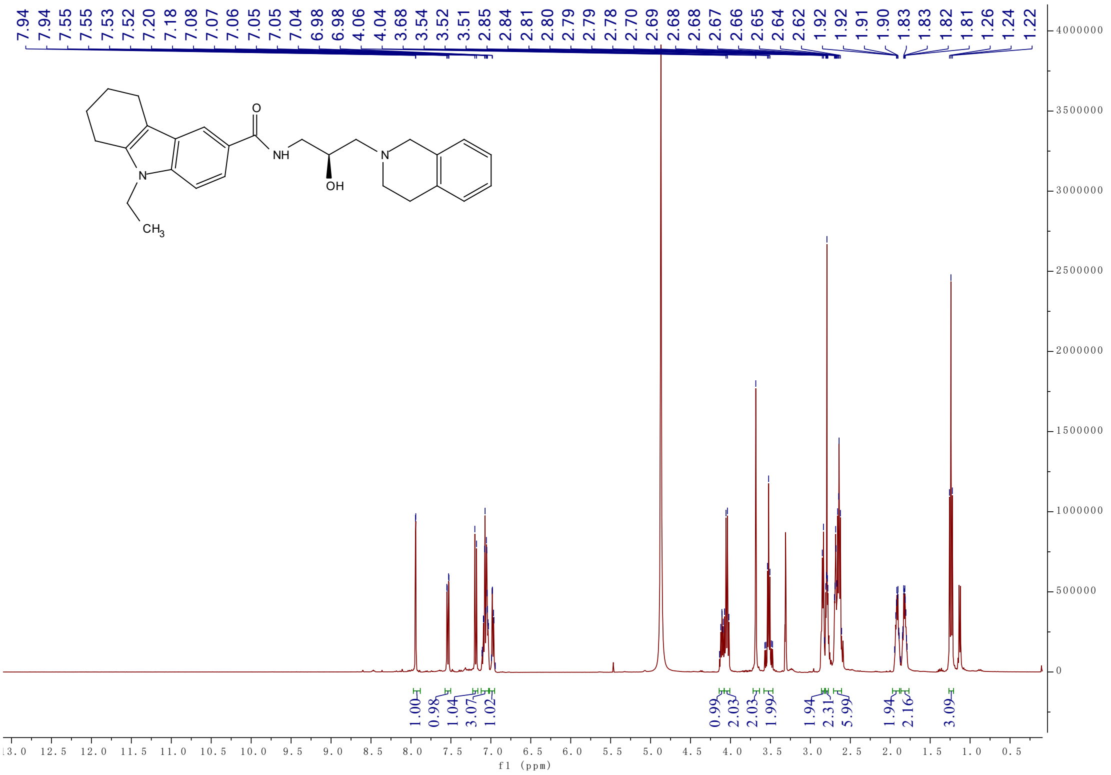

^{13}C-NMR of compound **14**

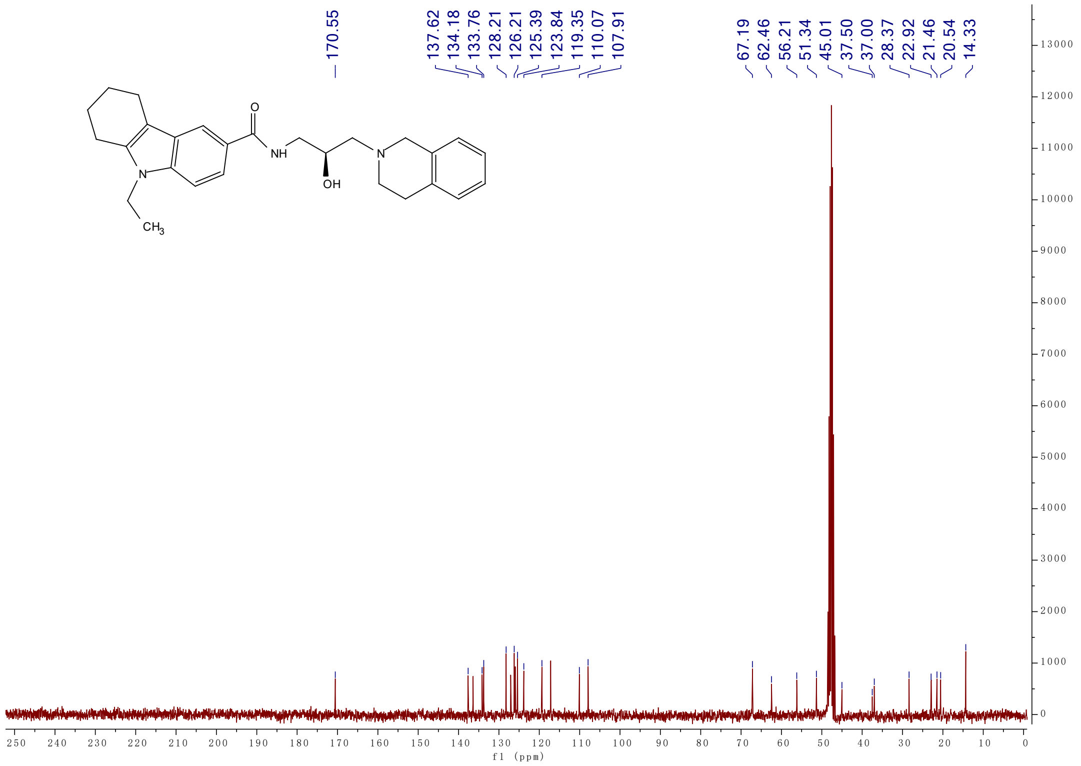

HRMS of compound **14**

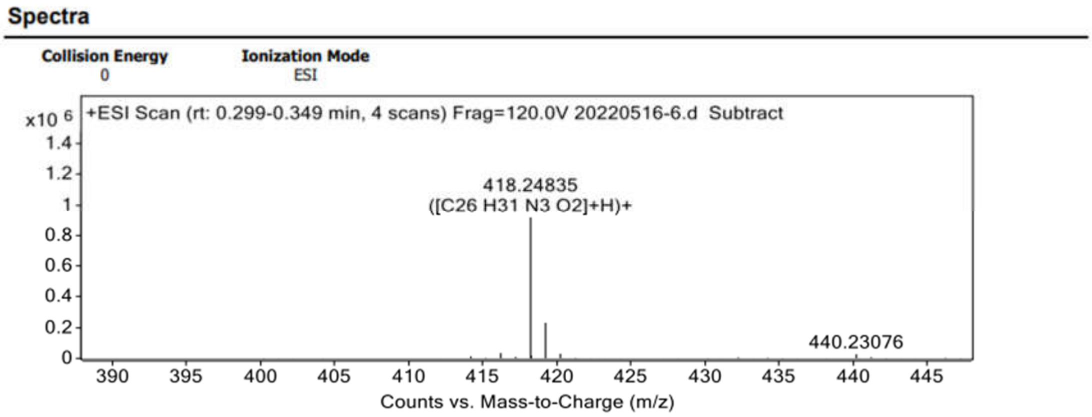

^{1}H-NMR of compound **15**

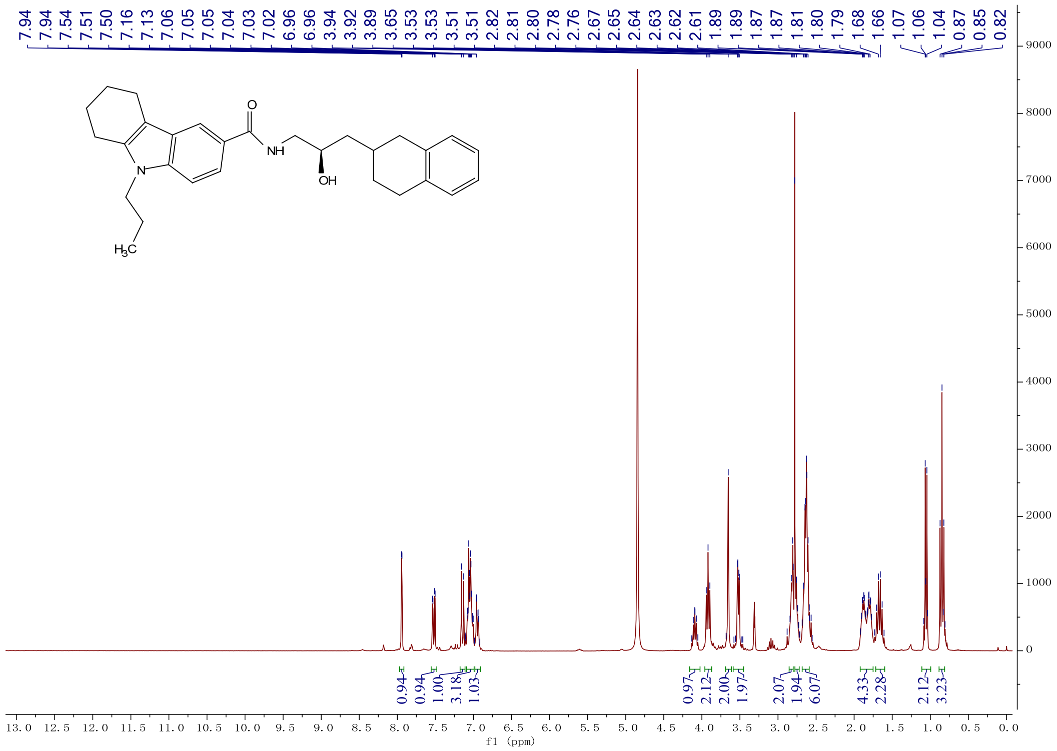

^{13}C-NMR of compound **15**

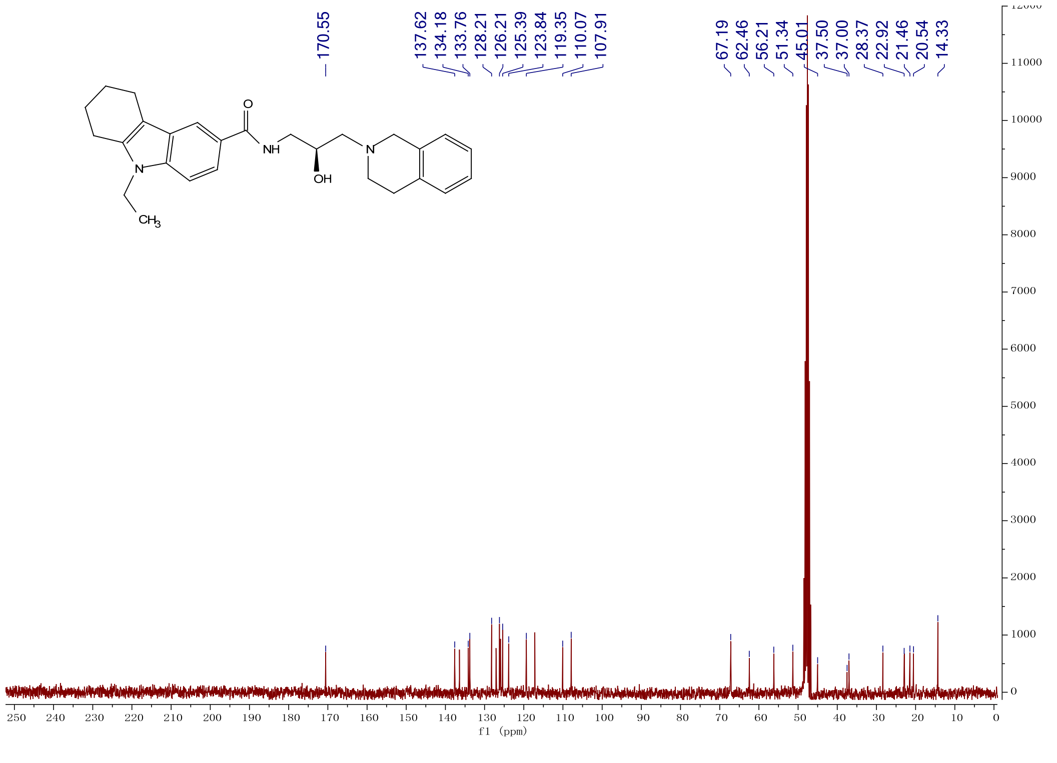

HRMS of compound **15**

Spectra

Collision Energy	Ionization Mode
0	ESI

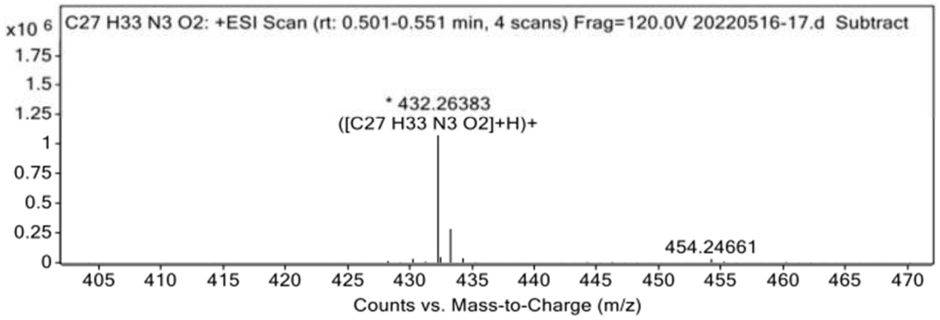

^{1}H-NMR of compound **16**

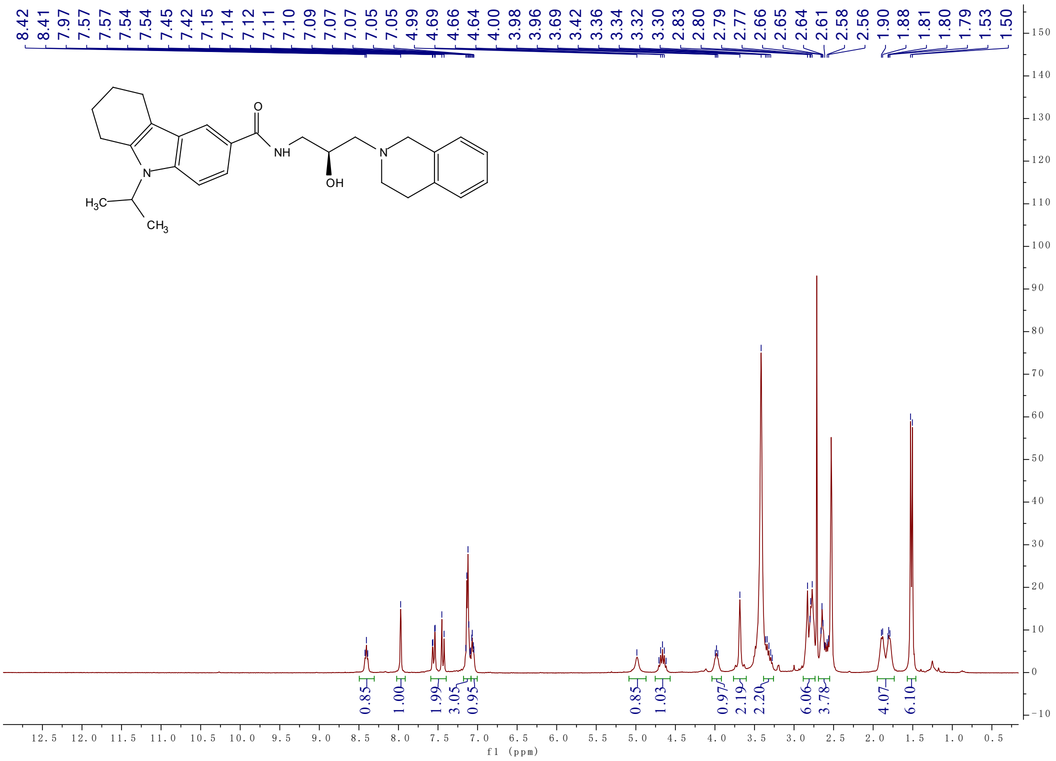

^{13}C-NMR of compound **16**

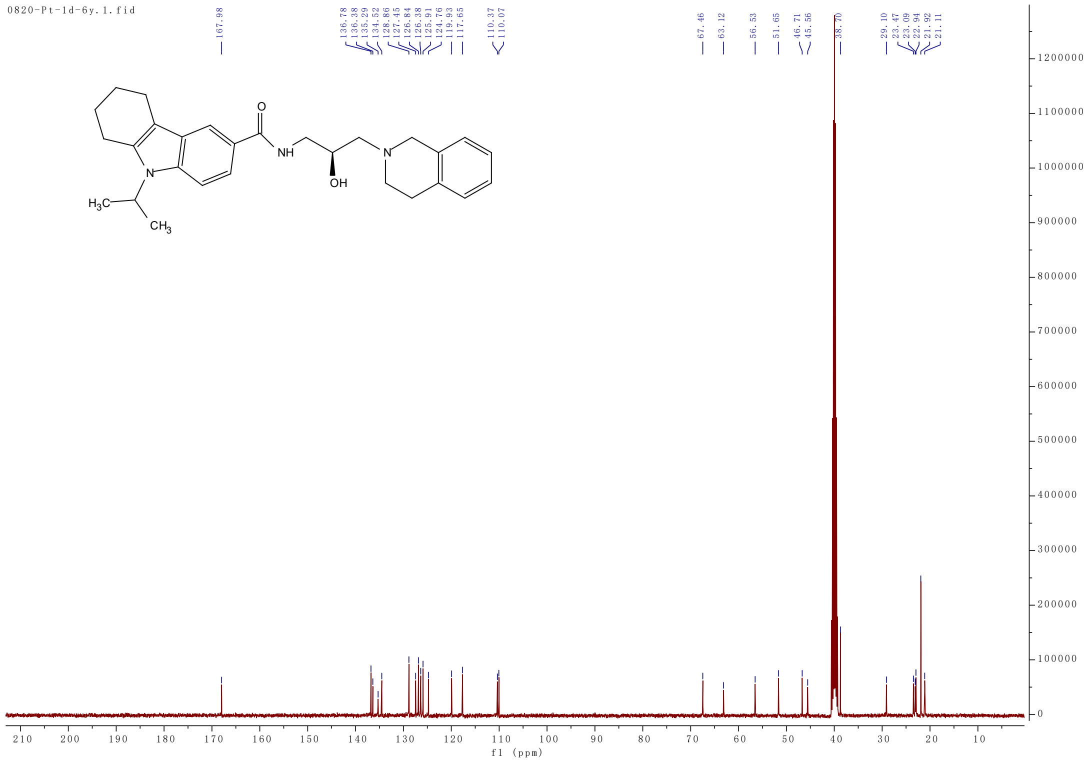

HRMS of compound **16**

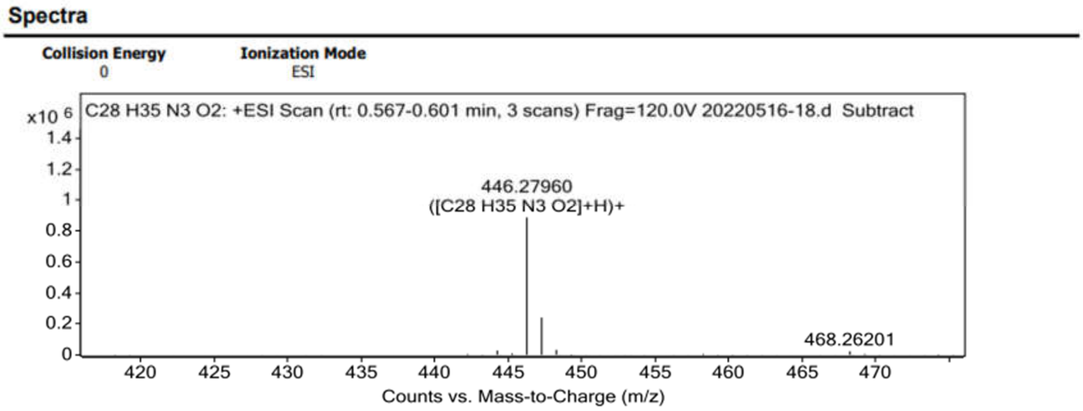

^{1}H-NMR of compound **17**

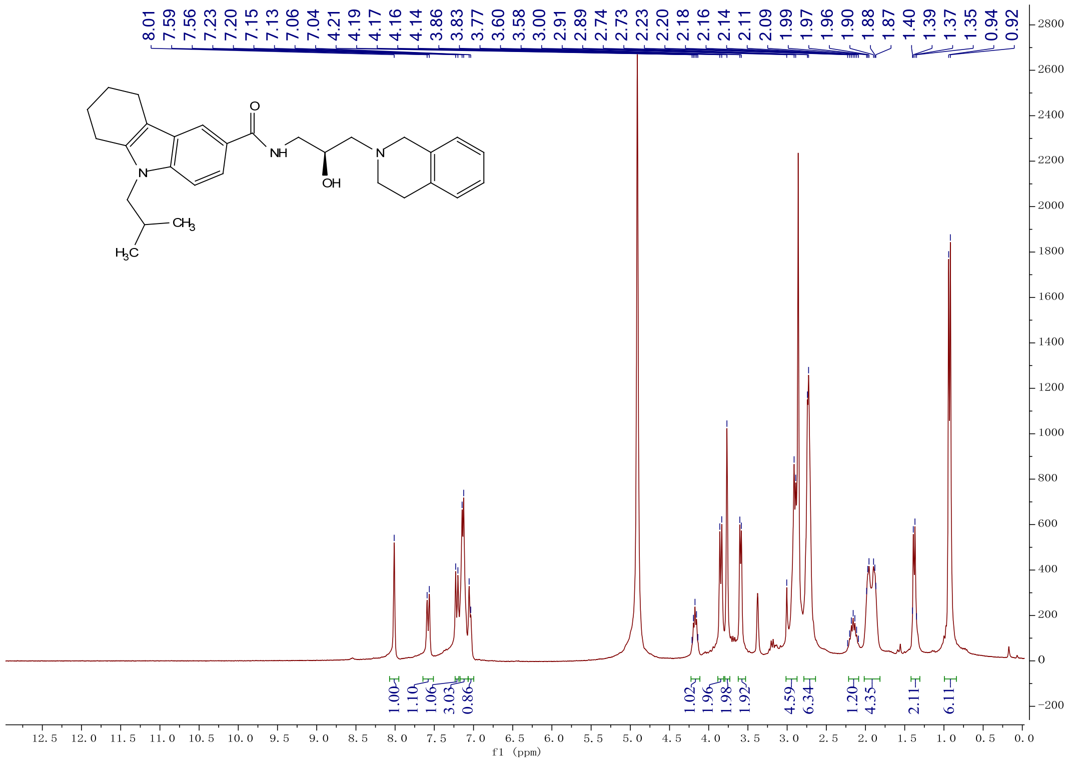

^{13}C-NMR of compound **17**

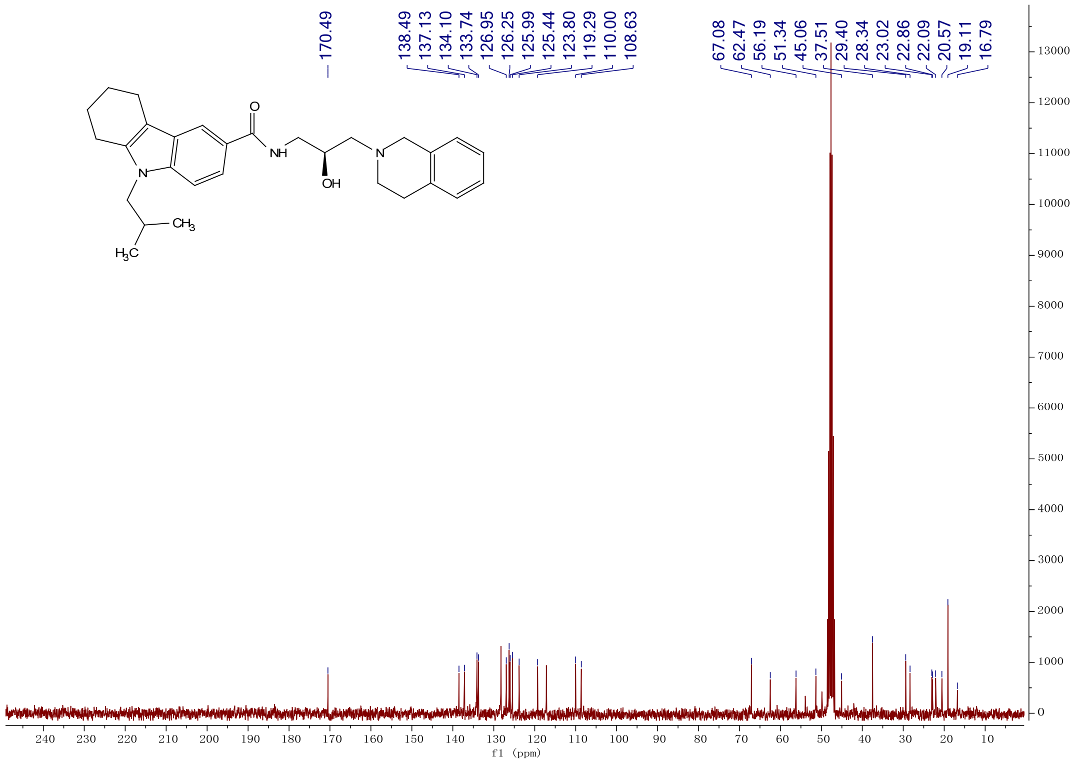

HRMS of compound **17**

Spectra

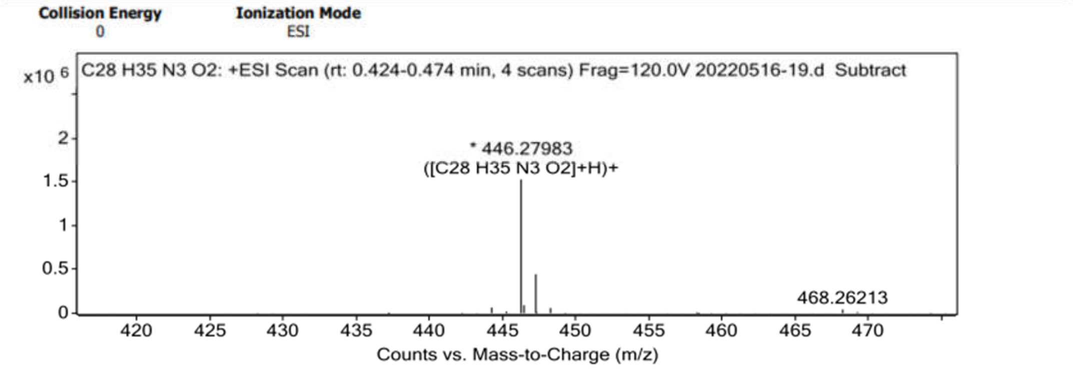

^{1}H-NMR of compound **18**

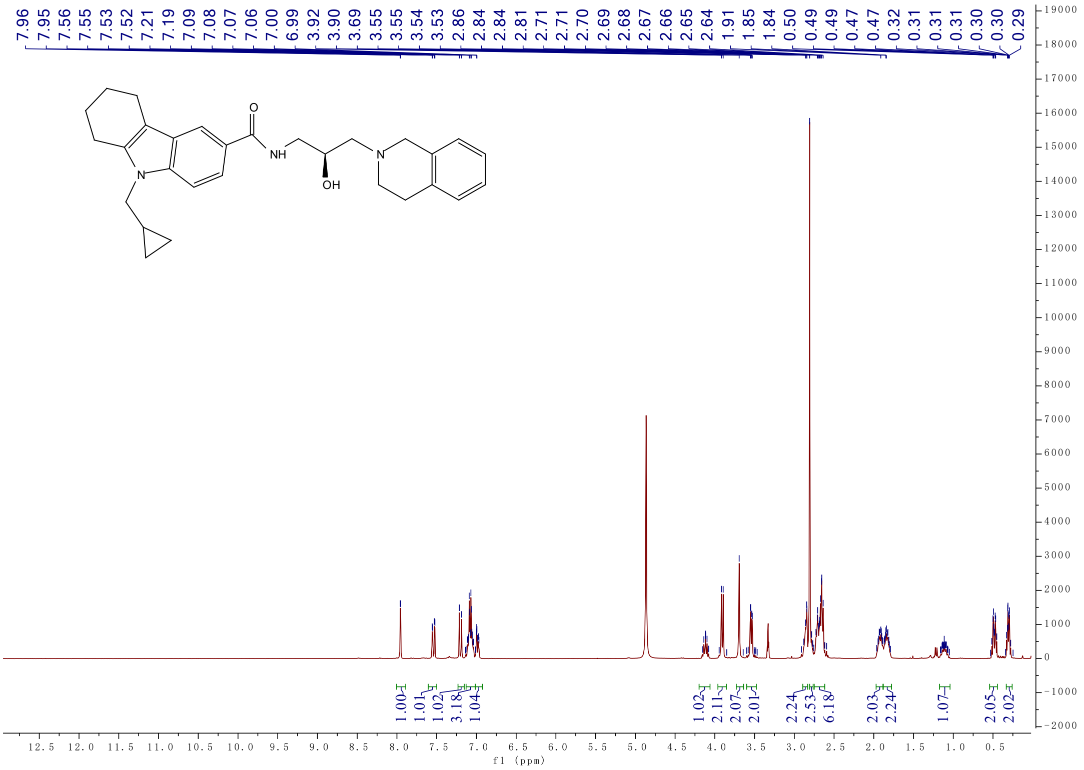

^{13}C-NMR of compound **18**

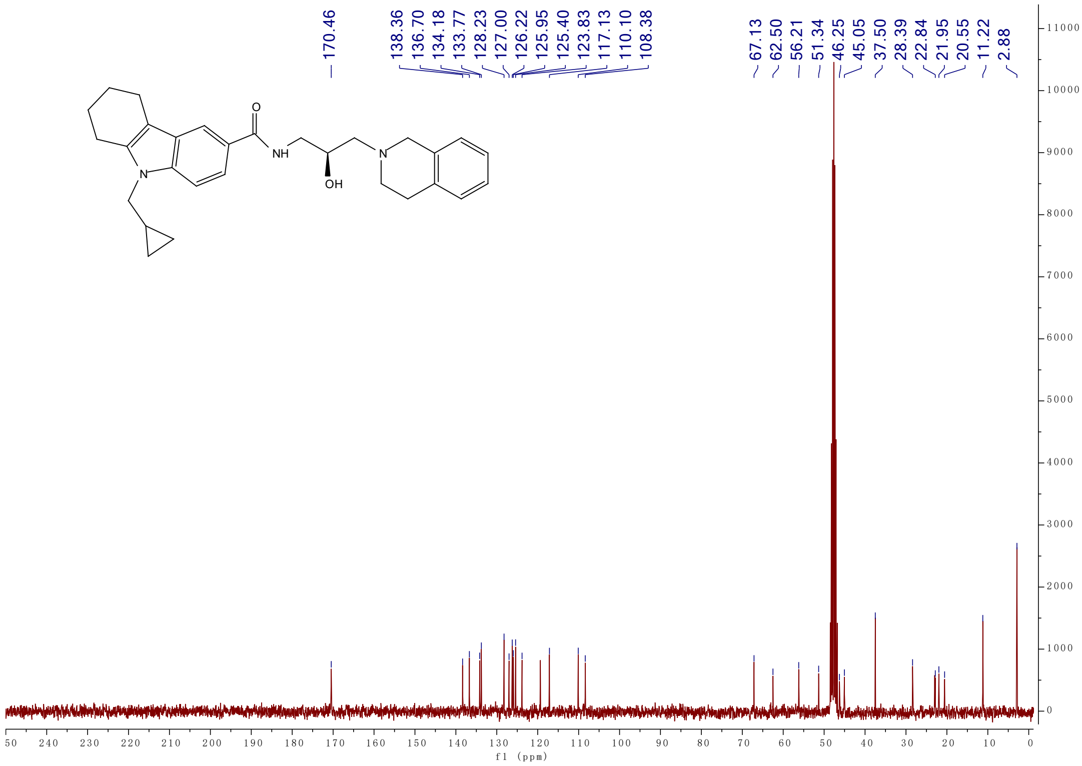

HRMS of compound **18**

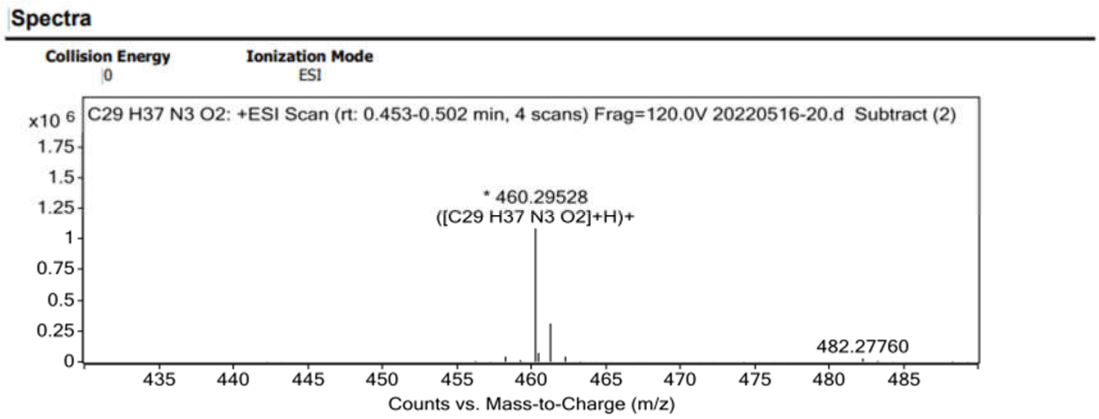

^{1}H-NMR of compound **19**

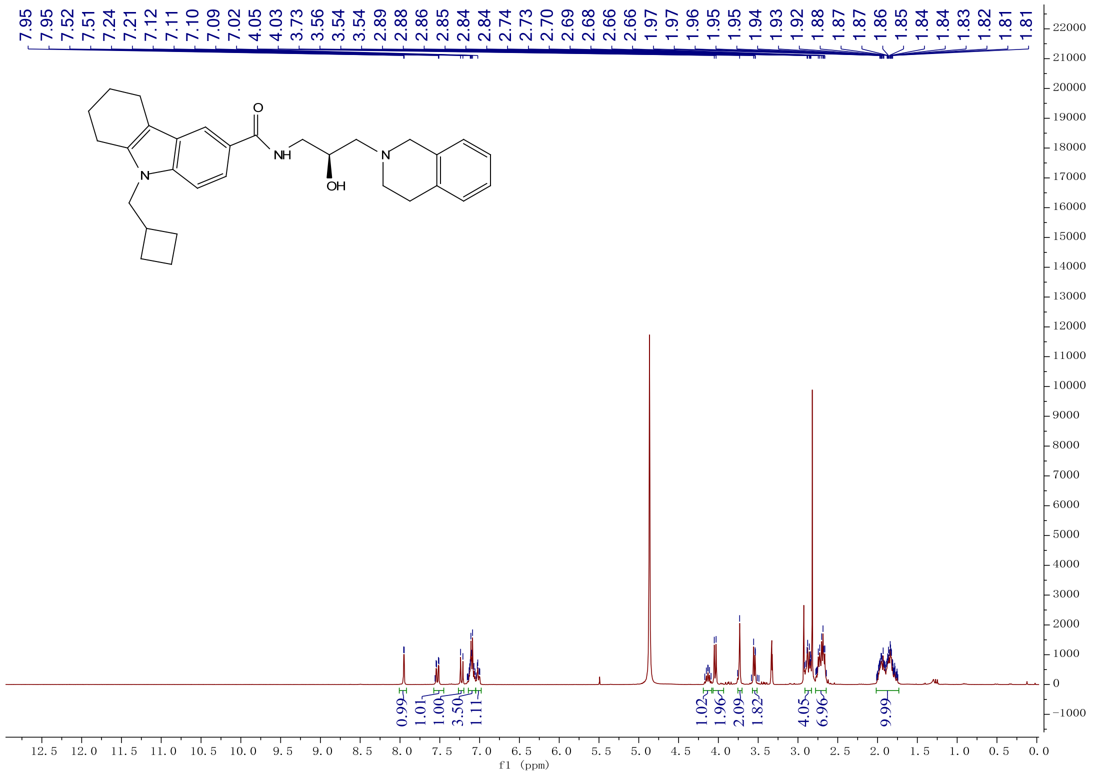

^{13}C-NMR of compound **19**

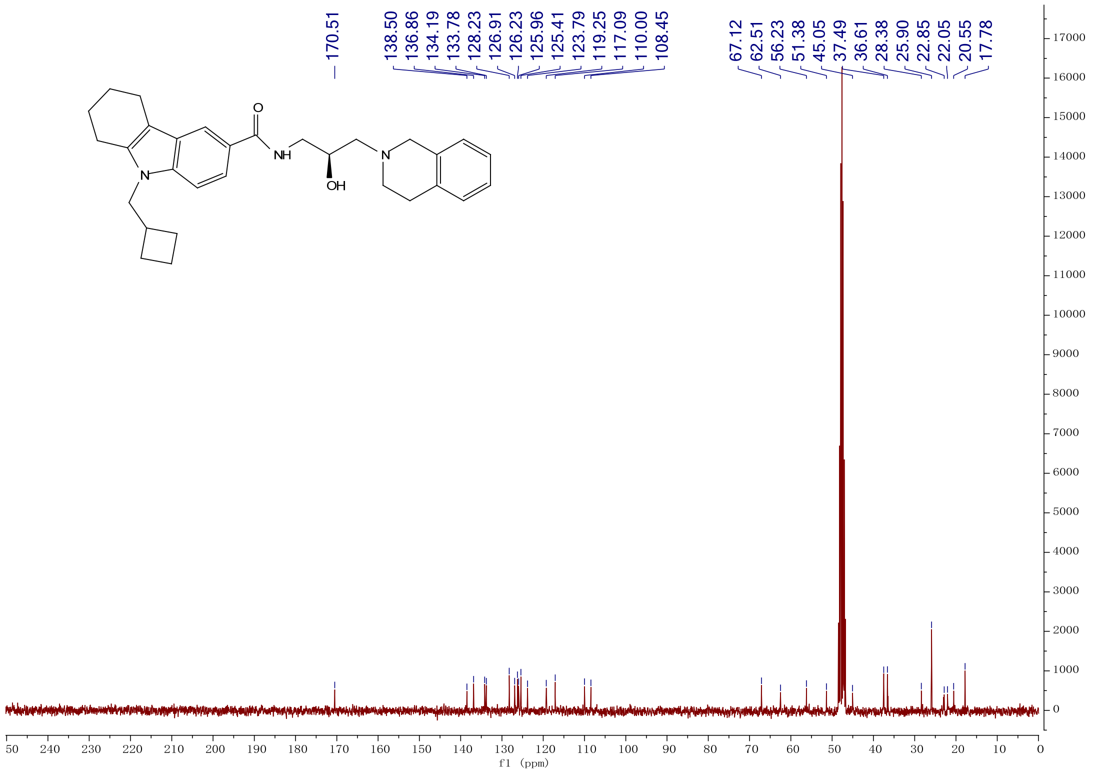

HRMS of compound **19**

Spectra

Collision Energy	Ionization Mode
0	ESI

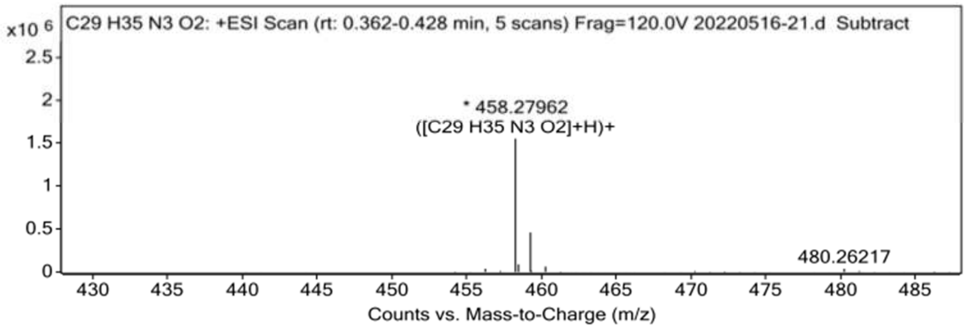

^{1}H-NMR of compound **20**

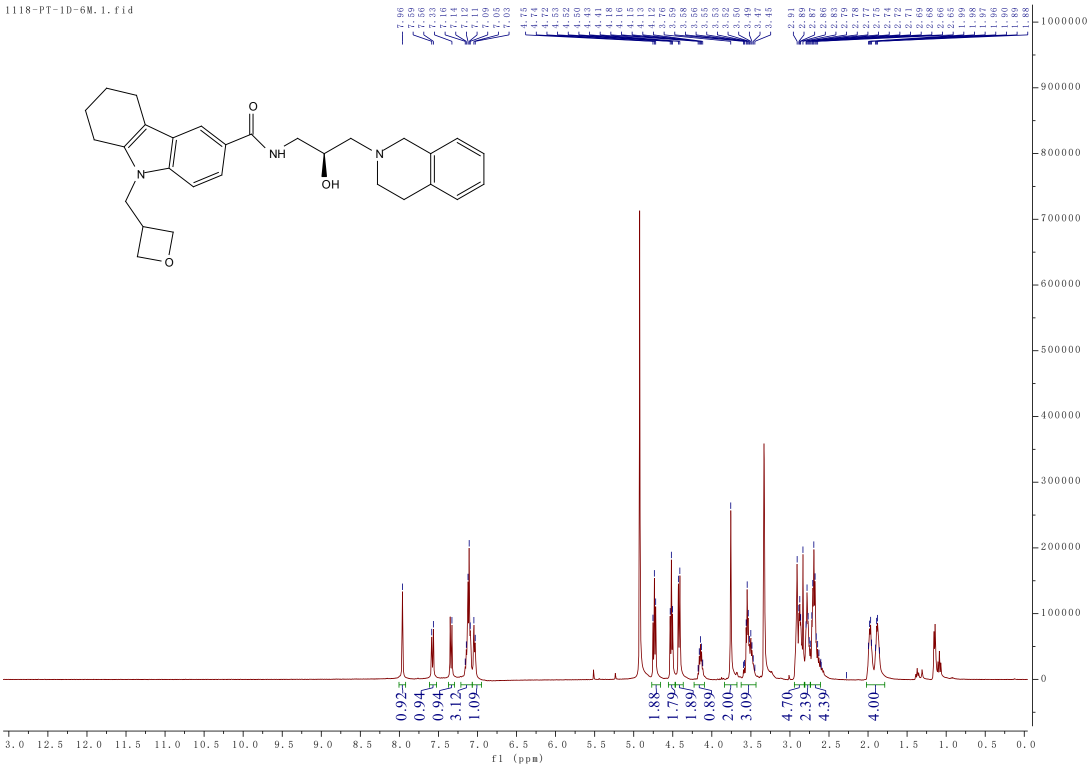

^{13}C-NMR of compound **20**

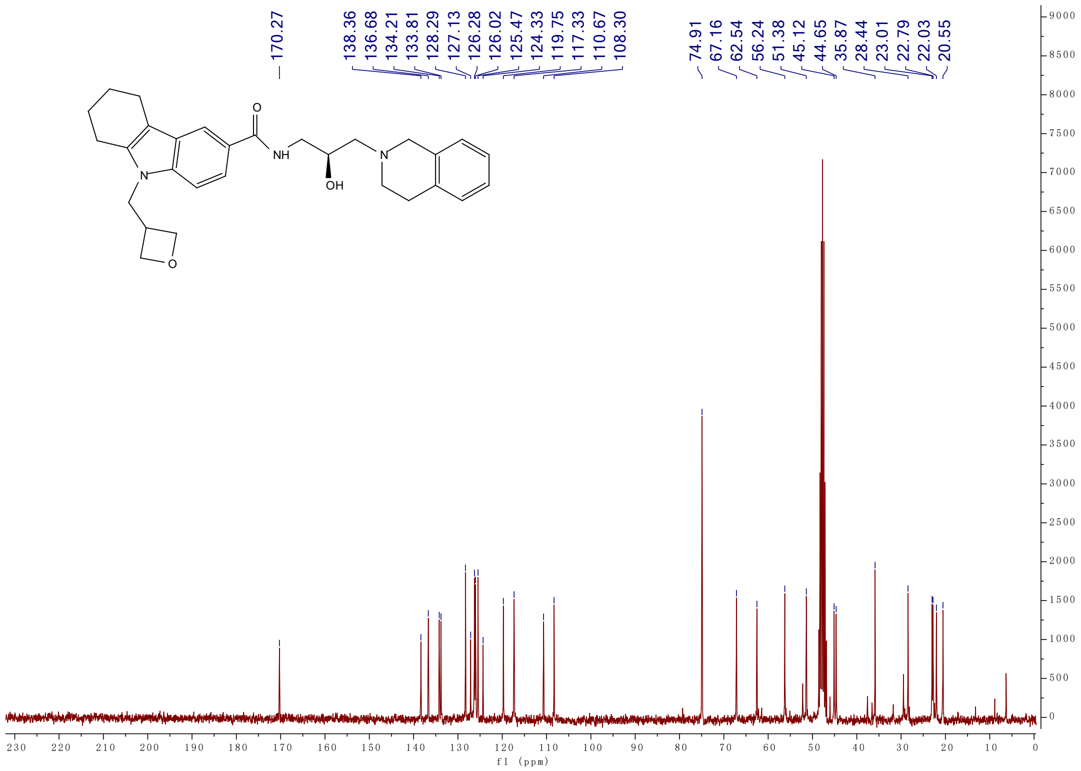

HRMS of compound **20**

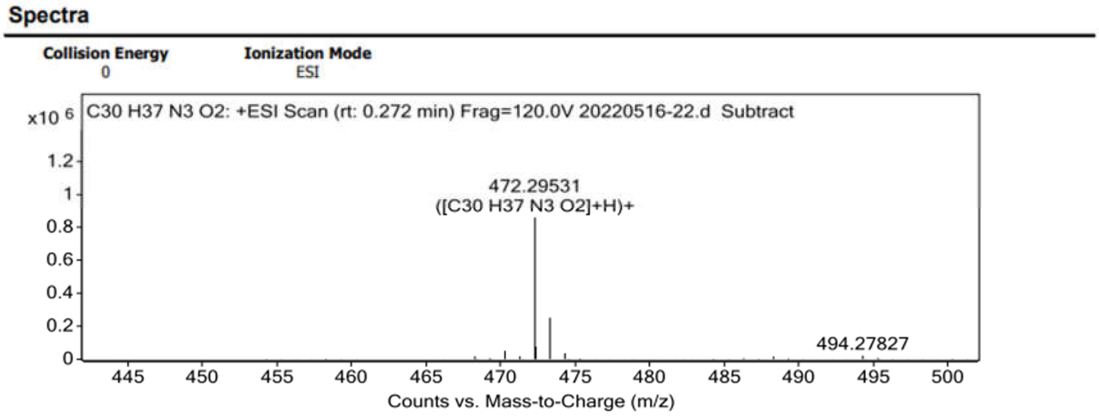

^{1}H-NMR of compound **21**

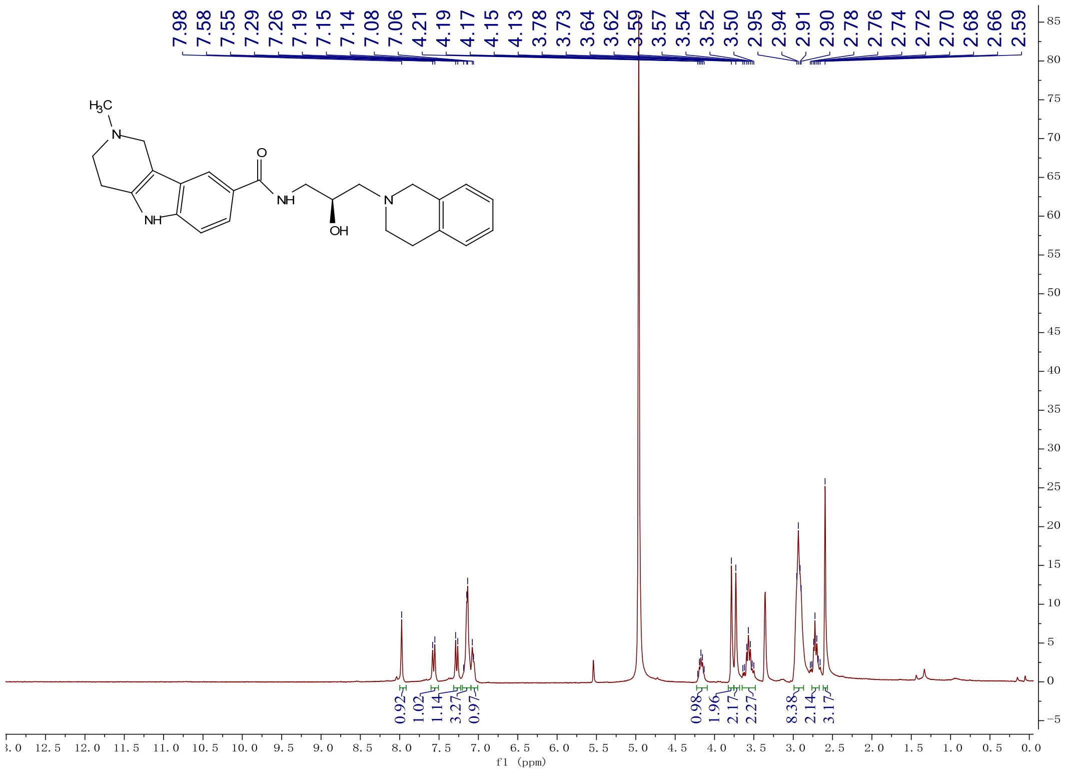

^{13}C-NMR of compound **21**

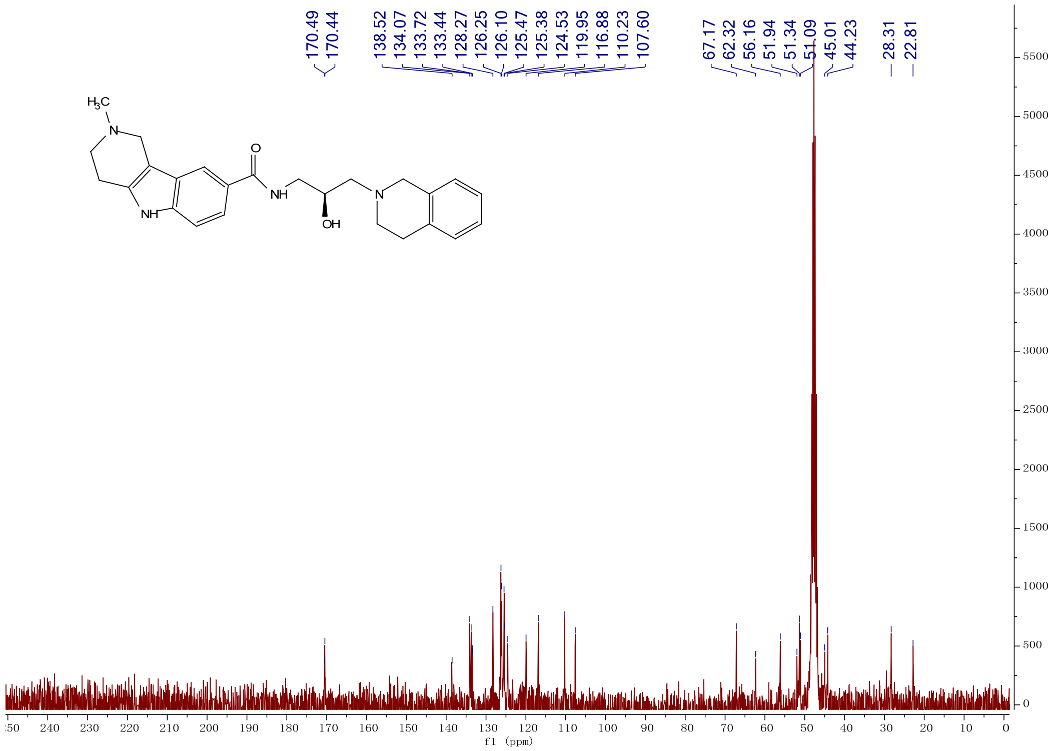

HRMS of compound **21**

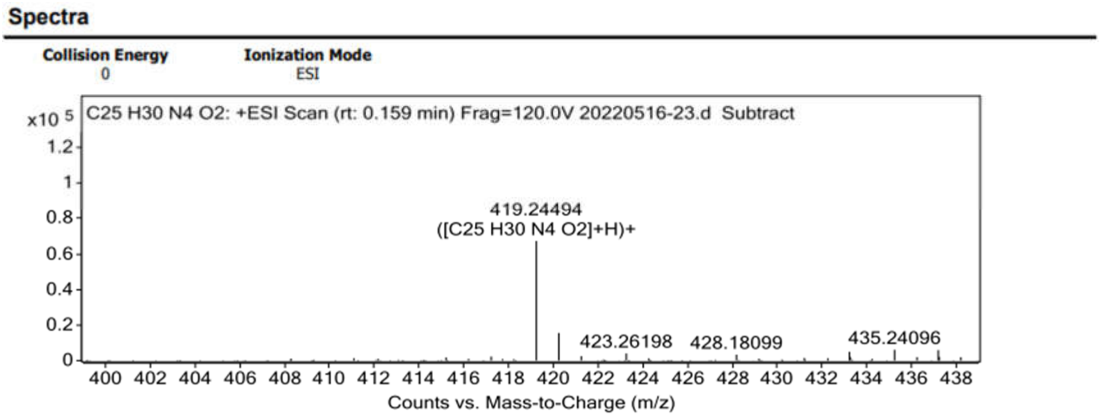

^{1}H-NMR of compound **22**

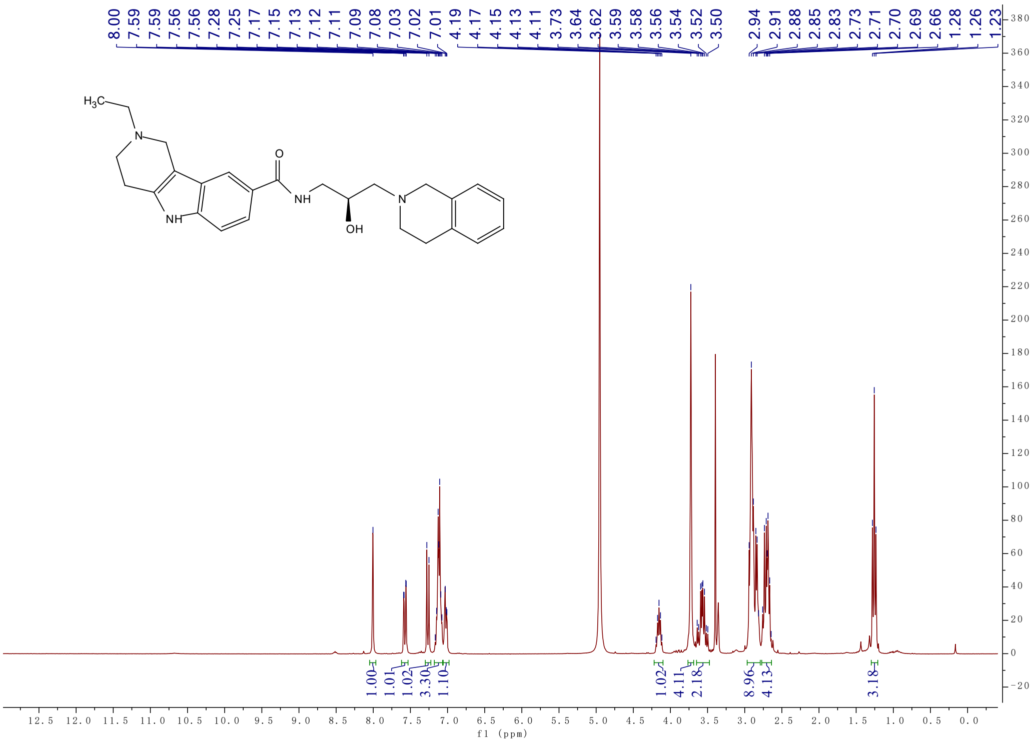

^{13}C-NMR of compound **22**

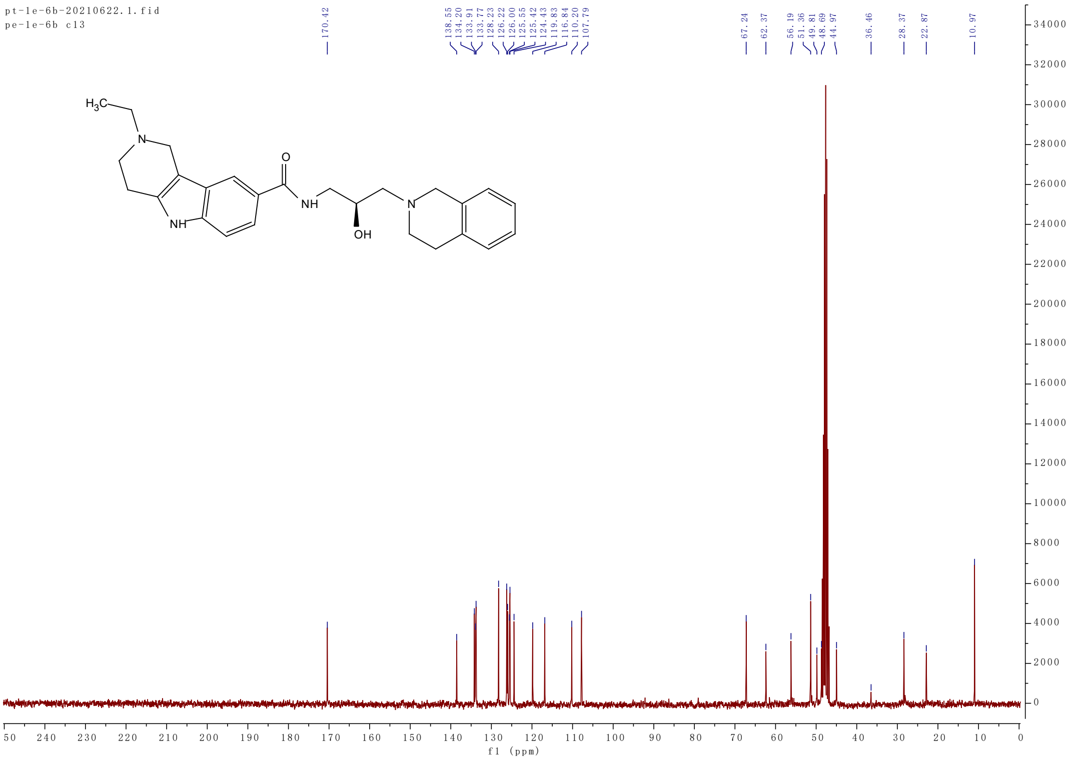

HRMS of compound **22**

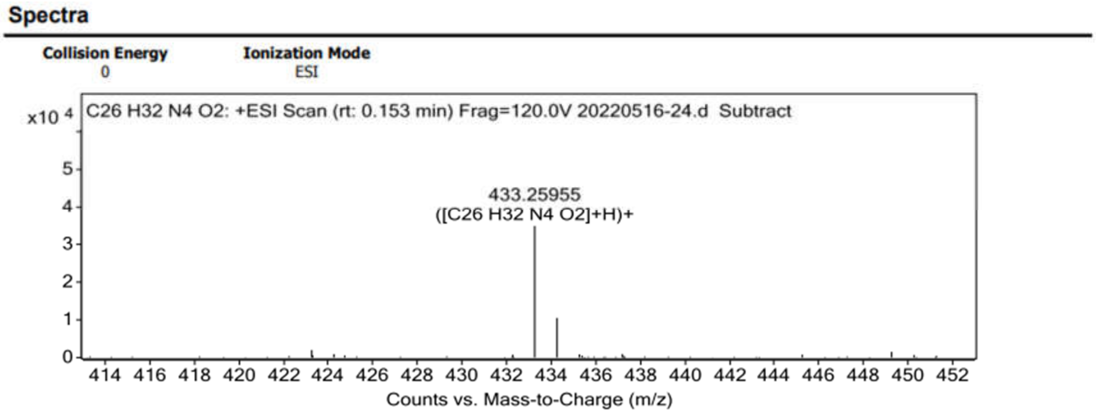

^{1}H-NMR of compound **23**

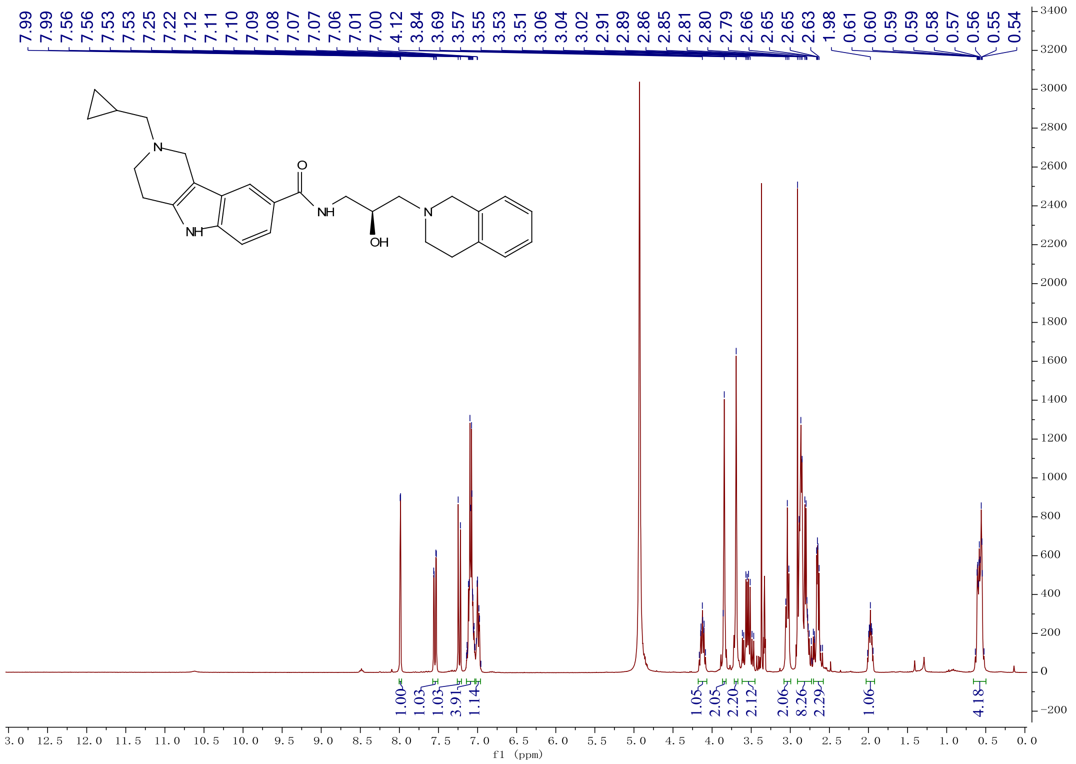

^{13}C-NMR of compound **23**

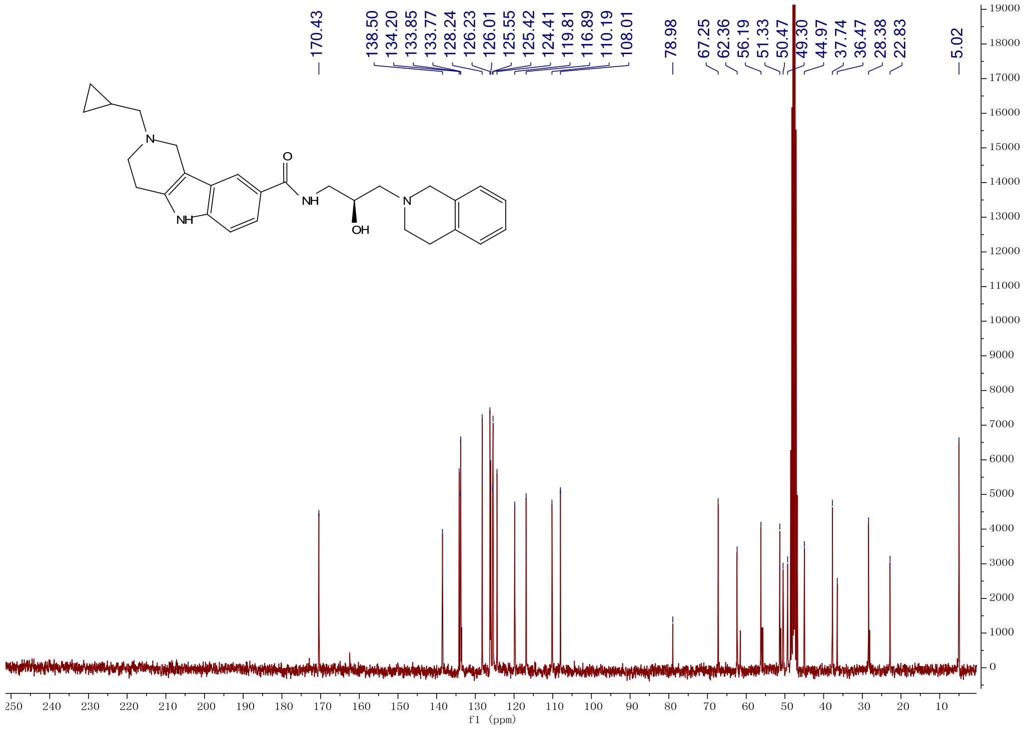

HRMS of compound **23**

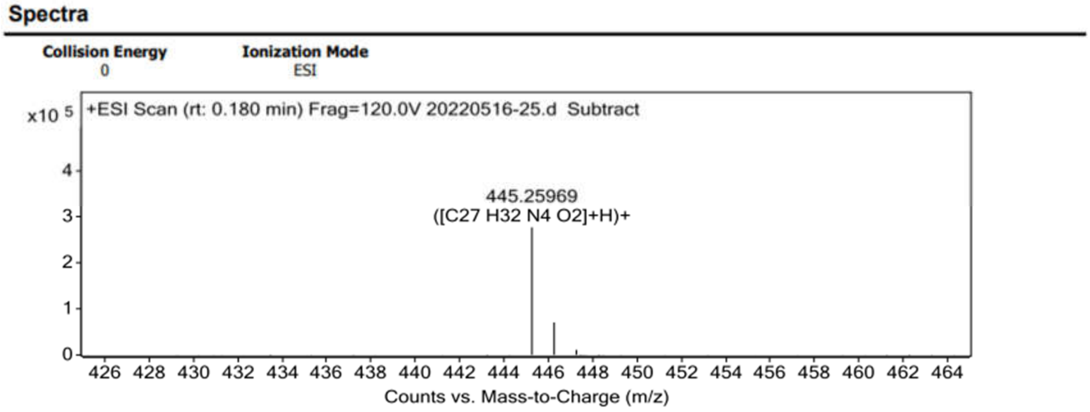

^{1}H-NMR of compound **24**

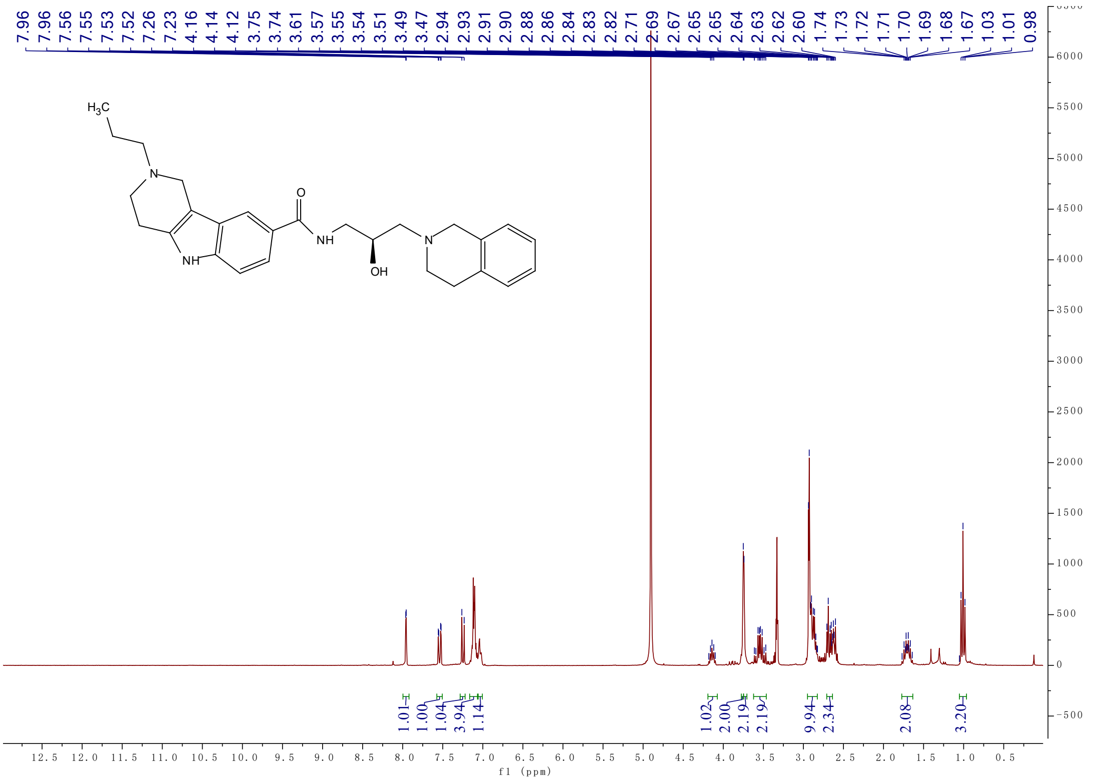

^{13}C-NMR of compound **24**

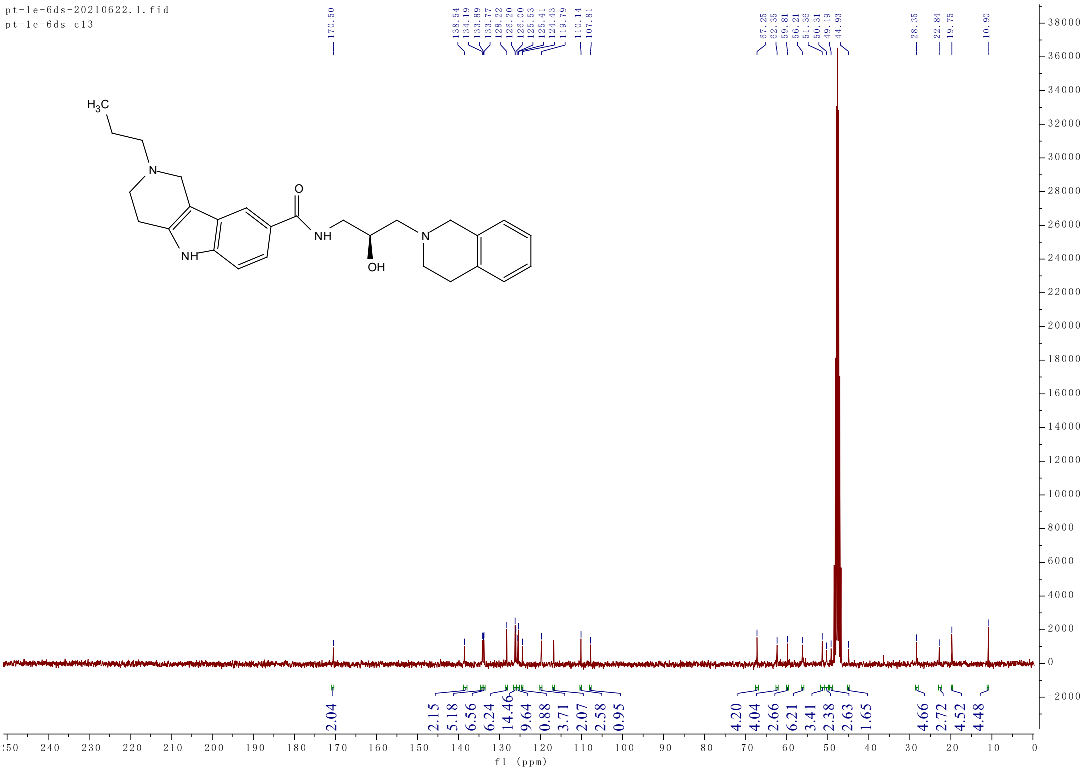

HRMS of compound **24**

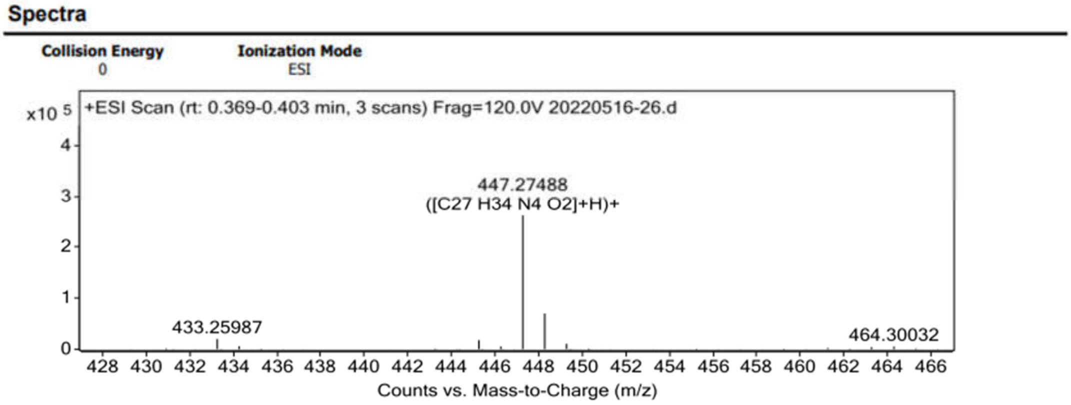

^{1}H-NMR of compound **25**

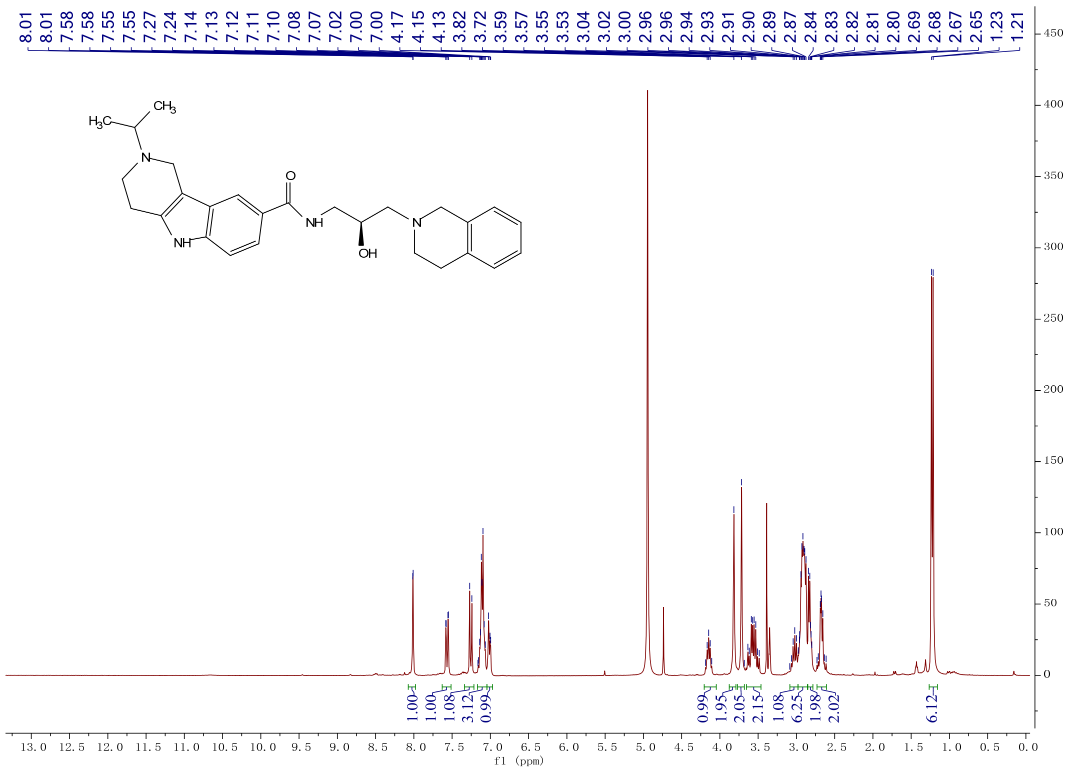

^{13}C-NMR of compound **25**

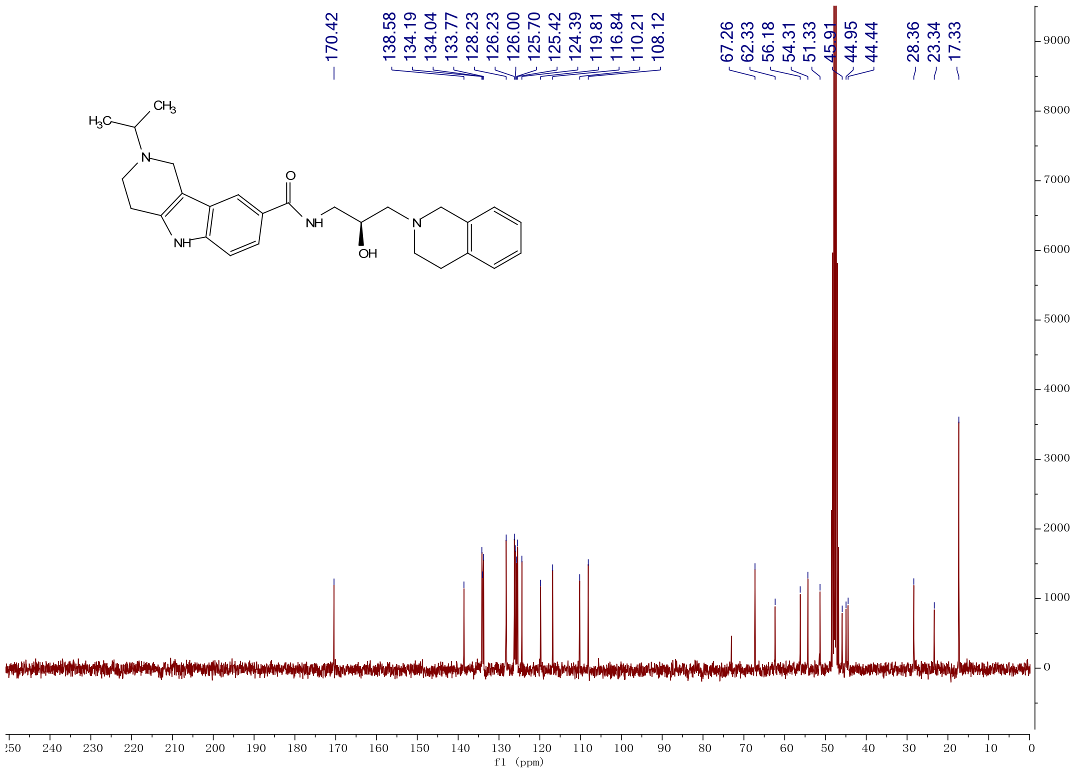

HRMS of compound **25**

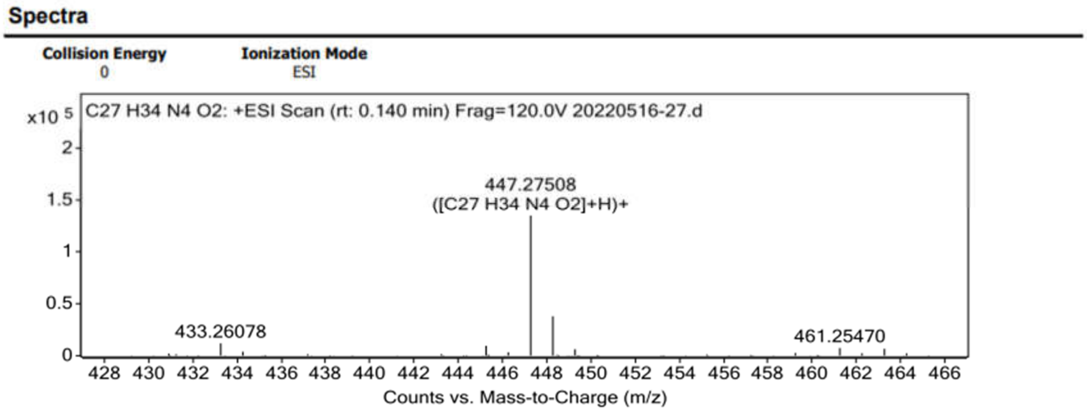

^{1}H-NMR of compound **26**

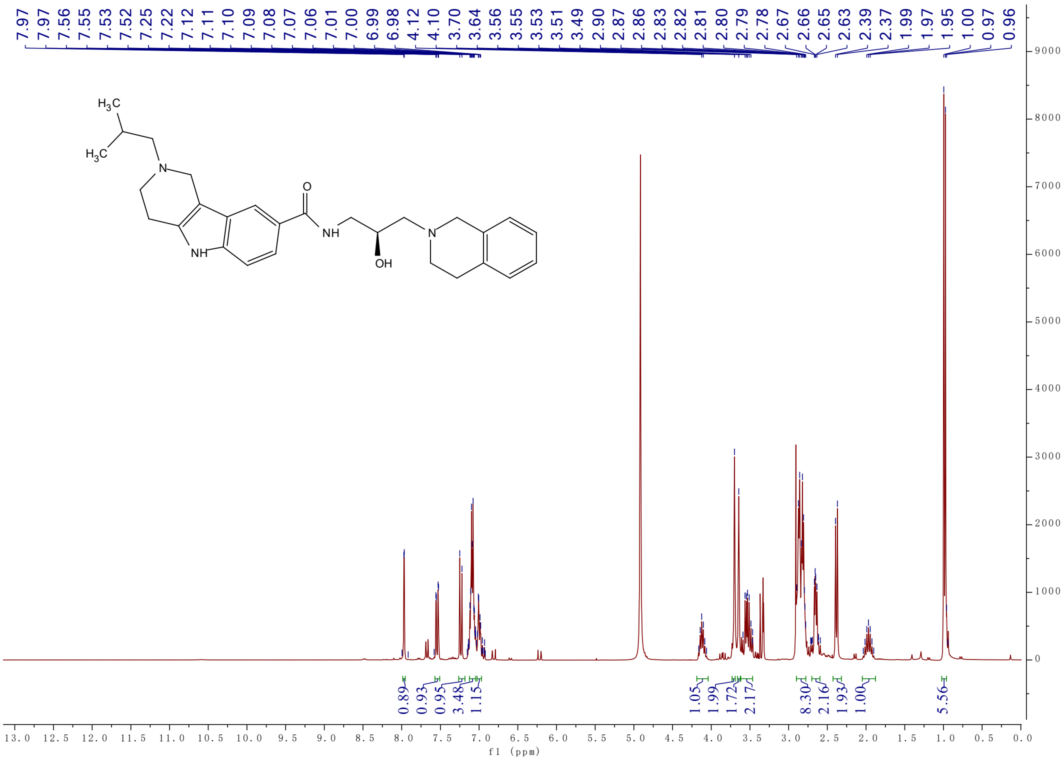

^{13}C-NMR of compound **26**

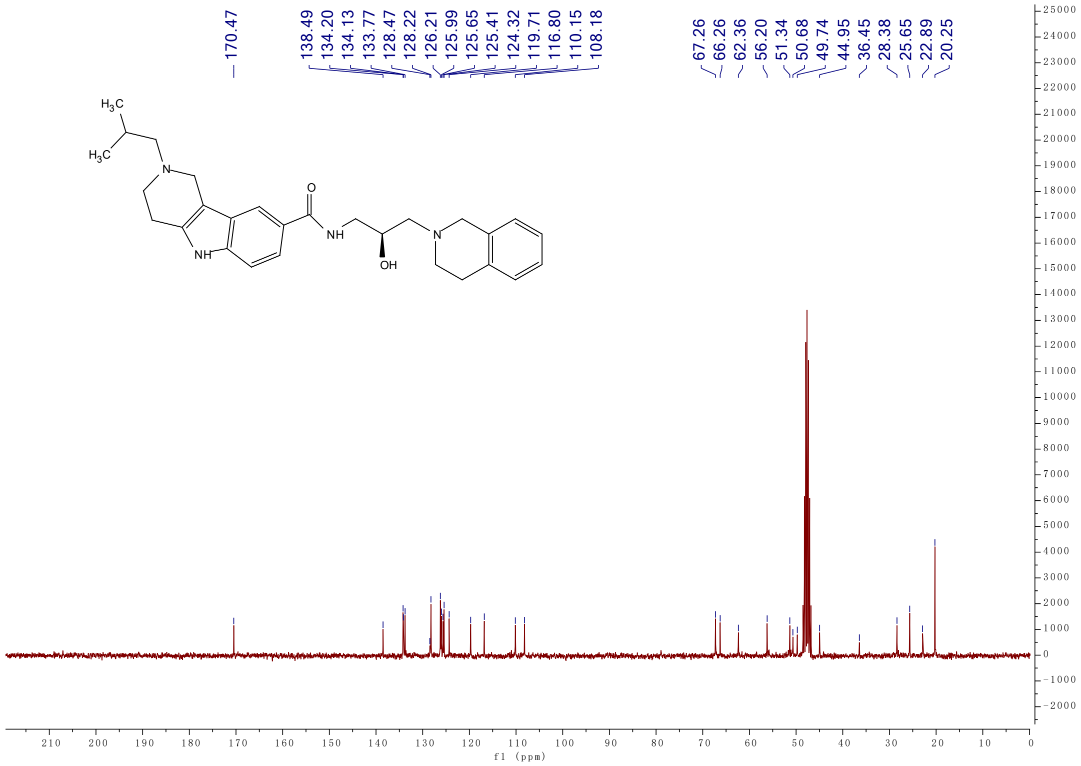

HRMS of compound **26**

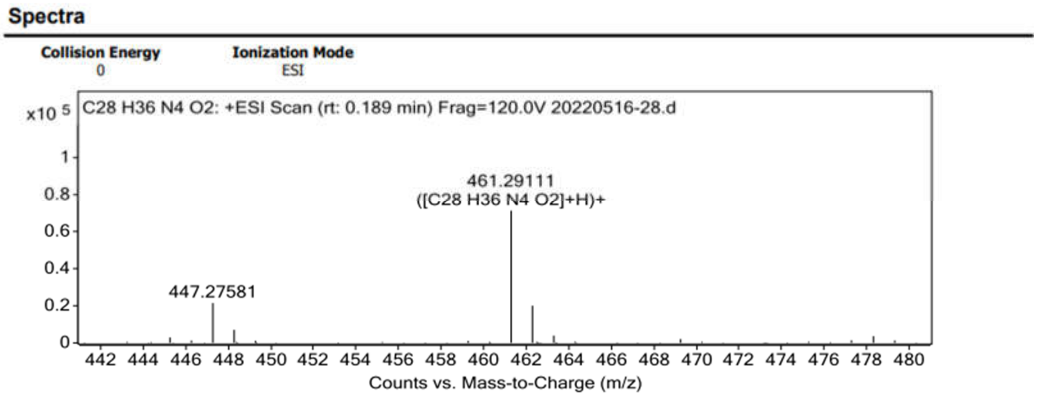

Supplement: Supplementary file 1 [file molecules-27-06637-s001.zip › molecules-1950072 -supplementary materials .pdf]
